# Ninhydrin as a Covalent Warhead for Chemical Proteomic-Enabled Discovery and Selective Engagement of Reactive Arginines

**DOI:** 10.1021/acscentsci.6c00922

**Published:** 2026-09-30

**Authors:** Andrew K. Ecker, Andreas Langen, Chloe S. Fields, José L. Montaño, Minh Tran, Ian B. Seiple, Balyn W. Zaro

**Affiliations:** † Department of Pharmaceutical Chemistry, 8785 University of California, San Francisco, San Francisco, California 94158, United States; ‡ Department of Chemistry, 683716 Scripps Research Institute, La Jolla, California 92037, United States; § Cardiovascular Research Institute, University of California, San Francisco, San Francisco, California 94158, United States

## Abstract

Covalent molecules have emerged as next-generation therapeutics and as powerful tools for perturbing fundamental biological processes. Chemical proteomic methods to screen for reactive proteinaceous amino acids have transformed small-molecule discovery pipelines, but their application remains mostly limited to sites where reactive cysteines and lysines are present. Here we report a ninhydrin-based warhead that selectively modifies arginine residues, thus expanding the repertoire of amino acids targetable by covalent molecules. Specifically, we developed alkyne-functionalized variants of ninhydrin to establish an arginine-specific chemical proteomics platform, enabling the classification of more than 6,800 unique reactive arginines from one cell line. These studies uncovered potential modification sites on disease-relevant proteins, including reactive arginines within catalytic sites that are essential for function and reactive arginines on proteins lacking reactive cysteines. By endowing a reversible small-molecule binder of cyclophilin A with a ninhydrin warhead, we achieved selective, covalent engagement in cell lysates and attenuation of enzymatic activity *in vitro*. These findings establish ninhydrin as a warhead for studying arginine reactivity and identifying at scale arginines amenable to covalent modification.

## Graphical abstract

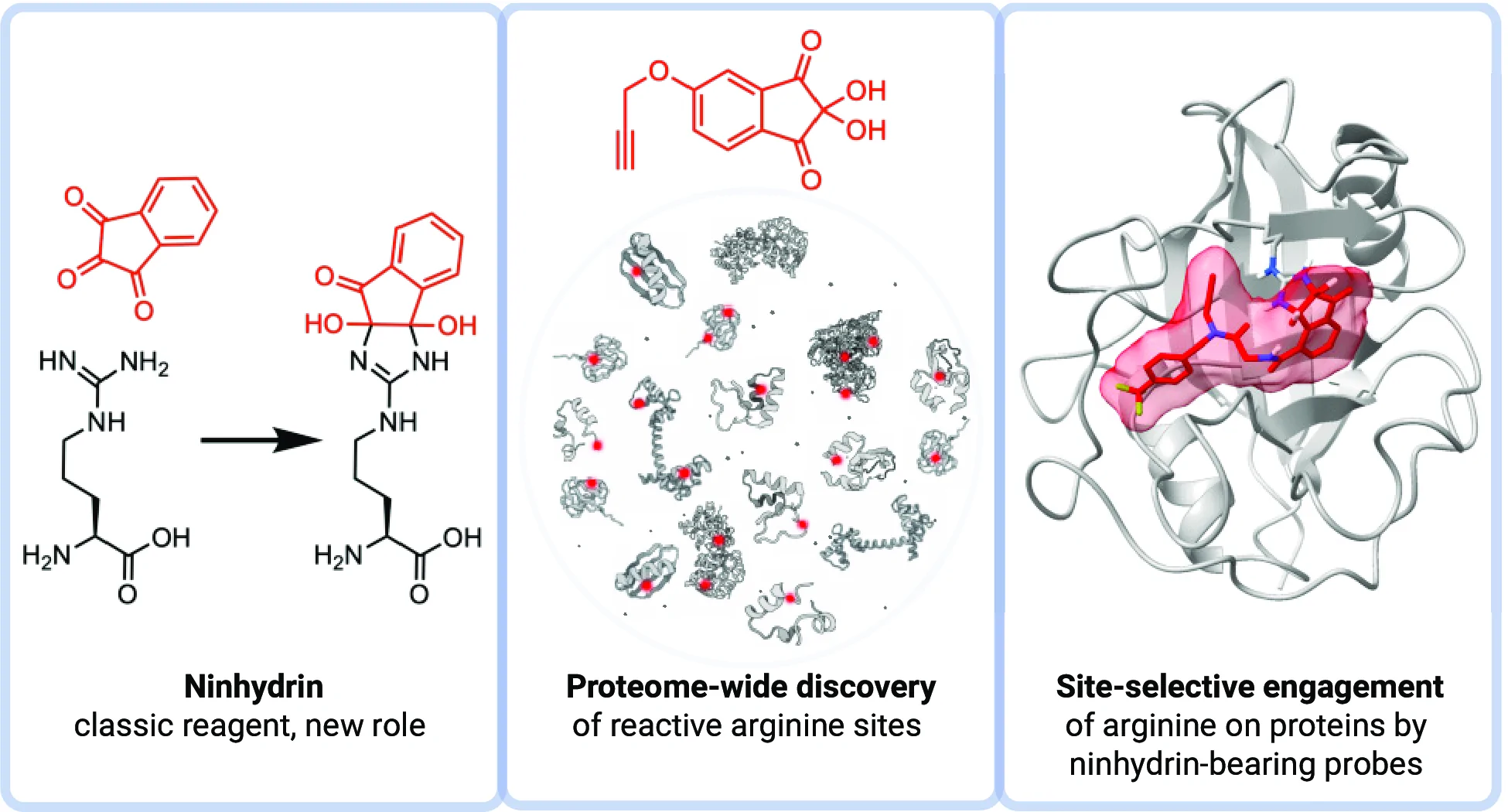

Covalent therapeutics have revolutionized drug discovery by enabling precise and irreversible engagement of protein targets. Cysteine has been the predominant residue leveraged for covalent modifications, with approved drugs such as ibrutinib (BTK Cys481), afatinib (EGFR Cys797), and adagrasib/sotorasib (KRAS G12C) exemplifying this strategy. Evaluating and exploiting proteome-wide cysteine reactivity has been transformed by the use of broad-spectrum electrophiles, such as iodoacetamide-alkyne (**IA-Alk**), that enable both reactivity profiling and competitive screening to assess a molecule of interest’s selectivity.^1,2^ More recently, lysine residues have also emerged as attractive covalent drug targets, aided by probes that form imines or related adducts.^3–5^ Beyond warheads selectively targeting a specific amino acid residue, such as cysteine and lysine, activity-based protein profiling (ABPP) strategies have begun to extend toward other nucleophilic side chains, although these remain less comprehensively mapped. SuFEx-based probes also enable the profiling of lysines and less conventional nucleophilic residues across the proteome, including histidine and tyrosine.^6–9^

Arginine plays indispensable roles in enzymatic catalysis, molecular recognition, and nucleic acid interactions. The positively charged guanidinium side-chain engages in a diverse array of noncovalent interactions, including salt-bridging and cation-π contacts, enabling arginine to stabilize transition states, mediate protein–protein interaction, and recognize aromatic functional groups within biomolecular targets.^10^ However, arginine’s high p*K*_a_, resonance-stabilized charge distribution, and limited nucleophilicity complicate the development of covalent modification strategies to detect free, reactive arginines. The reaction between vicinal dicarbonyl compounds with amidine and guanidine moieties has long been investigated from initial studies by Diels and Schleich^11^ to the Maillard reaction of sugar fragments with arginine,^12^ yet the direct application of these reactions in biological systems has been quite limited. α,β-diketoamides^13^ and phenylglyoxal derivatives^14–19^ have been explored. Phenylglyoxals were found to have significant cross reactivity with lysines when used as a probe for activity-based protein profiling studies.^17^ An anthracene moiety is also reported to engage arginines, resulting in a carbonylation scar that can be derivatized for indirect detection of anthracene-modified arginine residues.^20^ Chemoproteomic workflows using α,β-diketoamides as warheads have not yet been reported. The development of a selective and robust warhead for covalent pan-arginine modification would provide a powerful tool for studying arginine reactivity and function in complex biological contexts and expanding the repertoire of covalently targetable residues.

Ninhydrin (**Nin**), an indanetrione, has long been utilized for detecting amino acids through its chromogenic reaction with primary amines at high temperature. Despite its established role in analytical chemistry, its potential as a selective covalent modifier of arginine has remained underexplored. Given its electrophilic properties and ability to rapidly form stabilized cyclic adducts with guanidine,^21^ we hypothesized that **Nin** could be repurposed for targeted covalent modification of arginine in biological systems. Furthermore, we postulated that if **Nin** were reactive enough across proteinaceous arginine residues, further functionalization with a bioorthogonal handle, such as an alkyne, would allow for the development of a pan-arginine chemical proteomics platform akin to **IA-Alk** and the isoTOP-ABPP workflow, as developed by Cravatt and co-workers for cysteine.^1,22^

## *In Vitro* Characterization of Arginine Engagement of Ninhydrin

Many vicinal dicarbonyl compounds in aqueous environments exist predominantly in their hydrate state, which attenuates electrophilicity. The equilibrium between this hydrate state and the reactive dehydrated state governs their ability to engage in covalent modification.^23^ To investigate this equilibrium for **Nin** versus the known arginine-reactive **phenylglyoxal** (**PGO**),^24^ we performed density functional theory (DFT) calculations (Figure 1A). These calculations estimate the relative thermodynamic preference for each state and thus the baseline population of the electrophilic, dehydrated species. Here, K_eq_ is defined as the dimensionless equilibrium constant for the dehydration reaction, the ratio of dehydrated to hydrated species. Our calculations revealed that both probes overwhelmingly favor their hydrate form; however, **Nin** has a calculated K_eq_ of 3.55 × 10^–3^, indicating a higher fraction of the molecule resides in the reactive dehydrated state. **PGO** has a significantly lower calculated K_eq_ of 1.33 × 10^–6^ (Figure 1A). This difference suggests that under identical conditions, **Nin** maintains a greater accessible pool of the electrophile. Although the calculated hydrate/dehydrate equilibrium provides a useful explanation for the greater labeling efficiency of ninhydrin, it should not be interpreted as a static measure of probe reactivity. Arginine labeling proceeds through the dehydrated electrophile, and covalent capture of this species is expected to draw continuously from the hydrated reservoir. The DFT-derived equilibrium difference may predict initial concentration of the reactive electrophile, but labeling also depends on the kinetics of arginine attack and cyclic adduct formation and stability. Therefore, we view hydration equilibrium as one potential contributor to a reactivity difference between **Nin** and **PGO**, rather than a complete explanation.

**Figure 1:**
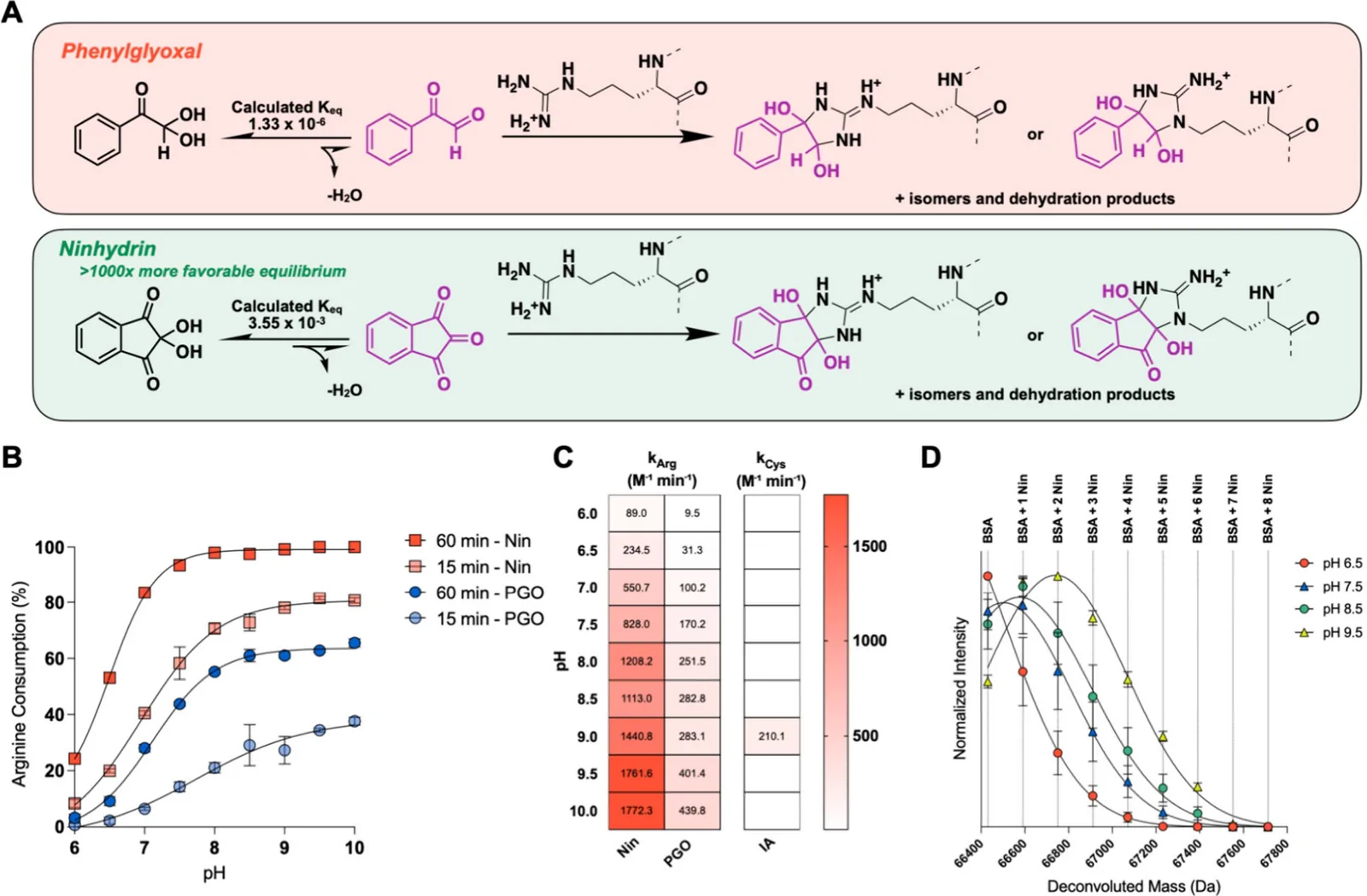
| Profiling the reactivity of ninhydrin with arginine in vitro. **A.** Proposed reactions between **phenylglyoxal** (**PGO**) and **ninhydrin** (**Nin**) with arginine, highlighting differences in hydration equilibrium. Calculated equilibrium constants (K_eq_) were obtained by density functional theory (DFT) at the B3LYP/6–311G** level with implicit water solvation. **B.** Kinetic analysis of pH- and time-dependent consumption of Fmoc-Arg (500 μM) by **Nin** or **PGO** (15 mM, 30x) in PBS at 20 °C. Arginine consumption was quantified by LC-MS over 2 to 60 min. Error bars represent SD. Representative HPLC traces in SI. *n* = 4 **C.** Heatmap depicting the second order reaction kinetics of **Nin** or **PGO** with Fmoc-arginine determined by UHPLC analysis compared to the reaction of iodoacetamide with cysteine determined by Ellman’s reagent. **D.** Intact protein mass spectrometry analysis of BSA (1.5 μM) treated with 150 μM **Nin** at 37 °C for 30 min across pH 6.5 to 9.5. Reactions were quenched by dilution into 0.1% aqueous TFA and analyzed by LC–MS on a Agilent 6230 TOF System. Error bars represent SEM *n* = 3.

To directly evaluate reactivity with arginine, we used an HPLC-MS-based assay monitoring the consumption of Fmoc-Arg-OH (Figures 1B and 1C). The second order reaction kinetics were evaluated across a pH gradient ranging from 6.0 to 10.0, with time points recorded at 2, 7, 15, 30, and 60 min. At pH 7.5, complete consumption of free Fmoc-Arg-OH was observed within 60 min (k_Arg, pH 7.5_ = 828.0 ± 86.8 M^–1^ min^–1^), whereas in more basic conditions, reaction completion was achieved within 30 min (k_Arg, pH 9.5_ = 1761.6 ± 1.2 M^–1^ min^–1^) (Figures 1C and S1A). The relatively fast second order reaction kinetics are comparable to that of iodoacetamide with cysteine (*k*_cys, pH 9.0_ = 210.1 ± 30.2 M^–1^ min^–1^) and further supported our hypothesis that **Nin** may be a warhead amenable to engaging arginines in biological systems on a reasonable time scale.^25^

When we compared these rates to the reaction of **PGO** toward arginine, we found, on average, a 6-fold decrease in reactivity (k_Arg, pH 7.5_ = 170.2 ± 0.2 M^–1^ min^–1^), with **PGO** never fully consuming the Fmoc-Arg-OH (Figures 1C and S1B). A similar study was performed with **Nin** toward Fmoc-Lys-OH and glutathione,^26^ which did not result in appreciable engagement (Figures S1C and S1D). Reversible lysine-reactive warheads such as salicylaldehydes that form Schiff base adducts require chemical reduction to be stabilized for proteomic detection.^5^ **PGO**-based workflows to modify arginine omit reducing agents since the cyclic adduct with arginine is less reversible than the formation of a schiff base. In a parallel experiment, Fmoc-Lys-OH was subjected to the same kinetics assay with **PGO**, revealing no detectable adduct formation under standard conditions (Figure S1E). However, under modified conditions with freshly prepared sodium cyanoborohydride (30 mM) in the PBS buffer for reductive trapping, a median 12.3% modification of Fmoc-Lys-OH with **PGO** was detected at 15 min (Figure S1F). Conversely, no detectable modification of Fmoc-Lys-OH with **Nin** was observed under reducing conditions. It should be noted, however, that Hacker and co-workers have reported detectable modification of lysine with an alkyne-modified **PGO** (**PGO-Alk** ^17^) in cancer cell and *E. coli* lysates in absence of reducing agents.

To probe the extent of **Nin** labeling and its potential to react with proteinaceous arginines, we examined its modification stoichiometry on a model protein, bovine serum albumin (BSA), which contains 23 arginine residues. Following 30 min of **Nin** treatment, intact protein mass spectrometry revealed up to 6 labeling events under physiologic conditions (Figure 1D), with increased reactivity in a pH-dependent manner, as we observed in our free-arginine kinetics assay. The mass shift for each labeling event was consistent with engagement of **Nin** with a nucleophilic side chain forming dehydration products. Similar studies were also conducted for lysozyme, revealing a comparable pH-dependent reactivity profile modifying up to 11 residues on this protein containing 11 arginines^27^ (Figure S1G). While it was possible that engagement of other side chains could result in a similar mass shift, it was unlikely that all labeling events could be attributed to nonarginine side chains due to the reversibility of those reactions compared to arginine, especially considering that free lysine and cysteine are not detectably engaged by **Nin**. We additionally considered the potential for **Nin** to engage protein N-terminal amino groups, which exhibit higher nucleophilicity than lysine’s ε-amines due to their lower p*K*_a_. While transient N-terminal engagement may occur in aqueous solutions, the irreversible Ruhemann’s purple-type reaction requires higher temperatures or dehydrating conditions to occur. The reversible reaction releasing active **Nin** from the hemiaminal intermediate is therefore expected to predominate, leading to preferential engagement of arginines.

## Ninhydrin Probe Development

Encouraged by Fmoc-arginine engagement and robust, rapid labeling of two purified proteins, we pursued the development of a **Nin** chemical probe for testing in complex proteomes. We synthesized **Nin-Alk** and the reported arginine-reactive probe **PGO-Alk** ^17^ (Figure 2A). We visualized their relative reactivity across the proteome (Figure 2B). Mino cell lysates were treated with the indicated probe for 1 h. Following incubation, lysates were subjected to treatment with an azide-conjugated TAMRA fluorochrome under Cu­(I)-catalyzed Azide–Alkyne Cycloaddition (CuAAC)^28^ conditions. Methanol was added, and precipitated proteins were resuspended and separated by SDS-PAGE. In-gel fluorescence scanning revealed dose-dependent labeling for both probes across the proteome, with **Nin-Alk** exhibiting higher levels of labeling at all concentrations tested (Figure 2B). As a benchmark, we compared proteome-wide labeling by **Nin-Alk** and **PGO-Alk** to the pan-cysteine probe **IA-Alk**.^1^ Both **Nin-Alk** and **PGO-Alk** exhibited reduced labeling compared to **IA-Alk** (Figure 2B). Curious as to if a regioisomer of **Nin-Alk** would have similar labeling, we also synthesized **4-Nin-Alk** (Figure 2A), which exhibited similar labeling of cell lysates compared to **Nin-Alk** (Figure S2A).

**Figure 2:**
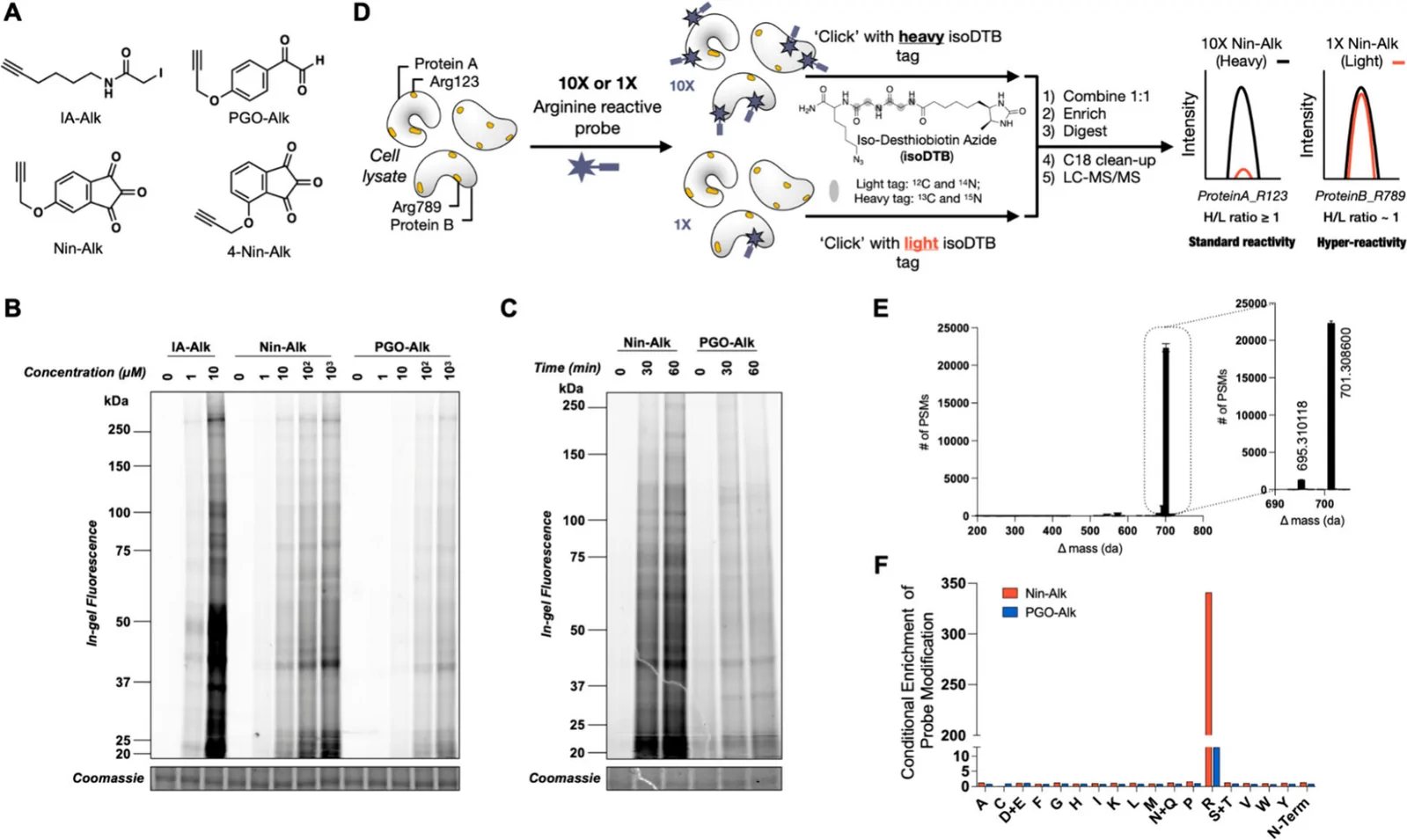
| Comparative profiling and biochemical characterization of arginine-reactive electrophiles. **A.** Chemical structures of electrophilic probes used in this study, including **IA-Alk** (cysteine-reactive control), phenylglyoxal alkyne (**PGO-Alk**), and two alkyne-functionalized ninhydrin derivatives, **4-Nin-Alk** and **Nin-Alk**. **B.** Qualitative, gel-based assessment of proteome-wide reactivity of alkyne-bearing covalent probes in Mino cell lysate. Soluble proteome (1.5 μg μL^–1^) was treated with each probe at the indicated concentration for 1 h, followed by CuAAC conjugation with TAMRA-N_3_, separation by SDS–PAGE, and in-gel fluorescence scanning. **C.** Qualitative time-dependent reactivity of **Nin-Alk**, and **PGO-Alk** in live cells. Mino cells were treated with 100 μM of the indicated probe for 0, 30, or 60 min, followed by lysis, CuAAC conjugation with TAMRA-N_3_, separation by SDS–PAGE, and in-gel fluorescence scanning. Uncropped gel images available in SI. **D.** Schematic of the Reactive Arginine Profiling (RAP) workflow to assess arginine reactivity. Cell lysates were treated with an arginine-reactive electrophile at high (1 mM) or low (100 μM) concentrations and subjected to copper-catalyzed azide–alkyne cycloaddition (“click” chemistry) with isotopically light or heavy isoDTB tags. Samples were combined 1:1, enriched, digested, and analyzed by LC-MS/MS. Peptides from hyper-reactive arginine residues show similar heavy/light (H/L) intensities, whereas less reactive sites are preferentially labeled at higher probe concentrations, yielding elevated H/L ratios. **E.** Open-mass search analysis of modified peptides reveals a predominant Δmass of 701.308600 and 695.310118 Da, corresponding to covalent modification of arginine by **Nin-Alk** and heavy or light isoDTB tag conjugation, respectively. Error bar represents SD. **F.** PSM-level conditional enrichment for probe-modified peptides by amino acid and protein N-terminus content. **Nin-Alk** shows strong and selective enrichment for arginine-containing peptides, whereas **PGO** exhibits weaker enrichment.

We next investigated whether **Nin-Alk** was capable of labeling live cells in a time-dependent manner. Mino cells were treated for 0, 30, or 60 min with the indicated probe (100 μM). Labeled cells were pelleted, lysed, and subjected to conjugation with TAMRA-azide as described above. In-gel fluorescence scanning of SDS-PAGE separated proteins revealed improved time-dependent labeling with **Nin-Alk** compared to **PGO-Alk** (Figures 2C **and** S2B). **4-Nin-Alk**, like **PGO-Alk**, exhibited reduced labeling in live cells compared to **Nin-Alk**, affirming our focus on **Nin-Alk** as a potential probe for characterizing arginine reactivity.

To evaluate **Nin-Alk**’s potential as a pan-arginine chemical probe, we developed a workflow utilizing the isotopically distinct azide-bearing desthiobiotin (isoDTB) tags developed by Hacker and co-workers^29^ (Figure 2D). Cell lysates were treated with **Nin-Alk** at either 100 μM or 1 mM for 1 h, and the resulting alkyne-labeled arginines were conjugated to light or heavy IsoDTB tags via CuAAC. Labeled proteome pairs were combined and processed by single-pot, solid-phase sample preparation (SP3), yielding enriched, desalted peptides for subsequent mass spectrometry analysis.^30^ We call this pipeline Reactive Arginine Profiling (RAP).

We first performed an open search to validate detection of intact probe modification. Open searches characterize the frequency of mass modifications to peptides, which allows for the identification of large mass differences between unmodified peptide sequences and experimentally observed precursors.^31^ For our **Nin-Alk**-treated lysates, we observed one major mass modification, + 701.308600 da, which corresponds to the **Nin-Alk** clicked to the heavy isoDTB tag without subsequent dehydration (Figure 2E). A less abundant mass corresponding to **Nin-Alk** clicked to the light isoDTB tag was also detected (+695.310118 da, Figure 2E and Table S1), which we expected given the 10-fold lower concentration of **Nin-Alk** used in the light channel.

Having established the dominant probe-derived mass shifts which implicated all nucleophilic residues, we incorporated these modifications into a closed-search workflow to enable confident assignment of probe-modified peptides. Although variable modifications were restricted to a subset of nucleophilic residues during database searching (Arg, Lys, Tyr, Trp, His, Cys), probe-modified peptides were subsequently identified based on their characteristic mass-shift at the PSM level, independent of site localization. Because the defining feature of probe-modified peptides is the mass shift itself rather than its assigned position, this approach captures modified peptides regardless of where the modification is localized during database searching. Using the predominant **Nin-Alk** adduct and the dominant reported **PGO-Alk** derived mass shift, we extracted probe-modified peptide-spectrum matches (PSMs) from RAP data sets and previously reported **PGO-Alk** data sets. To assess residue-level specificity in a proteomic context, conditional enrichment of probe-modified PSMs revealed strong preference for arginine-containing peptides for both **Nin-Alk** and **PGO-Alk**. Notably, **Nin-Alk** exhibited substantially higher enrichment for arginine-containing peptides (341.2) compared to other amino acids and N-termini, whereas **PGO-Alk** showed more modest engagement (13.0) (Figure 2F). These results support enhanced arginine-directed reactivity of **Nin-Alk**. Additionally, RAP samples were prepared in a nonborate buffer, which until now has been ubiquitous in arginine reactivity workflows using glyoxal probes.^14,17–19^

With data suggestive that **Nin-Alk** was an arginine-targeted probe, we were curious as to if our RAP workflow would enable identification of reactive arginines on model proteins BSA and lysozyme. We supplemented Mino cell lysates with purified BSA and lysozyme (1% w/w each) prior to probe treatment. Utilizing the chemical proteomics pipeline described, we identified 5 arginines on BSA and 8 arginines on lysozyme engaged by **Nin-Alk** (Figures S2C and S2D). Modified sites were detectable across the protein and consistent with the number of modifications detectable following treatment with **Nin** under physiologic pH in our intact protein studies (Figures 1D and S1G).

We next compared the performance of **Nin-Alk** and **PGO-Alk** in a comparative RAP experiment. Paired lysates were each treated with either probe (1 mM; 1 h) and conjugated to a heavy or light isoDTB tag. UpSet plot analysis revealed that the majority of **PGO-Alk**-engaged peptides were also engaged by **Nin-Alk** (n = 2862 of 2871 arginines are shared) while remarkably few arginines are uniquely engaged with **PGO-Alk** (n = 9) (Figure 3A, Table S2). This high overlap suggests that most **PGO-Alk**-reactive arginines are also accessible by **Nin-Alk**. Conversely, an additional 713 arginines are uniquely engaged by **Nin-Alk**, suggestive that the probe engages a broader subset of arginines in the proteome. Most arginines engaged by both probes were preferentially engaged by **Nin-Alk** (2704 arginines), whereas a significantly smaller fraction of sites were favored or exclusively engaged by **PGO-Alk** (n = 44) (Figure 3B).

**Figure 3:**
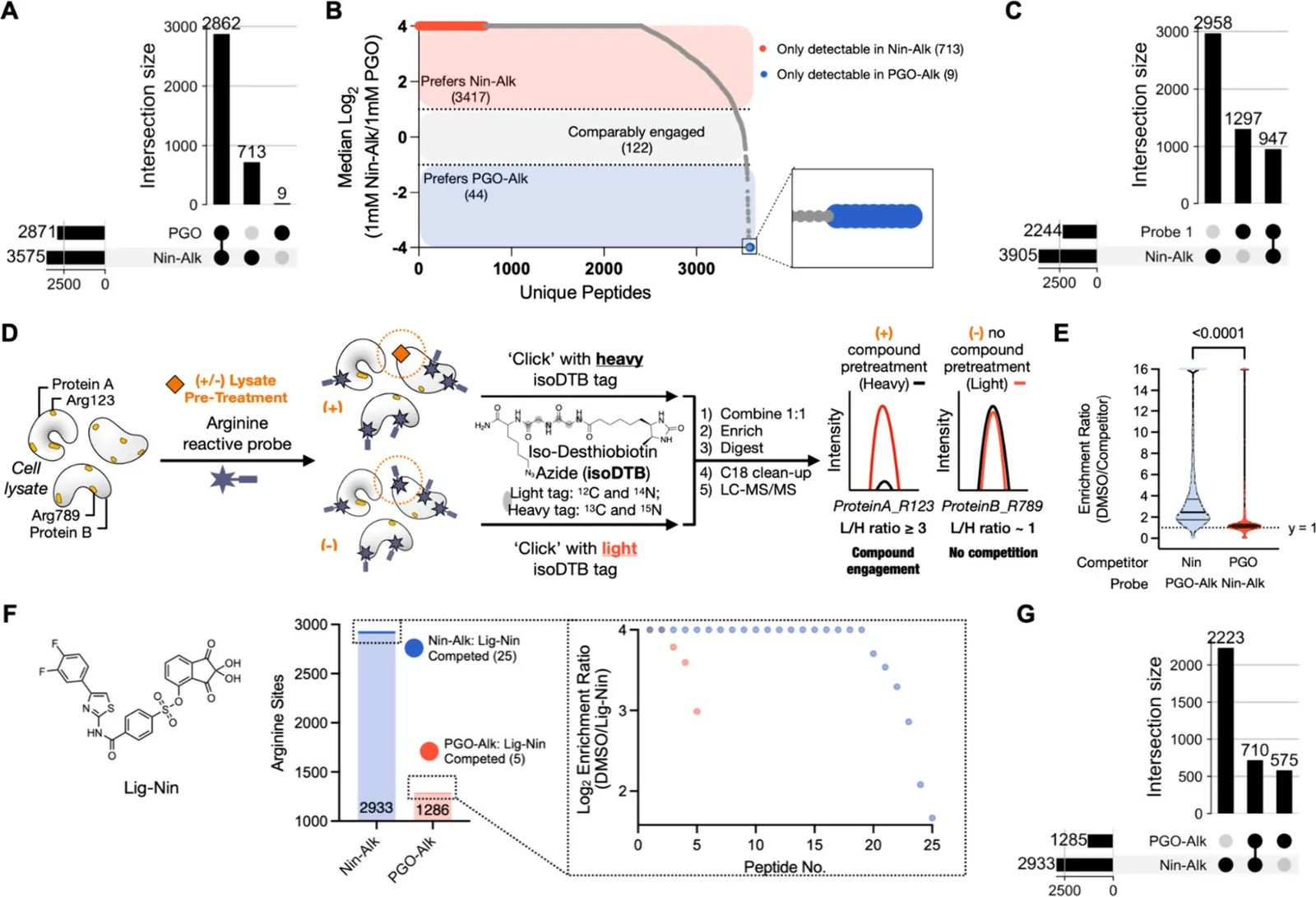
| Comparative profiling of PGO-Alk and Nin-Alk demonstrates increased saturation and promiscuity for Nin-Alk. **A.** UpSet plot demonstrating high overlap between **PGO-Alk**-engaged arginines and **Nin-Alk**-engaged arginines at 1 mM (1 h), with **Nin-Alk** engaging more targets. **B.** Waterfall plot showing median log_2_ ratios of 1 mM **Nin-Alk** to 1 mM **PGO-Alk** isoDTB enrichment (*n* = 6). Peptides with log_2_(**Nin-Alk**/**PGO-Alk**) ≥ 1 were classified as preferring **Nin-Alk**, whereas those with log_2_(**Nin-Alk**/**PGO-Alk**) ≤ −1 preferred **PGO-Alk**. Because **PGO-Alk** generates a distinct oxidation side product, quantification was performed by merging closed searches corresponding to oxidized and unoxidized species; intensities for both light channels (±ox) were summed for each peptide, and the heavy channel was averaged across searches to prevent double-counting. **C.** UpSet plot comparing unique arginine sites identified in A549 cells by Wang et al. using **Probe 1** and by **Nin-Alk** RAP profiling. **D.** Competitive RAP workflow. Cells or cell lysates are treated with an arginine-reactive electrophile or DMSO vehicle for 1 h prior to treatment with **Nin-Alk** (1 mM, 1 h). Labeled lysates are subjected to copper-catalyzed azide–alkyne cycloaddition (“click” chemistry) with isotopically light (DMSO treated) or heavy (competitor-treated) isoDTB tags. Samples are combined 1:1, enriched, digested, and analyzed by LC-MS/MS. Peptides containing arginine residues not engaged by competitor show similar heavy/light (H/L) intensities, whereas peptides containing arginines that are engaged by competitor yield at least a 3-fold decrease in H/L ratios across all replicates. **E.** Pretreatment of cell lysates with Ninhydrin (**Nin**) competes away sites of **PGO-Alk** labeling whereas pretreatment of cell lysates with **PGO** does not affect **Nin-Alk** labeling. All treatments were performed at 1 mM for 1 h. **F.** Structure of **Lig-Nin** and competitive profiling of reactive arginines using **Nin-Alk** and **PGO-Alk** in biological duplicates. **Lig-Nin** pretreatment of cell lysate (50 μM, 1 h) or DMSO prior to treatment with **Nin-Alk** (1 mM, 1 h) or **PGO-Alk** (1 mM, 1 h) in the competitive RAP workflow revealed expanded proteome-wide arginine coverage and a greater number of competed arginine sites using **Nin-Alk** relative to **PGO-Alk**, with 2933 quantified arginine sites and 25 competed sites identified by **Nin-Alk** compared to 1286 quantified sites and 5 competed sites identified by **PGO-Alk**. Insets highlight the subset of competed peptides and respective enrichment ratios corresponding to arginine sites engaged by **Lig-Nin**. **G.** UpSet plot comparison of arginine sites shared or uniquely engaged by **Nin-Alk** (*n* = 2223) and **PGO-Alk** (*n* = 575) with 710 sites shared between probes.

These findings suggest that **Nin-Alk** achieves broader and more uniform engagement of reactive arginines and is therefore well suited for proteome-wide screening applications analogous to **IA-Alk** in cysteine profiling. **Nin-Alk** also improves downstream analysis workflows, as it yields a single dominant mass adduct and clean isotopic labeling profiles. In contrast, arginine-probe adducts arising from both **PGO-Alk** and another glyoxal based probe, **Probe 1**, are reported to yield the expected mass and a second but comparably abundant rearranged moiety.^17,19^ We also observed this limitation with **PGO-Alk** (Table S3). The emergence of multiple mass products significantly complicates downstream quantitative analysis and can be observed on arginines and lysines. For our comparative studies we performed searches with both the nonoxidized modification and oxidized modifications (Figures S3A and S3B, Tables S2, S4, and S5). These data revealed comparable reactivity profiles for both adducts and combining both searches into our quantitative pipeline, while cumbersome, did not alter our results.

To directly compare arginines detectable by RAP with **Probe 1**, we performed RAP in A549 cells and compared data sets, which again revealed a substantial expansion in arginine coverage by **Nin-Alk** (Figure 3C, Table S6). While **Probe 1** detected 2244 arginines, **Nin-Alk** detected 3905 arginines, nearly 3000 of which were uniquely engaged. While both probes engaged a shared set of proteins (n = 589) (Figure S3C), **Nin-Alk** identified substantially more arginine sites within these shared targets, uniquely detecting 1,657 unique arginines compared to 793 identified by **Probe 1**, with 947 sites shared between probes (Figure S3D). These results further suggest that **Nin-Alk** has increased coverage of reactive arginines compared to glyoxal-based probes, which is critical for competitive screening workflows (Figure S3D).

With evidence that **PGO-Alk** was unable to saturate targets, we evaluated the ability of either warhead to compete away labeling by other probe. We employed our RAP workflow but pretreated cell lysates with **Nin**, **PGO** or DMSO vehicle (1 mM, 1 h) prior to incubation with **PGO-Alk** or **Nin-Alk** (1 mM, 1 h, Figure 3D, Tables S7 and S8), respectively. The median enrichment ratio of arginines engaged by **PGO-Alk** in the absence vs presence of **Nin** was 2.44, suggestive of significant competition by **Nin** (Figure 3E, Table S7). Conversely, the median enrichment ratio of arginines engaged by **Nin-Alk** in the absence vs presence of **PGO** was 1.16, indicating that **PGO** pretreatment has little effect on **Nin-Alk** labeling (Table S8).

These data suggests that **PGO-Alk** may be unable to saturate targets to the extent required for competitive screening approaches. To compare the ability of **Nin-Alk** and **PGO-Alk** to function as pan-reactive probes for competitive screening campaigns, we synthesized a ninhydrin-bearing ligand **Lig-Nin** (Figure 3F).^32^ Mino cell lysate was pretreated with **Lig-Nin** (50 μM, 1 h) or DMSO vehicle prior to treatment with **Nin-Alk** or **PGO-Alk** (1 mM, 1 h, Figure 3D). Using **Nin-Alk**, 2933 total arginine sites were identified, of which 25 were significantly competed by **Lig-Nin** (Figure 3F, Table S9), defined as peptides exhibiting greater than 3-fold reduced probe labeling following competitor pretreatment in both replicates. In contrast, **PGO-Alk** identified 1286 total arginine sites, with only 5 competed sites identified (Figure 3F, Table S10). Comparison of these data sets revealed that **Nin-Alk** identified 2223 unique arginine sites, whereas 710 sites were shared between probes and 575 were uniquely identified by **PGO-Alk** (Figure 3G). Comparison of proteins containing these reactive arginines revealed **Nin-Alk** uniquely identified 704 proteins, with 407 proteins shared between data sets and only 88 proteins uniquely identified by **PGO-Alk** (Figure S3E). These findings support the claim that **Nin-Alk** provides expanded coverage of reactive arginines in a competitive screening format.

## Chemical Proteomic Profiling of Arginine Labeling

Having validated the arginine-targeted pan-reactivity of **Nin-Alk**, we next leveraged the data to identify reactive proteinacious arginines in Mino cell lysates. Raw data collected from the RAP workflow comparing 1 mM and 100 μM **Nin-Alk** labeling conditions were subjected to a modified isoDTB closed-search workflow.^29^ We captured 10,642 modified peptide ions mapping to 6,888 unique arginines across 2,076 distinct proteins (Figure 4A and Table S11). Despite a recent observation from the Hacker group that **PGO-Alk** modified arginines are very rarely (0.2%) recognized by trypsin as a proteolytic cleavage site, we found that a greater subset (6.74%) of our **Nin-Alk** modified arginines were still recognized by trypsin for cleavage.^17^

**Figure 4:**
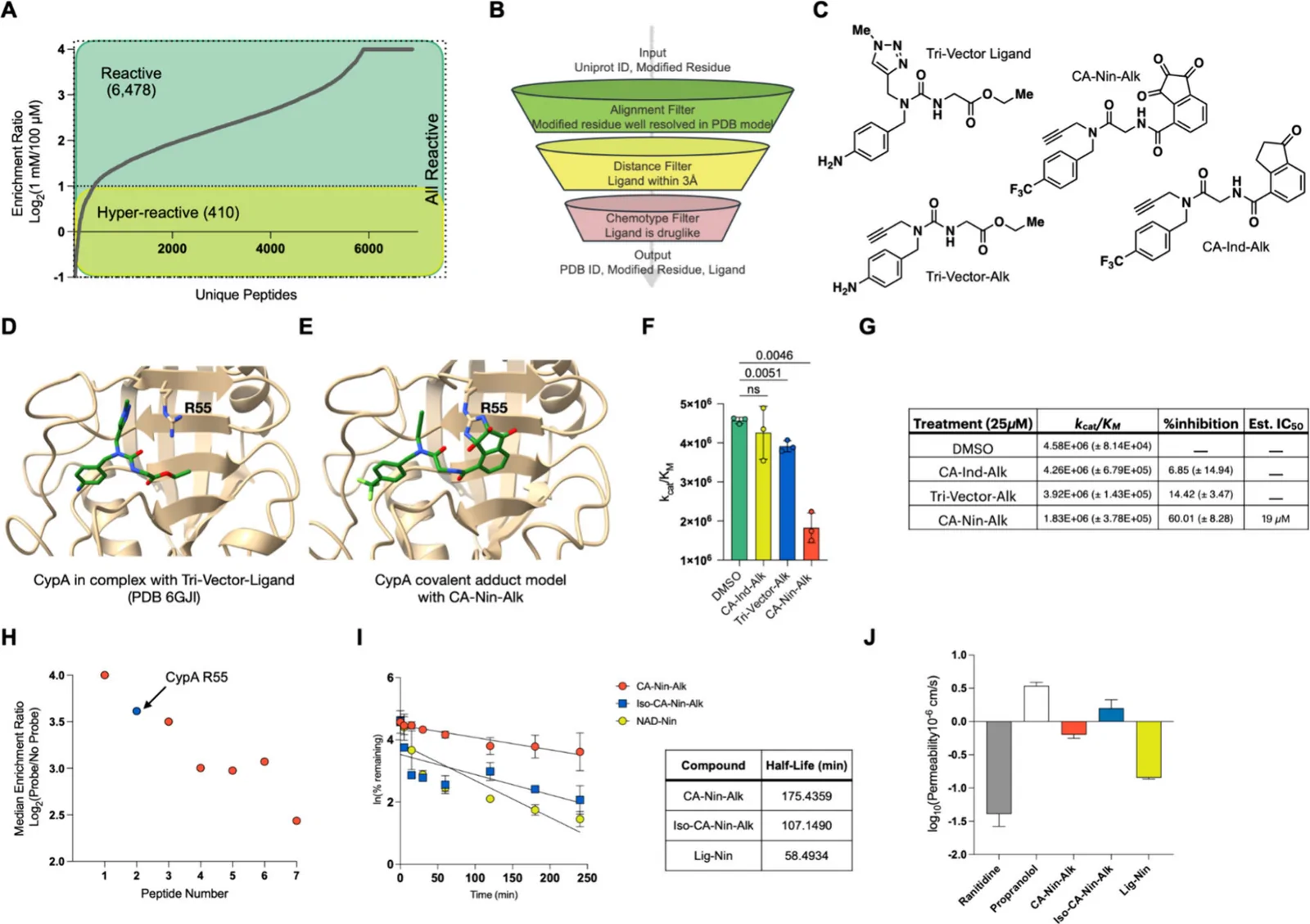
| A proof-of-concept ninhydrin-endowed probe targeting a reactive arginine. **A.** Waterfall plot showing the distribution of median RAP enrichment scores (log_2_ heavy-to-light [H/L] ratios) for all quantified arginines across 4 biological replicates. Arginine residues with log_2_(H/L) ≤ 1 were classified as hyper-reactive (*n* = 410), representing a subset of the total reactive arginines identified (*n* = 6,888). See Figure 2D for a schematic of the RAP workflow. **B.** Schematic of the in-house structure-based filtering pipeline used to identify candidate ligands in proximity to modified arginines. Input UniProt IDs and modified residue positions were filtered by PDB alignment, ligand proximity (≤3 Å), and drug-likeness of the ligand to prioritize hits for follow-up.**C.** Chemical structures of the trivector ligand and trivector alkyne, a clickable analog previously shown to bind cyclophilin A (CypA). The trivector alkyne contains an alkyne moiety that enables CuAAC-based enrichment and inspired the synthesis of **CA-Nin-Alk**. **D.** Published cocrystal structure of CypA bound to the trivector ligand (PDB: 6GJI),^33^ showing R55 positioned at the periphery of the ligand binding site. **E.** Covalent docking model of **CA-Nin-Alk** to R55 of CypA, showing similar binding orientation as the trivector ligand. **F.** CypA activity measured by a coupled peptidyl-prolyl isomerase assay. Preincubation of 1 μM recombinant CypA with **CA-Nin-Alk**, a previously reported **Tri-Vector-Alk** analog, and noncovalent, indanone-bearing **CA-Nin-Alk** analog **CA-Ind-Alk** (25 μM; 1 h) decrease CypA catalytic efficiency (k_cat_/K_m_) by 60.01 ± 8.3%, 14.42 ± 3.5%, and 6.85 ± 14.9% respectively compared to DMSO. Data represent mean ± s.d. (*n* = 3). **G.** Summary of kinetic parameters (k_cat_/K_M_), percent inhibition, and estimated IC_50_ values for indicated treatments. **H.** Chemoproteomic enrichment of peptides labeled by CA-Nin-Alk (10 μM, 1 h) in mino cell lysate. Each data point represents a distinct modified peptide; covalent labeling of R55 in cyclophilin A (CypA) is highlighted in blue. **I.** Human plasma stability of **CA-Nin-Alk**, **iso-CA-Nin-Alk**, and **Lig-Nin** at 10 μM assessed by LC-MS/MS over 240 min at 37 °C. Data are plotted as ln­(% remaining) versus time; half-lives were calculated from the slope of the linear regression as *t*_1/2_ = ln(2)/|slope|. Data represent mean ± s.d. (*n* = 2). **J.** Passive membrane permeability of **CA-Nin-Alk**, **iso-CA-Nin-Alk**, and **Lig-Nin** assessed by PAMPA. Ranitidine and propranolol were included as low and high permeability controls, respectively. Data represent mean ± SEM (*n* = 3).

The higher probe concentration range used in our RAP experiments may be less suitable for prolonged live-cell labeling conditions. Therefore, we evaluated **Nin-Alk** performance in both live cells and lysates with reduced concentrations (100 μM and 10 μM) (Figure S4A and S4B, respectively). Strikingly, our in-cell and lysate treatments at these concentrations resulted in less than 100 quantified arginines for each condition (Figures S4A and B). This reduced labeling is consistent with what Zanon and co-workers reported for PGO-Alk in cancer cell and *E. coli* lysates.^17^ Live cell data contained intracellular proteins of interest such as the Akt modulator TCL1A, mitochondrial GTPase EFTU, and the endoplasmic reticulum disulfide isomerase PDIA3, indicating that **Nin-Alk** is capable of entering cells. However, the low proteome coverage limits the application of this probe at such concentrations in live cells or lysates.

We speculate that the comparatively weak labeling of arginines by **Nin-Alk** and **PGO-Alk** at 100 μM and lower reflects several factors. Productive covalent engagement requires both of the probes to exist in their dehydrated, electrophilic form, which represents only a fraction of the total population in aqueous solution for engagement to occur. In addition, because the cellular proteome contains a vast number of arginine residues with measurable basal reactivity, lower probe concentrations are expected to preferentially enrich only the most highly abundant sites, while the majority of labeled arginines remain below the threshold required for robust enrichment and confident detection by LC-MS/MS. Other factors, including competing reactions with endogenous biomolecules, may further reduce the effective concentration of probe available for protein labeling. We therefore deprioritized lower-concentration treatments and focused our downstream analysis on the lysate RAP data set comparing 1 mM and 100 μM **Nin-Alk** engagement of nearly 7,000 arginines with the intention of utilizing **Nin-Alk** in a manner similar to that of **IA-Alk**.^1^

## Development of a Pipeline for Ligand-Directed Arginine Modification

To systematically identify which reactive arginines in our RAP dataset were amenable to targeting with ligands bearing arginine-targeted warheads, we developed an in-house computational structural proteomics pipeline (Figure 4B). The workflow compiles a list of UniProt IDs^34^ alongside their respective modified arginine residues and cross-references them with available high-resolution structures in the Protein Data Bank (PDB).^35^ The analysis parsed through 9,348 unique PDB files, identifying 33,402 occurrences of arginines from our data set. For each structurally resolved arginine, the pipeline identifies nearby ligands within 3 Å of the guanidinium group (Table S12). From these occurrences, 292 arginines were found to engage in 884 unique contacts with 461 distinct ligands. To prioritize functionally relevant ligands, we excluded primary human metabolites, crystallographic ions, and buffer components.

Applying this strategy, we identified R55 in cyclophilin A (CypA) as a reactive arginine proximal to recently reported trivector noncovalent binders^33^ that provide synthetically tractable starting points (Figures 4C and 4D). While these molecules have been previously reported to noncovalently engage CypA, they have not been evaluated for their inhibition of CypA activity. Structure-guided ligand optimization led to the development of **CA-Nin-Alk** (Figure 4C), where the ethyl ester was replaced with a ninhydrin warhead, the urea was substituted with an amide, and the aniline NH_2_ was replaced with an isosteric trifluoromethyl group. This substitution leveraged the trifluoromethyl group’s ability to electronically and spatially mimic an aniline while avoiding potential liabilities of a free amine noted in the original trivector ligand report, thereby maintaining the binding geometry. This design positioned the ninhydrin warhead appropriately for covalent engagement with R55 in our docking models to the crystal structure of cyclophilin A in complex with a trivector ligand (PDB 6GJI)^33^ (Figure 4E). To control for covalent reactivity, we synthesized **CA-Ind-Alk**, an indanone containing analog of **CA-Nin-Alk** that preserves the parent scaffold but lacks the ninhydrin electrophile (Figure 4C).

An *in vitro* washout-like activity assay revealed that **CA-Nin-Alk** attenuated CypA catalytic activity (Figures 4F, 4G and S5A, Table S13).^36^ Using a modified coupled peptidyl-prolyl isomerase (PPIase) assay with the chromogenic substrate suc-AAPF-pNA, recombinant CypA (1 μM) was preincubated with inhibitors **CA-Nin-Alk**, **CA-Ind-Alk**, **Tri-Vector-Alk** (25 μM; 1 h), or vehicle and diluted 100-fold into the reaction mixture for a final CypA concentration of 100 nM immediately before initiating catalysis. This dilution step should dissociate noncovalent complexes^37^ thereby mimicking washout conditions, thus indicating whether the interaction was covalent or noncovalent. Under these conditions, **CA-Nin-Alk** treatment reduced (k_cat_/K_m_) by 60.01 ± 8.28% relative to DMSO. The corresponding noncovalent analogs exhibited substantially weaker inhibition under identical conditions; **CA-Ind-Alk** showed minimal inhibition (6.85 ± 14.94% at 25 μM), and **Tri-Vector-Alk** reduced catalytic efficiency by only 14.42 ± 3.47% at 25 μM (Figures 4F and 4G). **CA-Nin-Alk** was additionally evaluated at 10 μM (33.21 ± 9.60%) (Figure S5B) and, based on a two-point interpolation, we estimate an apparent IC_50_ of ∼ 19 μM. These findings suggested that **CA-Nin-Alk** is either capable of covalently inhibiting CypA enzymatic activity or functions as a noncovalent inhibitor at 100 nM.

To assess the proteome-wide selectivity of **CA-Nin-Alk** and validate covalency for CypA at R55, we performed a probe-versus-no-probe chemoproteomic experiment at two concentrations in mino cell lysate. At 100 μM, **CA-Nin-Alk** enriched 287 modified peptides (Table S14). At 10 μM, only 7 modified peptides, including R55, were detected, demonstrating a marked reduction in off-target engagement at lower concentrations (Figure 4H and Table S15). The ligand scaffold likely plays a key role in directing this selectivity by engaging the binding pocket containing R55 and restricting the modification of other reactive arginines throughout the proteome. Together with the reduction in CypA catalytic efficiency observed in the PPIase assay, these results suggest that **CA-Nin-Alk** covalently inhibits CypA through its active-site arginine *in vitro*. A comparative RAP analysis at 1 mM between **CA-Nin-Alk** and its regioisomer **iso-CA-Nin-Alk** (Figure S5C), which repositions the ninhydrin warhead away relative to R55, revealed both a significantly reduced overall reactivity for **iso-CA-Nin-Alk** at CypA R55 and a median log_2_ selectivity between the probes ratio of 2.49 in favor of **CA-Nin-Alk**, likely driven by a combination of improved interactions with the binding pocket and better electrophile placement for improved engagement­(Figure S5E and Table S16).

We also evaluated CA-Nin-Alk engagement in live Mino cells. Cells were treated with **CA-Nin-Alk** at 10 μM, 100 μM, or 1 mM for 1 h. However, no **CA-Nin-Alk** modified peptides were detected at any concentration. This result could reflect issues both dependent and independent of the ninhydrin warhead, including but not limited to insufficient intracellular exposure, compound sequestration by efflux, or insufficient formation or recovery of the adduct. We nonetheless sought to determine whether ninhydrin-bearing molecules possess properties necessary for in cell labeling.

For covalent electrophiles, plasma stability provides a useful measure of effective reactivity under biologically relevant conditions and susceptibility to quenching by plasma proteins and endogenous nucleophiles. Human plasma stability was assessed by LC-MS/MS over 240 min and half-lives were calculated by fitting the decay in the parent compound over time to a first-order exponential model (Figure 4I). **CA-Nin-Alk** demonstrated the greatest stability (*t*_1/2_ = 175 min), followed by **iso-CA-Nin-Alk** (*t*_1/2_ = 107 min) and **Lig-Nin** (*t*_1/2_ = 58 min). These values fall within the range reported for clinically validated covalent warheads, including probe analog of Ibrutinib, which displays plasma half-lives of approximately 168 min.^38^ The improved stability of **CA-Nin-Alk** relative to **iso-CA-Nin-Alk** and **Lig-Nin** further suggests that the surrounding scaffold can modulate effective warhead reactivity.

We next assessed passive membrane permeability using the parallel artificial membrane permeability assay (PAMPA), with ranitidine and propranolol serving as low and high permeability controls, respectively. **iso-CA-Nin-Alk** demonstrated permeability comparable to propranolol, **CA-Nin-Alk** showed intermediate permeability, and **Lig-Nin** exhibited the lowest passive permeability, approaching that of ranitidine (Figure 4J). These findings demonstrate that membrane permeability is tunable through modification of the nonwarhead portions of the scaffold. Importantly, PAMPA measures only passive transcellular diffusion and cannot account for active uptake or efflux mechanisms. These data establish that ninhydrin-based arginine-directed covalent scaffolds can achieve a favorable balance of plasma stability and membrane permeability, and that the lack of covalent labeling by **CA-Nin-Alk** in live cell studies does not preclude further optimization or elaboration of the ninhydrin moiety toward intracellular activity.

## Characterization of Proteins Harboring Reactive Arginines

Having validated **Nin-Alk** as a pan-reactive arginine probe and evaluated ninhydrin-bearing small molecules as inhibitors, we were curious to more broadly explore the reactive arginome. From Mino cells, we identified 2076 proteins harboring 6888 reactive arginines (Figure 5A). Statistical overrepresentation analysis of proteins containing reactive arginines revealed enrichment for protein classes associated with protein translation, mRNA processing, and nucleic acid binding, among others (Figure 5B).^39,40^

**Figure 5:**
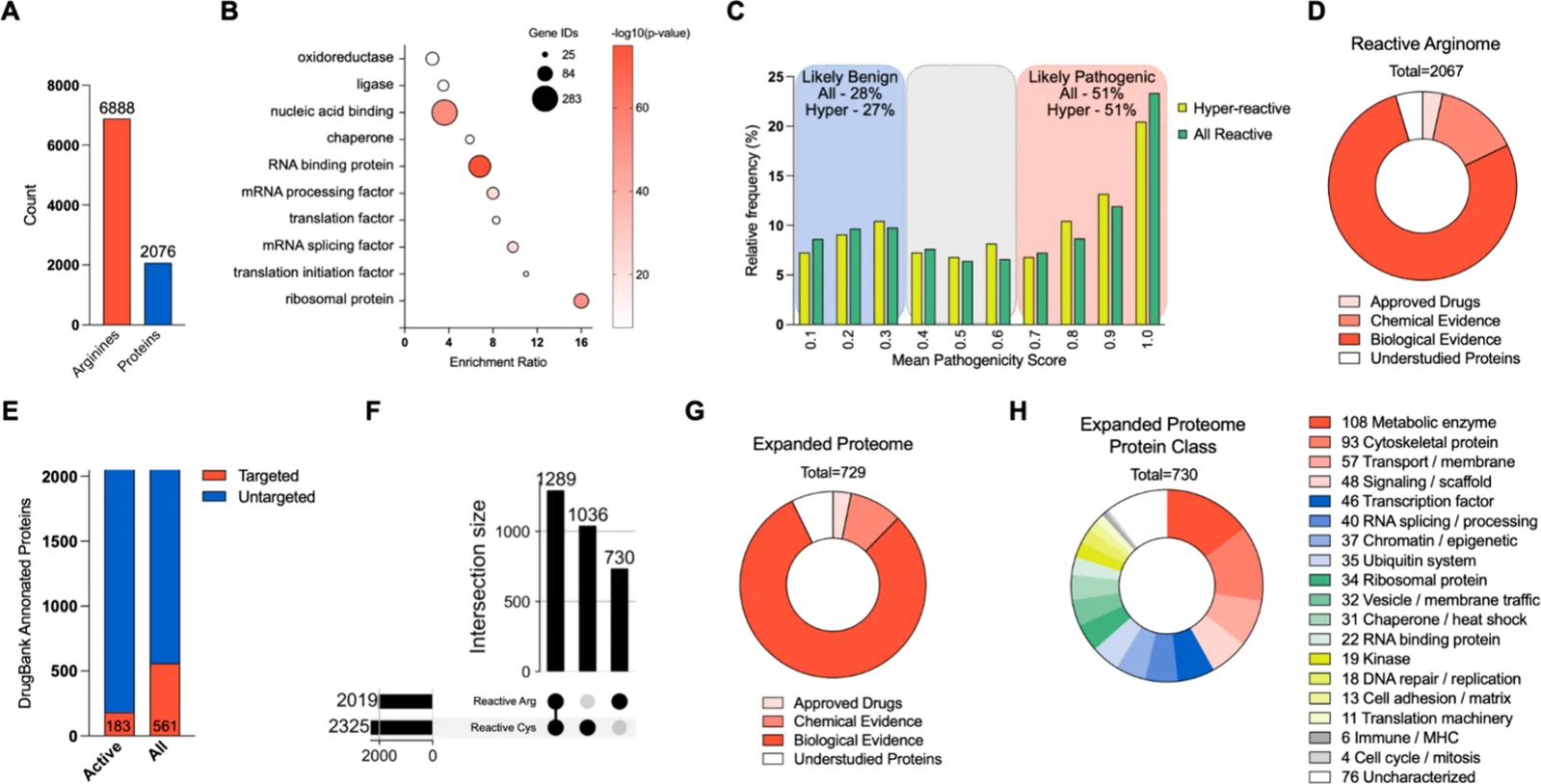
| Expanding the ligandable proteome through arginine. **A.** Total number of unique arginines and corresponding proteins identified using the RAP workflow (see Figure 2D and Table S11). **B.** Bubble plot of the top 10 most significantly enriched PANTHER protein classes. Statistical significance was calculated using Fisher’s Exact test and adjusted using the Benjamini–Hochberg procedure to control the false discovery rate at α = 0.05; only terms with adjusted p-value <10^–9^ are shown. Bubble size reflects the number of proteins in each class. **C.** Mean pathogenicity score of mutations to each hyper-reactive (*n* = 220) and reactive arginine (*n* = 4865), predicted using AlphaMissense­(Table S17).^41^ **D.** Target development level of proteins containing reactive arginines by Pharos, based on Target Central Resource Database classification, where Tclin = Approved Drug, Tchem = Chemical Evidence, Tbio = Biological Evidence, Tdark = Understudied Proteins. **E.** Number of proteins detected in RAP data set annotated as targeted in the DrugBank, either actively by approved pharmaceuticals (Active) or by any drug in the Drugbank including approved, investigational, experimental, withdrawn or illicit (All) **F.** Intersection of proteins harboring reactive cysteines and/or arginines as determined by IA-Alk engagement or **Nin-Alk** engagement and MS detection. **G.** Target development level of proteins containing reactive arginines but not reactive cysteines by Pharos, based on Target Central Resource Database classification, where Tclin = Approved Drug, Tchem = Chemical Evidence, Tbio = Biological Evidence, Tdark = Understudied Proteins. **H.** Functional protein class distribution of 730 proteins containing reactive arginines but not reactive cysteines in Mino cell lysates. Proteins were classified into 18 functional categories based on UniProt functional annotations; 76 proteins could not be assigned to a defined class­(uncharacterized).

We explored functional constraints by mapping mean pathogenicity scores^41^ (AlphaMissense) onto each reactive arginine (Figure 5C and Table S17). A high pathogenicity score indicates that mutations of a given residue are associated with significant functional impairment or disease-related phenotypes. Both hyper-reactive and all reactive residues are enriched at the high end of the pathogenicity scale, with 51% of enriched arginines falling into the likely pathogenic range, indicating a labeling bias toward arginines that are mutationally constrained, likely reflecting their critical structural or functional roles in maintaining cellular integrity. Since these arginines play critical roles in cellular function, they are less likely to undergo genetic mutations that could evade covalent engagement. Thus, covalent engagement at these essential positions likely mimics genetic perturbation, directly impacting residue functionality and consequently cellular processes, making them attractive and robust targets for drug and tool compound development.

We cross-referenced proteins harboring reactive arginines with Pharos,^42^ a database developed by the NIH to categorize a protein’s status as a drug target based upon available chemical and biological evidence. This analysis revealed that the majority of proteins harboring reactive arginines are potential therapeutic targets supported by strong biological evidence but currently lack chemical tools for modulation (Figure 5D). Complementary annotations from DrugBank^43^ further suggest an opportunity to expand covalent small-molecule engagement to a broader set of potentially druggable proteins through reactive arginine engagement (Figure 5E).

## Expanding the Covalently Targetable Proteome

To directly determine the potential of targeting reactive arginines compared to reactive cysteines, we compared our RAP data set to a paired cysteine reactivity profiling experiment also in Mino cells. Use of RAP revealed 730 new protein targets that do not harbor a cysteine that engages IA-Alk (Figure 5F, Table S18). These proteins account for 36% of the RAP data set, which is comparable to the 46% of proteins engaged by IA-Alk that are not detected to harbor reactive arginines. When we cross-referenced the 730 proteins containing reactive arginines and not reactive cysteines with Pharos, we again saw a striking enrichment for drug targets that have biological evidence for therapeutic value with currently no chemical matter enabling targeting of these proteins (Figure 5G, Table S18). Protein class assignments by PANTHER revealed that proteins harboring reactive arginines but not reactive cysteines include but are not limited to signaling proteins, transcription factors, and RNA binding proteins, further highlighting the value of expanding covalency to arginine (Figure 5H).

## Characterization of Reactive Arginines

Inspired by the unique roles arginines play in protein structure and scaffolding, we sought to characterize the broader structural and functional context of **Nin-Alk** engaged residues. Through secondary-structure mapping across the PDB, we found reactive arginines enriched in helices and flexible loops compared to β-sheets (Figure S6A and Table S19). To gauge local flexibility, we used IUPred2A, an energy-based predictor of intrinsic disorder, to compute per-residue scores from 0 (ordered) to 1 (disordered). Reactive arginines peaked in the moderate-disorder regime (0.3–0.4) yet ∼ 40% reside in well-ordered contexts (scores <0.2), demonstrating that both structural support and some degree of flexibility facilitate efficient covalent engagement (Figure S6B).

Beyond these structural contexts, we turned to cation-π contacts with aromatic side chains, quantifying interactions across all available PDB models for each protein. We applied two distance thresholds, 8 Å, which is the commonly used cutoff, and a more stringent 5.5-Å cutoff for true energetic relevance, measured from the arginine CZ atom to the aromatic ring-centroid. To ensure only geometrically plausible face-on and edge-on interactions were included, we added angular filters: for parallel (face-on) interactions, the CZ-to-centroid vector was required to lie within 30° of the ring normal, while for T-shaped (edge-on) interactions, deviation from 90° had to be ≤ 20°. Using these criteria, we identified 905 total cation-π contacts at 8.0 Å, while only 332 satisfied the stricter 5.5 Å threshold (Figure S6C and Table S20). Phenylalanine and tyrosine accounted for the majority of these contacts, compared to histidine and tryptophan. At the more stringent distance threshold, the distribution of aromatic partners mirrors the natural abundance of aromatic residues in the human proteome (Table S20). Unlike recent efforts to selectively label tryptophans that showed enrichment for tryptophans engaged in cation-π interactions,^44^ we do not see an enrichment of arginines engaged in cation-π interactions (Figure S6D and Table S20). This indicates that arginine reactivity is not biased by a specific type of π-donor or the stabilizing effect of a cation−π interaction.

We next measured salt bridges as the minimum distance between any arginine guanidinium nitrogen and Asp/Glu carboxylate oxygens for both PDB entries and AlphaFold^45^ models. In AlphaFold structures, these distances cluster tightly around ∼2.6 to 2.8 Å, whereas PDB entries span a broader 2.5 to 4.0 Å range with a median closer to 3.2 Å (Figures S6E, Tables S20 and S21). Likewise, relative solvent accessibility values derived from solvent accessible surface area (SASA)^46^ analysis on PDB models are systematically higher and more variable than those from AlphaFold (median RSA for AlphaFold predicted models = 0.19, median RSA for PDB experimental models = 0.33; Figure S6F, Tables S22 and S23). Although AlphaFold models offer near proteome-wide coverage, their monomeric, in-silico nature often yields overly “idealized” geometries that may not reflect the full range of biologically relevant conformations.^47–49^ This was exemplified in our secondary structure analysis on AlphaFold models, which showed a greater enrichment of alpha helices than determined in the PDB models (Figures S6A and G, Tables S19 and S24), which is a common feature of AlphaFold structures.^50^ To address this, we prioritized experimentally determined PDB structures, trading breadth for depth by focusing on a smaller set of proteins with multiple crystallographic or complexed snapshots that better approximate physiological contexts. In both cases, the experimental data capture greater conformational diversity and exposure compared to the predicted models, which we believe are critical parameters for accurately mapping covalent-probe hotspots.

We sought to dissect the biophysical and functional determinants that govern differential arginine reactivity using data sets of reactive and hyper-reactive arginines (Figure 4A). We defined hyper-reactive arginines as those which display similar enrichment at high and low concentrations (comparative ratio of intensity between conditions of less than 2), whereas less reactive arginines display concentration-dependent labeling (comparative ratio of at least 2). The median log_2_ ratio of enrichment between 1 mM and 100 μM **Nin-Alk** across all arginines was 2.50, reflecting dose-dependent labeling of most reactive arginines. Notably, 410 sites (∼6.0% of detected arginines) were hyper-reactive, fully engaged at 100 μM, while the remaining 6478 sites exhibited clear, concentration-dependent labeling. This percentage of arginines annotated as hyper-reactive is consistent with reports for cysteine and arginine.^1,2,4^ Moreover, proteins typically harbor a single hyper-reactive arginine but can contain multiple concentration-dependent reactive arginines (Figure S7A). pLOGO analysis of peptides with a modified arginine found no significant 2D sequence determinants of labeling, with some minor enrichment for acidic residues 3–4 residues away (Figure S7B).^51^

With our lists of either all reactive (any arginine engaging ninhydrin) or hyper-reactive (enrichment ratio less than 2) arginines, we set out to identify distinguishing features between the two sets. Even though arginine remains protonated under physiological conditions, cysteine and lysine reactivity have been linked to residue p*K*_a_ values,^52,53^ so we first asked whether arginine labeling follows a similar trend. Predicted p*K*_a_ values for all reactive arginines within the PDB were, as expected, tightly clustered around 12.5. Distributions for hyper-reactive versus all reactive arginine sites showed no significant differences (Figure S7C and Table S22), indicating that, unlike lysine (Figure S7D), intrinsic protonation state does not drive labeling efficiency. This finding was consistent for predicted AlphaFold structures (Figure S7D, Table S25).

Given the established importance of solvent accessibility for cysteine modification by iodoacetamide,^54^ we next assessed the relative solvent exposure of reactive arginines. SASA analysis^46^ revealed comparable relative solvent exposure distributions for hyper-reactive and all reactive sites, with both populations exhibiting partial rather than full surface exposure (median RSA for hyper-reactive = 0.31, median RSA for all reactive = 0.33, mean RSA for both = 0.33; Figure S7E and Table S22). Together with the absence of meaningful differences in the predicted p*K*_a_ values, these data indicate that intrinsic physicochemical properties alone are poor predictors of differential arginine reactivity. This finding has important implications for covalent ligand design. Whereas many successful cysteine-directed covalent inhibitors exploit unusually nucleophilic or highly accessible cysteines, our data suggest that differential arginine engagement is less dependent on privileged intrinsic residue reactivity. Instead, arginine-directed probes appear capable of engaging arginines across diverse structural and environmental contexts, with selectivity arising primarily from noncovalent interactions between the ligand scaffold and the binding site that positions the electrophile in proximity to a suitably oriented arginine residue.

To examine whether salt-bridge geometry and active site distance contributes to hyper-reactivity, we compared the salt bridge and active-site distances for hyper-reactive and all reactive arginines in PDB models (Figures S7F and S7G, Tables S20 and S22). Both populations exhibit broad, overlapping distributions (median distances = 3.2 Å for both hyper-reactive and all reactive sites), indicating that salt-bridge proximity alone does not distinguish the hyper-reactive subset.

## Discussion

Covalent probes have transformed chemical biology and drug discovery, but broadly expanding covalent strategies to amino acids beyond cysteine and lysine has been challenging. Our study establishes ninhydrin-based probes as selective and robust tools for covalent arginine labeling and reactivity profiling, significantly expanding the chemical proteomics toolkit and enabling new approaches to study arginine-mediated processes. Our ninhydrin-derived probes leverage the equilibrium between the hydrate and reactive dehydrate states of vicinal dicarbonyls, optimizing probe reactivity while minimizing nonspecific interactions and positions arginine as a promising target for broadly applicable covalent strategies. Recently reported glyoxal-based chemoproteomic approaches required 4 h of probe treatment in a borate buffer to enhance glyoxal-arginine adduct stability, achieving coverage of 4606 arginine sites across 4 cell lines.^19^ Our RAP workflow operates under standard PBS conditions while achieving coverage of nearly 7,000 sites in one cell line. Nevertheless, glyoxal warheads remain valuable in contexts where selective or attenuated reactivity is advantageous. **PGO**’s greater rotational freedom and lower intrinsic electrophilicity suggest that it could function analogously to chloroacetamides in cysteine-directed ABPP, providing a less globally reactive warhead. Future efforts might leverage this tunability in designing arginine-targeting covalent inhibitors with site-specific, tailored electrophilicity.

Unlike cysteine and lysine, whose reactivities heavily depend on intrinsic residue p*K*_a_ and high solvent accessibility, arginine residues demonstrate consistent reactivity independent of p*K*_a_ variations or extensive solvent exposure. This independence could be advantageous or disadvantageous for probe development: it allows for covalent engagement in structurally diverse and otherwise challenging environments, but privileged intrinsic reactivity is minimal for arginines compared to cysteine-directed covalent ligands. Moreover, while ninhydrin exhibits rapid intrinsic reaction kinetics toward arginine in simplified aqueous systems, the limited proteomic coverage of the minimal probe **Nin-Alk** at 10 to 100 μM in both live cells and lysates highlight that intrinsic electrophile reactivity alone is insufficient to predict performance in complex biological systems, where effective target engagement is additionally governed by probe concentration, stability, and competing reactions with other biomolecules.

Cell lysate labeling studies revealed that modified arginines are often partially buried rather than fully solvent-exposed, and our analyses of p*K*_a_ and solvent accessibility showed no meaningful differences between hyper-reactive and all reactive arginines, emphasizing that determinants of arginine reactivity remain poorly defined (Figures 1C, S6F, S7E). This behavior contrasts with the conventional paradigm in covalent ligand discovery where selectivity can be achieved by exploiting a limited subset of unusually nucleophilic or highly accessible residues. Our findings suggest that, for arginine-directed covalent ligands, selectivity may instead arise predominantly from noncovalent molecular recognition, with the ligand scaffold positioning the electrophile adjacent to a suitable oriented arginine residue. In this model, the covalent bond serves to reinforce a productive binding event rather than to capitalize on exceptional intrinsic reactivity of a targeted side chain. Consistent with this model, **CA-Nin-Alk** selectively engaged the reactive, but not hyper-reactive, residue R55 on cyclophilin A in cell lysates, while the regioisomeric probe **iso-CA-Nin-Alk** showed diminished targeting when the ninhydrin warhead was misaligned relative to R55 (Figure 4E, Figure S5E). Together, these findings suggest that precise scaffold recognition and electrophile orientation may be key determinants of selectivity for arginine-directed covalent ligands.

Arginines are frequently localized at protein–protein interaction (PPI) sites, where their covalent modification is poised to directly modulate or disrupt critical interaction surfaces. Both cation-π interactions and salt bridges are more prevalent in proteins from thermophilic organisms, enhancing protein stability. We observe that reactive arginines frequently engage in these stabilizing interactions, although these features were not enriched in hyper-reactive arginines compared to generally reactive ones. This trend is consistent with the absence of significant differences in predicted p*K*_a_ or solvent accessibility between the two groups. In our RAP data set, we identify 290 unique arginines stabilizing PPIs within the PDB, forming 519 unique PPI pairs. Among these, 145 arginines participated in 193 cation-π interactions, and 212 arginines were involved in 326 unique salt bridge pairs (Table S20). Despite the prevalence of these specific interactions, our analysis found that fully solvated arginines are underrepresented based on their physicochemical properties. This rarity may partly reflect the inherent dynamic nature of fully solvated arginines, as their positions might only be reliably resolved when stabilized at interaction interfaces, resulting in relatively lower RSA values being captured in our bioinformatic analyses. Importantly, these interactions provide valuable insights into structural contexts that could guide rational optimization of arginine-targeted covalent probes.

Integration of experimental PDB structures significantly enhanced our structural analyses compared to predictions based on computational AlphaFold models. Although AlphaFold offers extensive near-proteome-wide coverage, these models frequently depict idealized conformations, lacking critical contextual interactions present in biological environments. By contrast, the use of PDB structures, though computationally more intensive, owing to the need for extensive sequence alignments, the handling of large cryo-EM complexes, and the presence of multiple structural entries for individual proteins, provided more realistic and biologically representative data. Specifically, experimental structures captured greater diversity in salt bridge geometries, solvent accessibility, and interaction distances (Figures S6E and S6F), all crucial for accurately predicting arginine reactivity. This approach underscores the value of incorporating experimentally determined structures when studying residue-level reactivity, particularly for interpreting complex biophysical features that may be underrepresented in predicted models.

Recent work studying iodoacetamide-cysteine reactivity found that simple features such as 2D sequence, p*K*_a_ or even data derived from AlphaFold models were not sufficient to predict a cysteine’s relative reactivity.^55^ More accurate prediction required experimentally determined structures to start to model residue-level reactivity. While AlphaFold offers transformative accessibility and continues to enable exciting applications such as predictive covalent docking, the current generation of models often struggles with accurate representation of side chain packing and interaction geometries. As we expand our platform to profile reactive arginines across more cell types, the growing bulk of arginine reactivity data may make AlphaFold-derived structures increasingly useful at scale, particularly given the computational demands of mining the PDB. Future iterations of Alphafold, which are showing improvements in side chain accuracy, may further enhance its utility for predicting covalent probe engagement.

Ninhydrin has been reported to inhibit the catalytic activity of mitochondrial aconitase at doses comparable to those used in our assays,^56^ but the mechanism of this inhibition has not been elucidated. Dysregulation of its activity has been implicated in metabolic disorders, neurodegeneration, and tumor progression.^57^ Mitochondrial aconitase has garnered interest as a metabolic vulnerability in cancer and as a redox-sensitive node in neurodegenerative diseases, with small molecules and metabolic poisons such as fluoroacetate demonstrating proof-of-concept for pharmacological modulation.^58^ Our **Nin-Alk** hyper-reactivity RAP data set revealed four modified arginines on mitochondrial aconitase (R479, R474, R564 and R607) which are all located within or proximal to the enzyme’s active site. Among these, R479 was consistently detected across all samples and occupies a central position in substrate coordination (Table S11, Figure S8A, PDB 1B0J).^59^ Covalent docking simulations further demonstrated that ninhydrin modification at this site would sterically occlude the active site, likely preventing substrate binding (Figure S8B). These data suggest that arginine-targeting covalent inhibitors provide an avenue to modulate mitochondrial aconitase function. Future work will focus on functional validation of ninhydrin-mediated aconitase inhibition.

This study establishes a robust framework for selective covalent arginine modification using ninhydrin-derived probes. We introduce RAP as a chemical proteomic approach to classify arginine reactivity, facilitating rational probe development and the identification of key arginines for therapeutic targeting. Future research will focus on elucidating additional structural determinants of arginine reactivity, expanding the application of ninhydrin-based probes to a broader range of therapeutically relevant targets, and leveraging the RAP platform as a competitive screening tool for the discovery of arginine-reactive small molecules. Importantly, the ability to perform competitive screening without requiring enrichment handles will simplify the evaluation of candidate molecules, enhancing throughput and reducing synthetic complexity, thus accelerating the development of novel arginine-targeted therapeutics. By advancing strategies for arginine-selective covalent modification, this work paves the way for novel chemical biology tools and next-generation covalent therapeutics.

## Supplementary Material





## Notes

Additional figures depicting evaluation of ninhydrin as a covalent modifier of lysine and free thiols, figures comparing **Nin-Alk** to covalent probes engaging cysteine and arginine, figures visualizing live-cell RAP profiling in Mino cells, figures depicting exploration of **CA-Nin-Alk** and its regioisomer, figures depicting biophysical and functional features of reactive arginine residues, figures supporting covalent inhibition of aconitase by ninhydrin at R479 *in vitro*, mass spectrometry data tables, data tables related to analysis of reactive arginine features, methods, uncropped gels, representative compound traces and, synthesis of probes and molecular characterization (PDF)
Mass spectrometry data tables for open and closed searches, peptide-level quantification of arginine labeling, competition and dose-response experiments, Cyclophilin A activity assay data, and data tables related to analysis of reactive arginine features, including pathogenicity predictions, secondary structure, intermolecular interactions, solvent accessibility, predicted pKa, and ligand proximity (XLSX)

## Notes

Safety Statement: No unexpected or unusually high safety hazards were encountered in the course of this work. SeO_2_ is an acute toxic oxidant and should be handled with appropriate PPE in a well-ventilated fume hood. Selenium-containing waste was collected and disposed of with heavy metal waste.
