# Supplementary material for: Ninhydrin as a Covalent Warhead for Chemical Proteomic-Enabled Discovery and Selective Engagement of Reactive Arginines
Source: ACS Cent Sci. 2026 Sep 30;12(10):1610–24. doi: 10.1021/acscentsci.6c00922 (PMC13720922; doi:10.1021/acscentsci.6c00922)
Supplement: Supplementary file 1 [file oc6c00922_si_001.pdf]

Supplementary Information for

Ninhydrin as a covalent warhead for chemical proteomic-enabled discovery and selective engagement of reactive arginines

Andrew K. Ecker[1,2*], Andreas Langen[1*], Chloe S. Fields[1], José L. Montaño[1], Minh Tran[1], Ian B. Seiple[1,2,3], Balyn W. Zaro[1]

1 - Department of Pharmaceutical Chemistry, University of California, San Francisco, San Francisco, United States
2 – Department of Chemistry, Scripps Research Institute, La Jolla, United States
3 - Cardiovascular Research Institute, University of California, San Francisco, San Francisco, United States
* equal contribution

To whom correspondence should be addressed: balyn.zaro@ucsf.edu, iseiple@scripps.edu

This PDF file contains:

Supplemental Figures **5**

List of Tables **13**

Methods **15**

Uncropped Gels **28**

Uncropped gel images from Figure 2B. *28*

Uncropped gel images from Figure 2C. *28*

Representative Traces **29**

Representative Fmoc-AA-OH consumption assay HPLC traces. *29*

Representative Intact Protein Mass Spectrometry Deconvoluted Spectra. *30*

Synthesis of Probes **31**

Compound SI-1 .... *31*

Nin-Alk *32*

Compound SI-2 .... *32*

4-Nin-Alk *33*

Compound SI-3 .... *33*

Compound SI-4 .... *34*

CA-Ind-Alk *35*

Compound SI-6 .... *36*

isoCA-Alk *36*

CA-Alk *37*

Compound SI-7 .... *38*

Compound SI-8 .... *38*

Compound Lig-Nin *39*

Spectral Data **40**

SI-1 1H *40*

SI-1 13C *41*

Nin-Alk 1H *42*

Nin-Alk 13C *42*

SI-2 1H *43*

SI-2 13C *43*

4-Nin-Alk 1H 44
4-Nin-Alk 13C 45
SI-3 1H 46
SI-3 13C 47
SI-3 19F 47
SI-4 13C 49
SI-4 19F 50
CA-Ind-Alk 1H 51
CA-Ind-Alk 13C 52
CA-Ind-Alk 19F 53
SI-6 1H 54
SI-6 13C 54
SI-6 19F 55
isoCA-Alk 1H 56
isoCA-Alk 13C 57
isoCA-Alk 19F 58
isoCA-Alk HSQC 59
CA-Alk 1H 60
CA-Alk 13C 60
CA-Alk 19F 61
CA-Alk HSQC 62
CA-Alk HMBC 63
Compound SI-7 1H 64
Compound SI-7 13C 64
Compound SI-8 1H 65
Compound SI-8 13C 65
Lig-Nin 1H 66
Lig-Nin 13C 66
Lig-Nin 19F 67
Lig-Nin COSY 67
Lig-Nin HSQC 68
CA-Alk: HPLC purity trace 254 nm 69
Iso-CA-Alk: HPLC purity trace 254 nm 69
Lig-Nin: HPLC purity trace 254 nm 70

Supplemental Figures

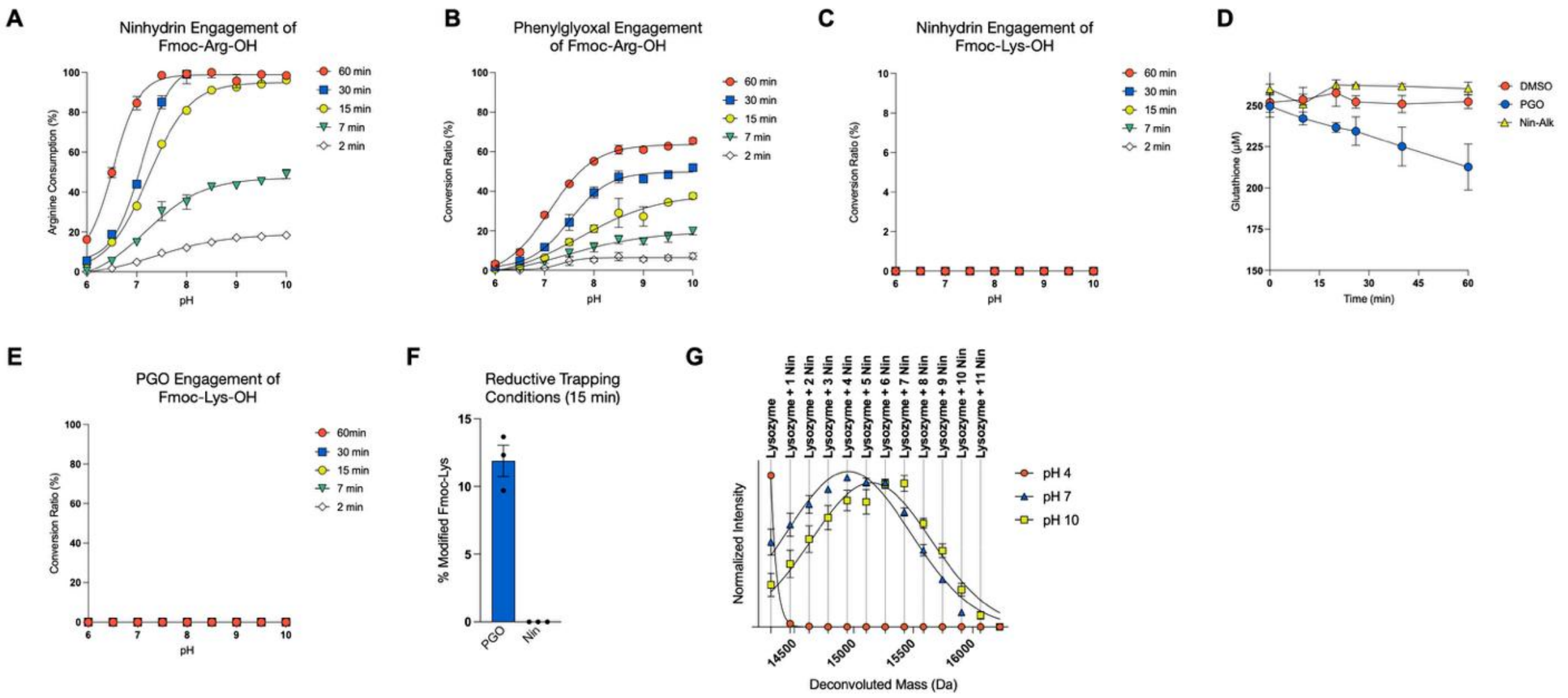

Figure S1 | Evaluation of ninhydrin as a covalent modifier of lysine and free thiols. A. Kinetic analysis of pH- and time-dependent consumption of Fmoc-Arg (500 µM) by ninhydrin (15 mM, 30x) in PBS at 20 °C. Arginine consumption was quantified by LC-MS over 2 to 60 minutes. Error bars represent SD. n = 3. **B.** Kinetic analysis of pH- and time-dependent consumption of Fmoc-Arg (500 µM) by phenylglyoxal (15 mM, 30x) in PBS at 20 °C. Arginine consumption was quantified by LC-MS over 2 to 60 minutes. Error bars represent SD. n = 3.**C.** Kinetic analysis of pH- and time-dependent consumption of Fmoc-Lys (500 µM) by ninhydrin (15 mM, 30x) in PBS at 20 °C. Lysine consumption was quantified by LC-MS over 2 to 60 minutes. Error bars represent SD. n = 3. **D.** Glutathione reactivity assay for arginine-reactive warheads. Compounds were incubated with reduced glutathione (250 µM) in assay buffer (100 mM Tris pH 9.0, 50% MeOH) at a final concentration of 500 µM. At the indicated time points (0–60 min), Ellman's reagent was added and residual free thiols were quantified via absorbance at 412 nm. Values reflect means of triplicate measurements with individual replicates shown. DMSO control (orange circles), phenylglyoxal (blue circles), **Nin-Alk** (yellow triangles). **E.** Kinetic analysis of pH- and time-dependent consumption of Fmoc-Lys (500 µM) by PGO (15 mM, 30x) in PBS at 20 °C. Lysine consumption was quantified by LC-MS over 2 to 60 minutes. Error bars represent SD. n = 3. **F.** Consumption of Fmoc-Lys (500 µM) by phenylgyoxal or ninhydrin (15 mM, 30x) in PBS supplemented with 30mM sodium cyanoborohydride at 20 °C for 15 minutes. Lysine consumption was quantified by LC-MS analysis of the UV trace at 300 nm. **G.** Assessment of ninhydrin engagement of lysozyme. Lysozyme is covalently modified by ninhydrin. Intact protein mass spectrometry analysis of Lysozyme (1.5 µM) and 100 µM ninhydrin at 37 °C for 30 minutes across pH 4.0, 7.0 and 10.0. Reactions were quenched by dilution into 0.1% aqueous TFA and analyzed by LC–MS on a Agilent 6230 TOF system. N =3 Error bars represent SEM. Representative deconvoluted mass spectra are in SI.

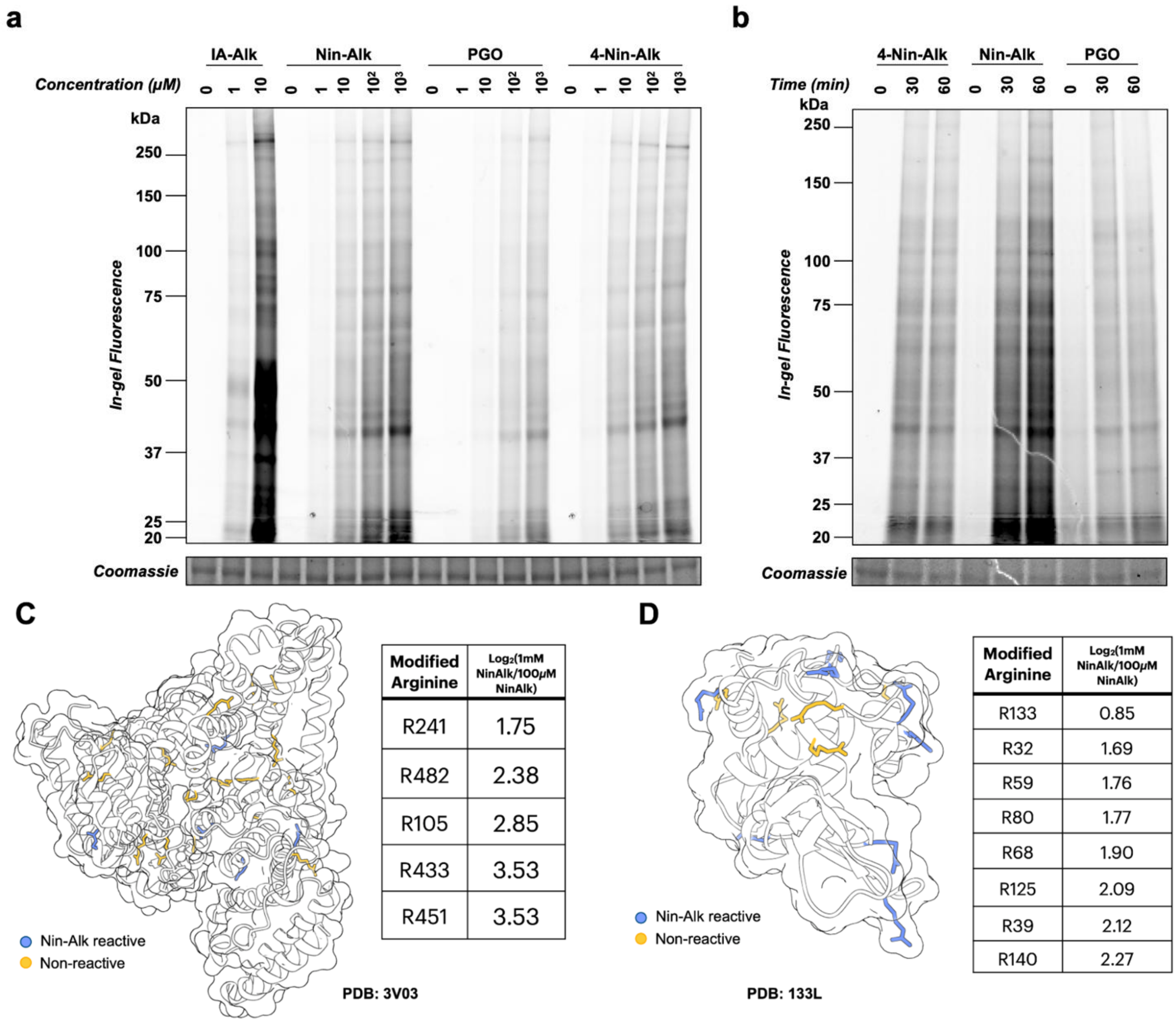

Modified Arginine	Log₂(1mM NinAlk/100µM NinAlk)
R241	1.75
R482	2.38
R105	2.85
R433	3.53
R451	3.53

Modified Arginine	Log₂(1mM NinAlk/100µM NinAlk)
R133	0.85
R32	1.69
R59	1.76
R80	1.77
R68	1.90
R125	2.09
R39	2.12
R140	2.27

Figure S2 | In-gel fluorescence scanning of cysteine and arginine-reactive probe labeling in lysates. A. Qualitative, gel-based assessment of proteome-wide reactivity of alkyne-bearing covalent probes in Mino cell lysate**.** Soluble proteome (1.5 µg µL⁻¹) was treated with each probe at the indicated concentration for 1 h, followed by CuAAC conjugation with TAMRA-N_3, separation by SDS–PAGE, and in-gel fluorescence scanning. **B.** Qualitative time-dependent reactivity of indicated probes in live cells. Mino cells were treated with 100 µM of the indicated probe for 0, 30, or 60 min, followed by lysis, CuAAC conjugation with TAMRA-N_3, separation by SDS–PAGE, and in-gel fluorescence scanning. Uncropped gel images are in SI. Identification of Nin-Alk reactive arginine residues on BSA (**C.**) and Lysozyme (**D.**) in Mino cell lysate doped with 1% (w/w) lysozyme and BSA. Highlighted residues indicate sites of Nin-Alk engagement, with corresponding Log_2(1mM/100µM). RAP analysis of the doped lysate quantified 5,257 unique arginine sites, indicating robust proteome-wide coverage is maintained.

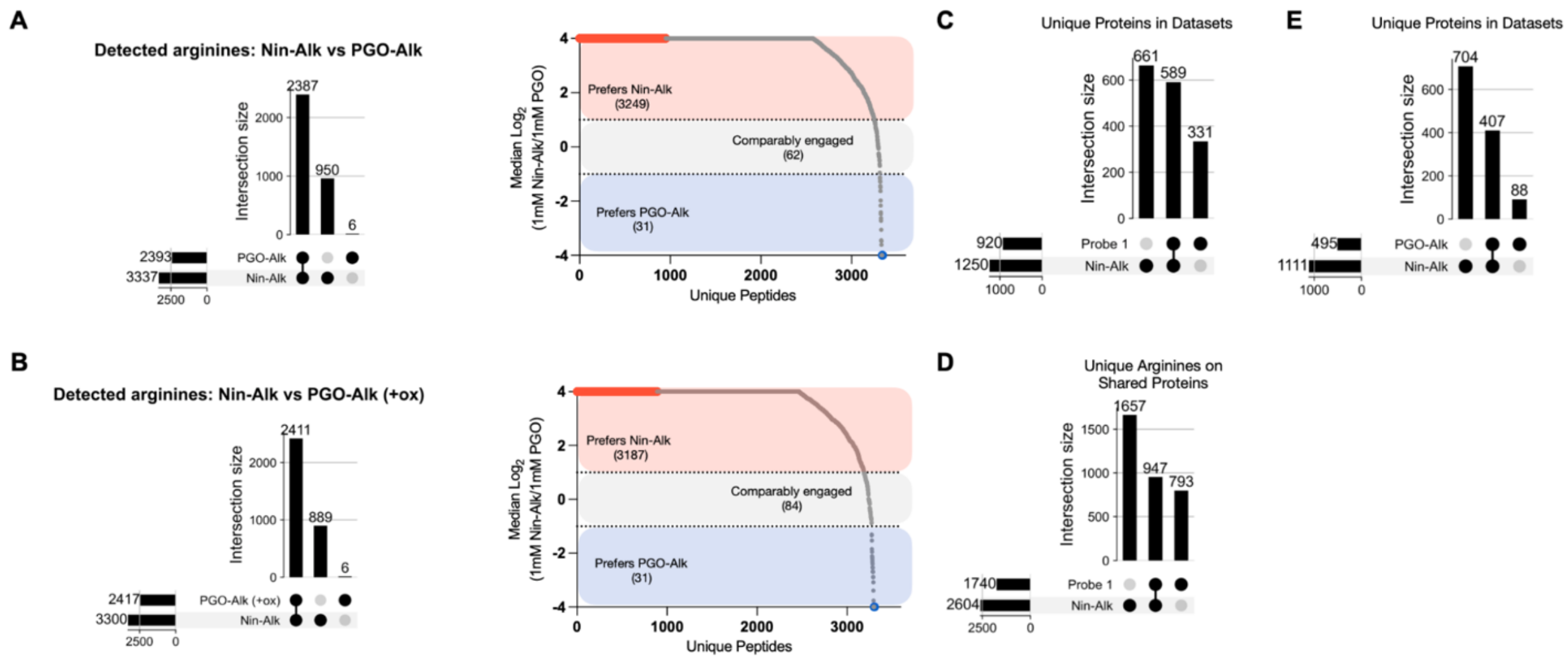

Figure S3 | Comparison of Nin-Alk and other arginine reactive probes. UpSet and waterfall plots for the closed search of the non-oxidized **PGO-Alk**-engaged and **Nin-Alk**-engaged arginines detected by RAP. High overlap was observed between **PGO-Alk** and **Nin-Alk**-engaged arginines (n = 2,387), with the majority preferentially engaged with a $\log_2$ ratio ≥ 1 by **Nin-Alk** (n = 3,249) relative to **PGO-Alk** (n = 31). **B.** Corresponding analysis for the closed search including the oxidized **PGO-Alk** species (+ox). Similar trends were observed (n = 3,306 total), with a higher preference for **Nin-Alk** (n = 3,187) relative to **PGO-Alk** (31). **C.** Overlap of unique proteins reported by Wang, et al. using **Probe 1** and Nin-Alk-based reactive arginine profiling (RAP) in A549 cells. A total of 661 unique proteins were identified in Mino cells by **Nin-Alk**, compared to 331 by Wang, et al., with 589 sites shared between datasets. **D.** Within shared proteins (n = 589), **Nin-Alk** uniquely identified 1,657 arginine sites compared to 793 reported by Wang, et al., with 947 sites shared among the shared proteins. **E.** Of proteins engaged by **PGO-Alk** and **Nin-Alk** in competition with **Lig-Nin**, **Nin-Alk** uniquely identified 704 proteins compared to only 88 identified by **PGO-Alk**, with 407 proteins shared between the probes.

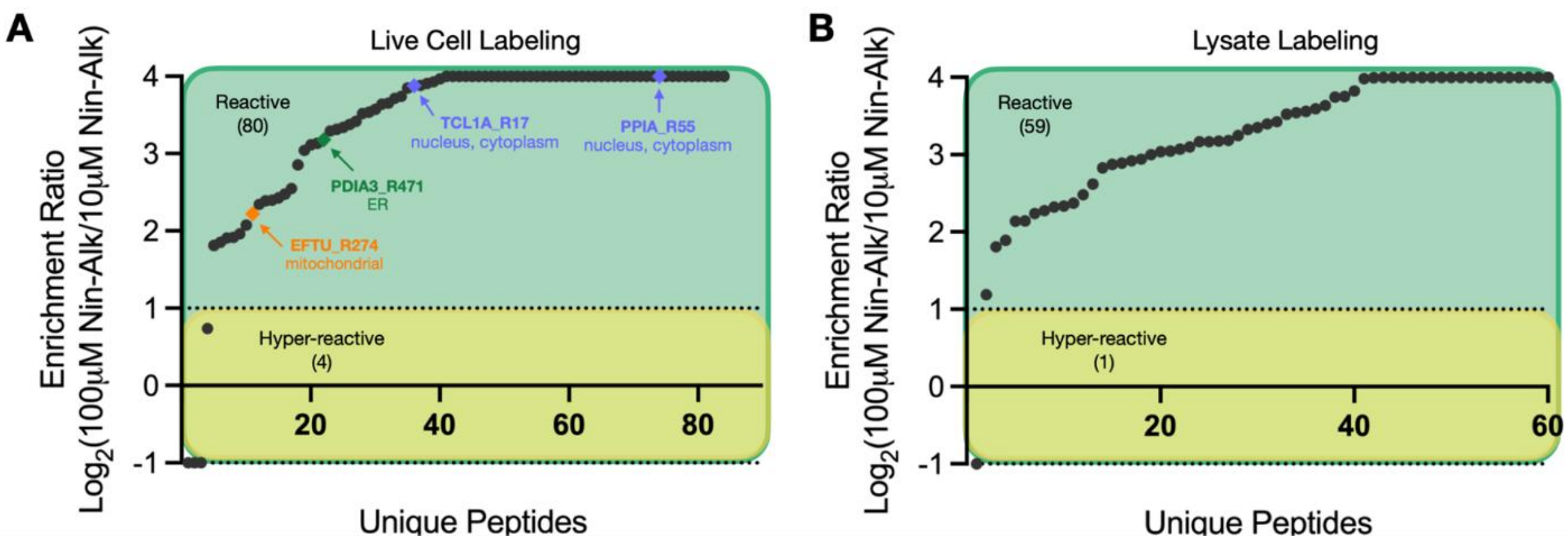

Figure S4 | Live-cell RAP profiling in Mino cells. A. Waterfall plot showing the distribution of median RAP enrichment ratios ($\log_2$100 μM **Nin-Alk**/10 μM **Nin-Alk**) obtained from live-cell labeling in Mino cells. Arginines with $\log_2 \geq 1$ were characterized as reactive (n = 80), while those with a $\log_2 < 1$ were designated hyper-reactive (n = 4). Representative proteins with their annotated subcellular localizations (UniProt) are highlighted, demonstrated labeling of arginines from distinct cellular compartments including the nucleus/cytoplasm (TCL1A, PPIA), endoplasmic reticulum (PDIA3), and mitochondria (EFTU). **B.** Waterfall plot showing distribution of median RAP enrichment ratios for lysate labeling of Mino cells at 100 μM and 10 μM of **Nin-Alk**. Despite the absence of cellular barriers and growth media, overall coverage remained modest (n = 60 reactive arginines), suggesting that the lower number of labeled sites observed in live-cell experiments is not solely attributable to probe permeability or intracellular accessibility.

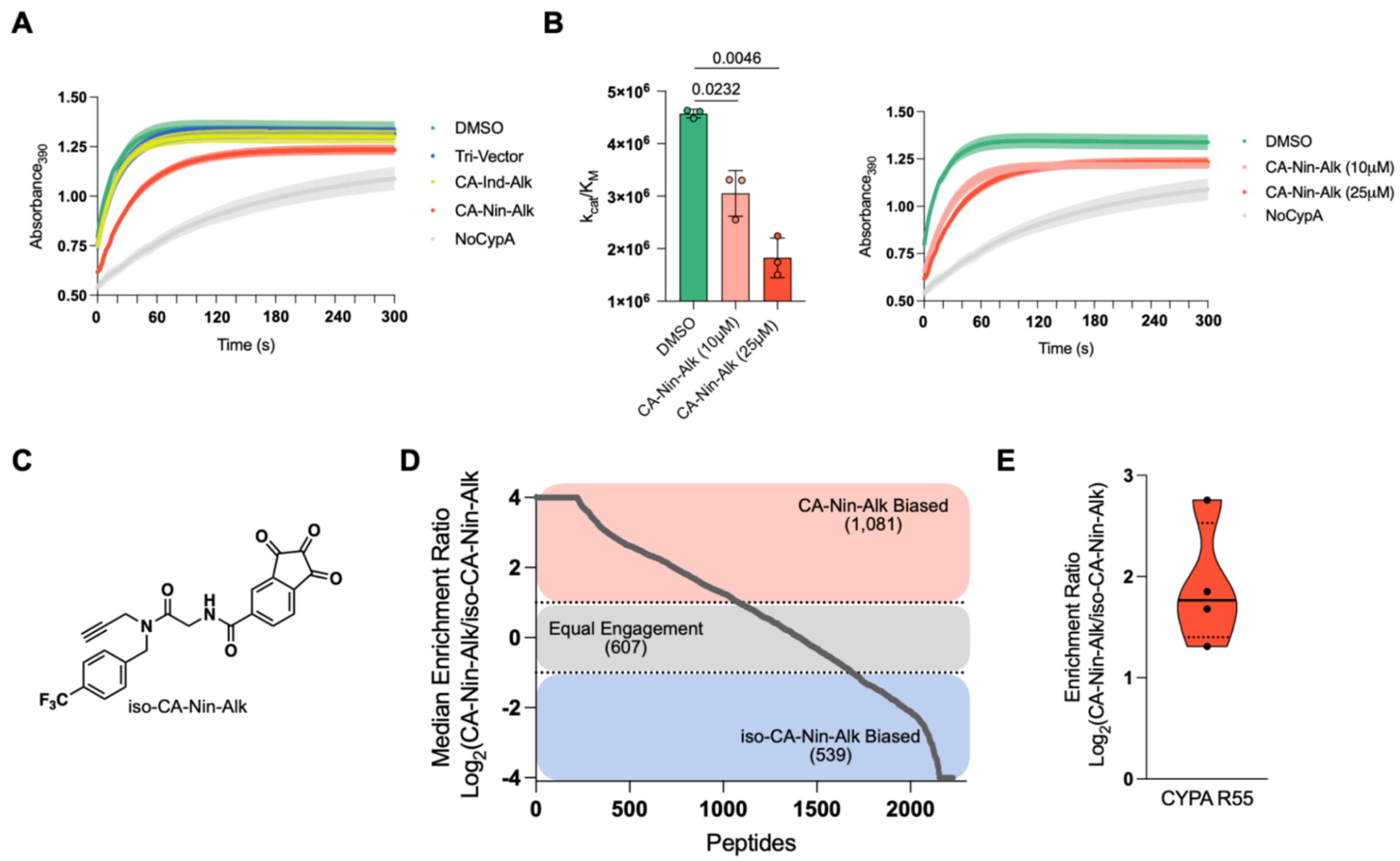

Figure S5 | Exploration of CA-Nin-Alk and its regioisomer. A. Representative absorbance traces (A_{390}) from the coupled peptidyl-prolyl isomerase assay over time (t = 300s). Solid lines represent mean values and shaded regions indicate ± s.d. (n = 3). No CypA condition reflects the uncatalyzed rate of substrate isomerization. **B.** CypA activity measured by a coupled peptidyl-prolyl isomerase assay. Pre-incubation of 1 µM recombinant CypA with **CA-Nin-Alk** decreases CypA catalytic efficiency (kcat/Km) in a dose-dependent manner compared to DMSO. Data represent mean ± s.d. (n = 3). Representative absorbance traces (A_{390}) from the coupled peptidyl-prolyl isomerase assay over time (t = 300s). Solid lines represent mean values and shaded regions indicate ± s.d. (n = 3). No CypA condition reflects the uncatalyzed rate of substrate isomerization. **C.** Chemical structure of the tri-vector alkyne **iso-CA-Nin-Alk**, a clickable analog previously shown to bind cyclophilin A (CypA) **D.** Waterfall plot depicting the regioselective enrichment of peptides labeled by **CA-Nin-Alk** and **iso-CA-Nin-Alk** in Mino cell lysate. Peptides identified by the RAP workflow were classified based on relative enrichment at 1 mM probe concentration in biological triplicates. Each point represents the $\log_2$ ratio of **CA-Nin-Alk** to **iso-CA-Nin-Alk** enrichment for an individual peptide. Peptides were grouped into **CA-Nin-Alk** biased (red, n = 1,081), **iso-CA-Nin-Alk** biased (blue, n = 539), and equally engaged (gray, n = 607) categories **E.** Relative enrichment of CypA R55 between **CA-Nin-Alk** and **iso-CA-Nin-Alk** as determined by RAP. n = 4 replicates.

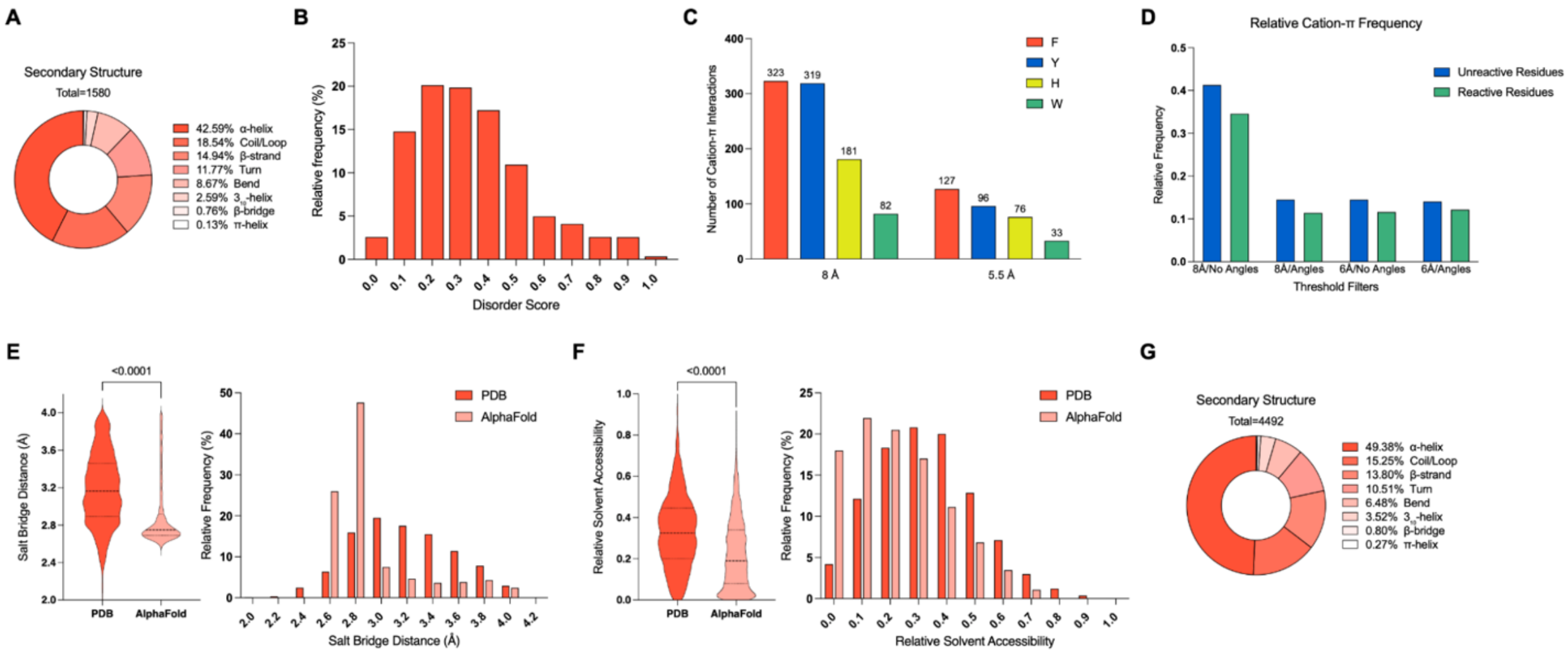

Figure S6 | Comparative analysis of intermolecular interactions surrounding reactive arginines. A. Secondary structure distribution of all reactive arginines based on DSSP annotation of PDB models available (n = 1580 residues). **B.** Histogram of predicted intrinsic disorder scores at modified arginine sites, calculated using IUPred2A. Scores > 0.5 indicate disordered regions. (n = 3630) **C.** Quantification of cation–π interactions between reactive arginines and aromatic residues in PDB deposited structures. Interaction counts are shown at distance thresholds of 8.0 or 5.5 Å, measured from the guanidinium CZ atom to the centroid of the aromatic ring. Geometric constraints were applied for inclusion of parallel interactions (face-on) when the angle between the CZ-to-centroid vector and the normal vector of the aromatic plane was ≤ 30°, and T-shaped interactions (edge-on) when deviation from 90° was ≤ 20°(**Table 7**). **D.** Frequency of arginines engaged in cation–π interactions across reactive (green) and unreactive (blue) datasets, based on AlphaFold-predicted models and varying distance (6 Å or 8 Å) and angle filters. No significant enrichment or depletion of cation–π interactions was observed among reactive residues. **E.** Comparison of salt bridge distances between reactive arginines and acidic residues in AlphaFold (light coral, n = 1594) versus experimentally determined PDB structures (terra cotta, n = 1445), showing generally shorter predicted distances in AlphaFold models. **F.** Comparison of RSA values for all reactive arginines across AlphaFold (light coral, n = 4309) and PDB (terra cotta, n = 1649) models reveals a modest shift toward higher accessibility in AlphaFold predictions. **G.** Secondary structure distribution of all reactive arginines based on DSSP annotation of AlphaFold models (n = 4492 residues). The majority localize within α-helices (49.38%), followed by coil/loop regions and β-strands.

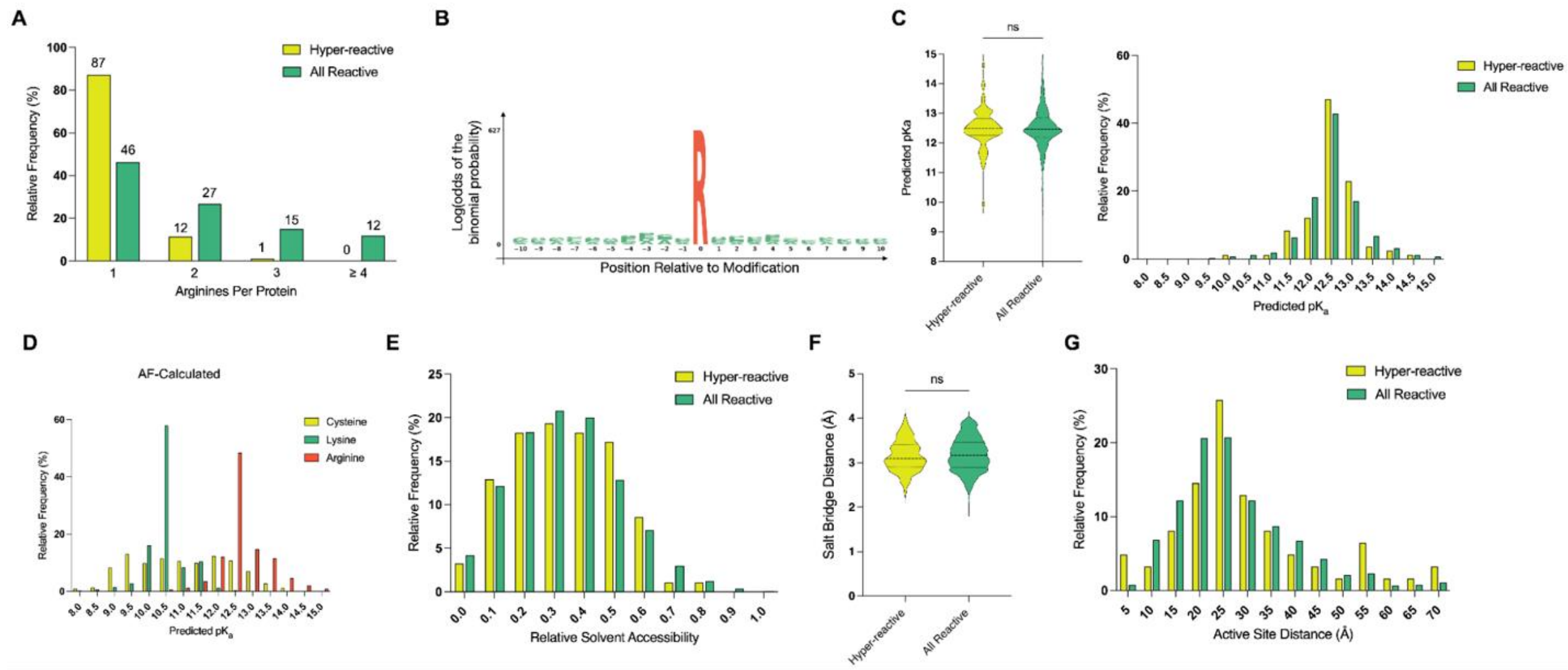

Figure S7 | Comparison of biophysical and functional features between reactive and hyper-reactive arginine residues. A. Distribution of reactive and hyper-reactive arginines per unique protein. **B.** pLogo motif analysis of the ±10-residue sequence window surrounding all modified arginines (foreground: 6,913 modified sites; background: 773,893 human arginines. **C.** Distribution of predicted pKa values for hyper-reactive (n = 85) and all reactive arginine residues (n = 1570), calculated using PROPKA3[6] and the PDB. Welch's t-test (two-tailed, parametric) **D.** Predicted pKa distributions for reactive arginines(orange, n = 4334), cysteines(yellow, n = 3988), and lysines (green, n = 3863) using AlphaFold structures. Arginine predictions are from the current dataset, cysteine data was based on matched cell analysis with Mino cell lysate and iodoacetamide alkyne as the electrophile and lysine data are derived from previously reported reactivity profiles from Hacker et al. (2017). No significant pKa shift distinguishes hyper-reactive from reactive residues across any residue class. **E.** Predicted relative solvent accessibility (RSA) of hyper-reactive (n = 93) and reactive arginines (n = 1649), computed using the Shrake–Rupley algorithm on PDB structures, indicating a shared preference for surface exposure(**Table 19**). **F.** Violin plot showing the distribution of salt bridge distances between guanidinium nitrogen atoms of hyper-reactive (n = 101) or all reactive arginines (n = 1445) and carboxylate oxygens of acidic residues (Asp/Glu) in PDB models(**Table 17**). Welch's t-test (two-tailed, parametric) **G.** Distribution of distances from reactive (n = 777) and hyper-reactive (n = 62) arginine residues to the geometric center of UniProt-annotated active site residues, calculated using AlphaFold-predicted structures. Only arginines with local pLDDT scores greater than 75 were included in the analysis.

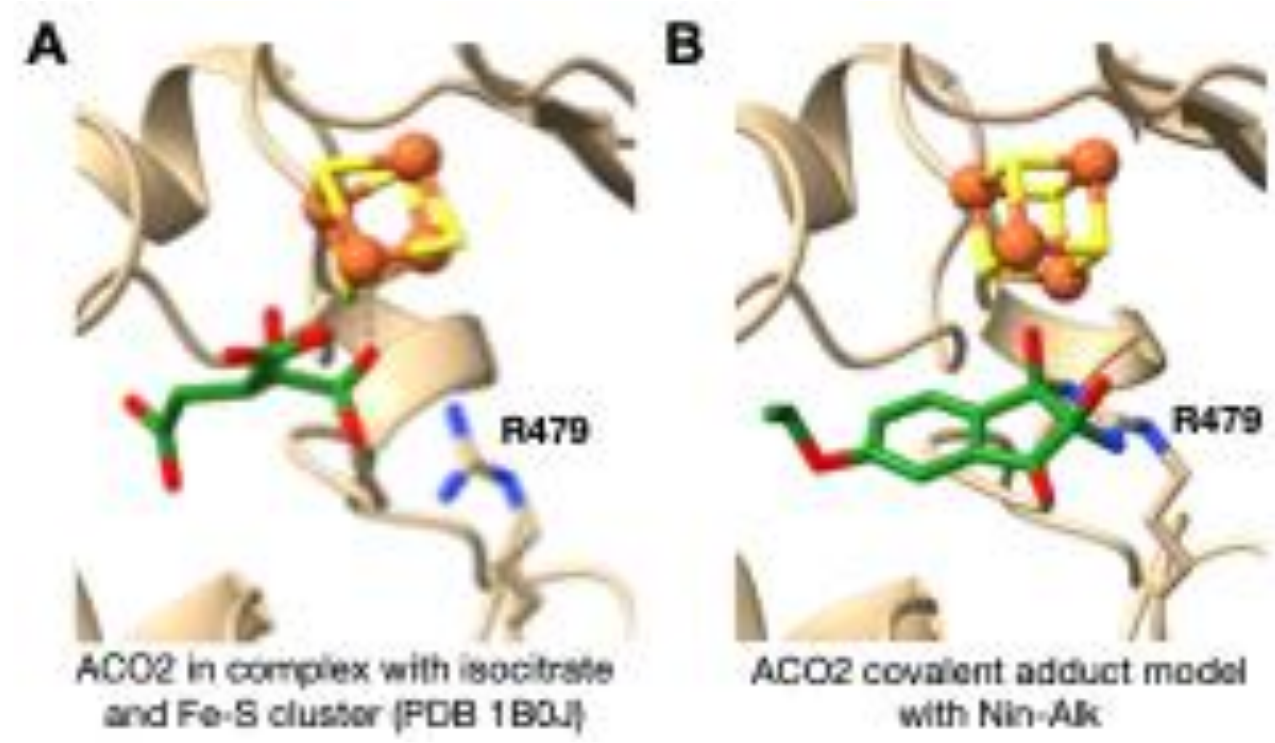

Figure S8 | Exploration of covalent inhibition of aconitase by ninhydrin at R479. A. Crystal structure of mitochondrial aconitase (ACO2) bound to isocitrate and a [4Fe-4S] cluster, highlighting the proximity of R479 to the active site (PDB: 1B0J). **B.** Covalent docking model of **Nin-Alk** to R479 of ACO2, showing spatial alignment with the native isocitrate ligand.

List of Supplemental Tables

Table S1_OpenSearch_NinAlkOnly: Open Search output listing enriched mass shifts detected in the RAP dataset via MSFragger/PTM-Shepherd.

Table S2_NinAlkvPGO(merged): Closed search output of 5-Nin-Alk vs PGO (for either mass) labeling at 1.0 mM. Includes peptide-level quantification and calculated R values.

Table S3_OpenSearch_PGOAlkvNinAlk: Open Search output listing enriched mass shifts detected in a comparative dataset via MSFragger/PTM-Shepherd.

Table S4_NinAlkvPGOAlk(-ox): Closed search output of 5-Nin-Alk vs PGO-Alk (with no oxidation) labeling at 1.0 mM. Includes peptide-level quantification and calculated R values.

Table S5_NinAlkvPGOAlk(+ox): Closed search output of 5-Nin-Alk vs PGO-Alk (with oxidation) labeling at 1.0 mM. Includes peptide-level quantification and calculated R values.

Table S6_NinAlk_A549: Closed search output of Nin-Alk labeling in A549 cell lysates at 100 μM and 1.0 mM. Includes peptide-level quantification and calculated R values.

Table S7_PGOAlk_NinComp: Closed search output of DMSO v Nin competition of PGO-Alk labeling at 1.0 mM. Includes peptide-level quantification and calculated R values.

Table S8_NinAlk_PGOComp: Closed search output of DMSO v PGO competition of Nin-Alk labeling at 1.0 mM. Includes peptide-level quantification and calculated R values.

Table S9_NinAlk_LigNinComp: Closed search output of DMSO v LigNin (50 μM) competition of Nin-Alk labeling at 1.0 mM. Includes peptide-level quantification and calculated R values.

Table S10_PGOAlk_LigNinComp: Closed search output of DMSO v LigNin (50 μM) competition of PGO-Alk labeling at 1.0 mM. Includes peptide-level quantification and calculated R values.

Table S11_NinAlk_Mino: Closed search output of Nin-Alk labeling in Mino cell lysates at 100 μM and 1.0 mM. Includes peptide-level quantification and calculated R values.

Table S12_ProximitySearch: Results of ligand proximity search in experimental structures for reactive arginines; reports all ligands within 3 Å if any are found, otherwise reports no ligands found.

Table S13_CypAActivity: Raw data from CyclophilinA activity assay.

Table S14_CA-Nin-Alk_100uM: Quantitative data for CA-Nin-Alk labeled arginines at 100 μM from RAP experiment.

Table S15_CA-Nin-Alk_10uM: Same as Table 13 but for 10 μM treatment to assess dose dependence of arginine reactivity.

Table S16_CA-Nin-Alkviso-CA-Nin-Alk: Comparison of arginine labeling between CA-Alk and 5CA-Alk. Contains peptide-level intensities from multiple replicates to show fold enrichment between regioisomers.

Table S17_AlphaMissense: AlphaMissense predictions for mutational pathogenicity of modified arginines, with pathogenicity scores and classification.

Table S18_ArgOnlyTargets: Proteins harboring reactive arginines and not reactive cysteines – pharos annotations and disease implications

Table S19_PDBSecondaryStructure: Secondary structure assignments of reactive arginines across all available PDB structures.

Table S20_InterPDBMoleInt: Intermolecular interactions (cation-π, salt bridges) involving reactive arginines in experimental PDB structures, including distances and interaction geometry.

Table S21_AFSaltBridges: AlphaFold-based salt bridge assignments between arginines and acidic residues, with pLDDT and distance info.

Table S22_RSAandPKA_PDB: Solvent-accessible surface area (SASA), relative solvent accessibility (RSA), predicted pKa values from PROPKA for reactive arginines and active site distances mapped to PDB structures.

Table S23_AF_RSA: SASA, RSA, and pLDDT for all reactive arginines in AlphaFold models. Used to analyze exposure and confidence across the proteome.

Table S24_AFSecondaryStructure: Secondary structure assignments of reactive arginines across all AlphaFold structures.

Table S25_ArgAFpKA: Predicted pKa values from PROPKA for reactive arginines mapped to AlphaFold structures.

Methods

Density Functional Theory (DFT) Calculations Initial molecular models were prepared using Avogadro[1]. All calculations were performed using GAMESS[2] on ChemCompute.org. Geometry optimization was performed with the PM3 basis set, restricted Hartree-Fock (RHF) molecular orbital method, and B3LYP DFT functional. Solvent effects were modeled using the water PCM solvent model on a single processor core. The optimized structures were subjected to a single-point energy calculation with the 6-311G** basis set. All output models were reviewed in Avogadro for realistic geometry, and the delta G free energy in solvent between the hydrated and dehydrated states (with an additional water molecule) for both phenylglyoxal and ninhydrin was calculated to determine the relative equilibrium constants.

Fmoc- Arginine and Lysine Kinetics Assay In triplicate, 190 µL of PBS buffer (pH 6.0 – 10.0, in 0.5 pH unit increments) was allotted into 27 Eppendorf tubes. A 5 µL aliquot of a 20 mM stock solution of Fmoc-Arg-OH(or Fmoc-Lys-OH) in DMSO was added to each tube, mixed thoroughly, and briefly centrifuged to ensure complete pooling of the solution. A 600 mM Ninhydrin solution (1:1 DMSO/DI water) was prepared alongside a 2 M HCl quenching solution. All tubes were pre-opened, and a timer, vortex mixer, waste container, and fresh pipette tips were arranged for efficient handling. Reactions were initiated by adding 5 µL of Ninhydrin solution to the first tube while starting the timer, followed by immediate capping and vortex mixing. This process was repeated every 10 seconds for the subsequent tubes, ensuring all reactions were initiated within 4.5 minutes. At designated time points, the reactions were quenched by sequentially adding 10 µL of 2 M HCl to each tube, following the same 10-second interval sequence as for reaction initiation. Samples were then centrifuged, and 200 µL of the supernatant was carefully transferred using gel-loading tips into pre-labeled LC-MS vials with conical inserts, ensuring minimal air bubble formation. Samples were analyzed using a Waters Acquity UPLC SQD2 detector LC-MS system on a polar gradient method (20–55% acetonitrile/water + 0.1% formic acid). The UV detection window was set to 300 nm for Fmoc absorbance, and free arginine (SM) and adduct formation were identified based on the extracted ion chromatograms. Peak integrations at 300 nm were used to calculate the conversion ratio of reactants to products.

Cysteine Reactivity Assay L-cysteine was prepared at 250 µM in assay buffer (100 mM Tris, pH 9.0, 50% MeOH). In triplicate, 100 µL of the cysteine solution was dispensed into each well of a clear, flat-bottom 96-well plate (Costar). Inhibitors were added as 100X stocks (1 µL per well; 500 µM final concentration) or with DMSO vehicle control, and reactions were incubated at room temperature for the indicated times (0-60min). Following incubation, Ellman's reagent (10 µL of a 50mM stock in DMSO; 5mM final concentration) was added, and plates were incubated for 10 minutes at room temperature. Absorbance was measured at 412nm in a plate reader (Spectramax M5). Free cysteine concentrations were quantified using a standard curve, and second-order rate constants were calculated as previously described[3].

Intact Protein Mass Spectrometry Reactions were performed with 1.5 µM BSA or Lysozyme in PBS, incubated with 150 µM ninhydrin at 37 °C for 30 minutes. Reactions were quenched by a dilution to 0.1% TFA (aq) and analyzed by intact protein LC/MS using an Agilent 6230 TOF LC/MS with a Dual AJS ESI ion source. Agilent 6230 TOF system equipped with an Agilent RLRP-S

1000Å 5 µm 50x2 1MM column. The mobile phase was a linear gradient of 5–90% acetonitrile/water + 0.1% formic acid over 4 minutes with a flow rate of 300 µL/min.

Glutathione reactivity assay Reduced glutathione (GSH) was prepared at a concentration of 250 µM in assay buffer (100 mM Tris pH 9.0, 50% MeOH as co-solvent). In triplicate, 100 µL of GSH (250 µM) in assay buffer was added to each well of a clear, flat-bottom 96-well plate (Greiner Bio-one). To each well, 1 µL of 100x inhibitor stocks in DMSO (final concentration 500 µM) or DMSO vehicle was added, and the plate was incubated at room temperature for the indicated time (0–60 min). Following incubation, Ellman's reagent (Thermo) (10 µL, 50 mM stock in DMSO, final concentration 5 mM) was added, and the reaction was incubated at room temperature for 10 minutes. Absorbance was measured at 412 nm using a plate reader (Cytation 5, BioTek). The remaining concentration of free thiols was quantified using a glutathione standard curve.[3]

Cell Culture Mino cells were cultured under standard conditions (37 °C, 5% CO_2) in T175 flasks. Cells were revived from frozen stocks stored in liquid nitrogen. Cryovials containing the cells were rapidly thawed in a 37 °C water bath and immediately transferred to pre-warmed complete culture media. To remove residual DMSO, cells were centrifuged at 300 × g for 5 min, resuspended in fresh culture media, and plated in appropriate flasks. Cells were allowed to recover for 24–48 hours before the first media change and were subsequently maintained under routine culture conditions.

Cells were maintained in non-treated T175 flasks in Cytiva RPMI-1640 medium (no HEPES, stored at 4 °C) supplemented with 10% fetal bovine serum (HyClone), L-glutamine (100 µg mL^{-1}), 0.05 mM β-mercaptoethanol, and penicillin-streptomycin (100 µg mL^{-1}). Cells were routinely passaged every 2–3 days when they reached a density of ~8 × 10^5 cells per mL to maintain exponential growth. Passaging was performed by centrifuging the culture at 300 × g for 5 min, discarding the supernatant, and resuspending the cell pellet in fresh pre-warmed medium at a 1:3 dilution.

Cells were harvested after three passages to ensure robust growth and adaptation to culture conditions. Mino cells were collected from suspension culture, pelleted at 300 × g for 5 min, and washed once with ice-cold PBS. Washed cell pellets were resuspended in ice-cold Dulbecco's phosphate-buffered saline (DPBS) and sonicated on ice (2 × 20 pulses, 20% amplitude, 1 s pulse rate) using a tip sonicator. The lysed suspension was centrifuged at 21,000 × g for 20 min at 4°C, and the supernatant was transferred to a lo-bind tube. Protein concentrations were determined using the bicinchoninic acid (BCA) assay (Thermo Scientific), and the resulting lysate was used fresh for subsequent experiments.

Gel-Based Probe Reactivity Profiling For screening reactivity in lysate, 50 µL of freshly prepared cell lysates (~1.5 mg mL^{-1} total protein) were treated as indicated in PBS buffer (pH 7.4) at room temperature. Lysates were dosed with a 100X probe in DMSO or an equal volume of DMSO as a control. Following probe treatment, CuAAC click chemistry was performed by sequentially adding 2 µL TAMRA-azide (5 mM, stock in DMSO), 2 µL tris(2-carboxyethyl)phosphine hydrochloride (TCEP) (50 mM, freshly prepared in water), 6 µL tris[(1-benzyl-1-H-1,2,3-triazol-4-yl)methyl]amine (TBTA) (1.7 mM, 3:1 tert-butanol:DMSO), and 2 µL

CuSO4•5H2O (50 mM in water) with vortexing between additions. The reaction was incubated at room temperature for 1 h with gentle mixing.

For live-cell screening, 10^6 cells were plated in 3 mL of complete cell growth medium (as described above) in a single well of a 12-well culture plate (Costar). Wells were treated with either 1000X of either probe or vehicle (0.1% v/v) for 1 h under standard conditions (37°C, 5% CO_2). Immediately following labeling, cells were washed 3X with 5 mL DPBS, pelleted, and flash-frozen in LN_2 and stored at -80°C overnight. The following day, pellets were lysed and protein concentrations were normalized to 50 µg (1 mg/mL). Click reactions were then performed under the same conditions as above.

Reactions were quenched by adding 250 µL of ice-cold methanol, followed by incubation at -20°C for at least 2 hours. Precipitated proteins were pelleted by centrifugation (10,000 × g, 10 min, 4°C), and the supernatant was discarded. Tubes were inverted and allowed to dry for 30 minutes to ensure complete evaporation of methanol. Protein pellets were resuspended in 75 µL of 4% SDS in PBS (pH 7.5) and bath sonicated (35 kHz, 10 min) to achieve complete solubilization. Following resuspension, 25 µL of 4× Laemmli loading buffer (Bio-Rad, containing 10% β-mercaptoethanol) was added, and samples were vortexed gently before boiling at 95°C for 5 minutes. After cooling to room temperature, 20 µg of protein per lane was loaded onto a Tris-Glycine SDS-PAGE gel (Bio-Rad, Criterion TGX) for separation. Fluorescently labeled proteins were visualized using a Bio-Rad ChemiDoc fluorescence scanner, and total protein staining with Coomassie was performed for normalization.

RAP Workflow For labeling of cell lysates, 250 µL freshly prepared cell lysate at 2 mg mL^{-1} protein concentration was treated in lo-bind tubes with either 100 µM probe or 1.0mM probe for 1 hr at 37 °C in pairs. Following probe treatment, CuAAC click chemistry was performed by sequentially adding 25 µL isoDTB tags (light tags to the 100 µM treated, heavy tags to the 1.0mM treated, (Vector Labs, CCT-1565-L and CCT-1565-H, 10 mM, stock in DMSO), 25 µL tris(2-carboxyethyl)phosphine hydrochloride (TCEP) (50 mM, freshly prepared in water), 75 µL tris[(1-benzyl-1-H-1,2,3-triazol-4-yl)methyl]amine (TBTA) (1.7 mM, 3:1 tert-butanol:DMSO), and 25 µL CuSO4•5H2O (50 mM in water) with mixing between additions. For competition workflows, the heavy channel was pre-treated with indicated warheads, and the light channel was pre-treated with DMSO vehicle control. Following pre-treatment, lysates were labeled with indicated probes and then clicked to heavy and light tags as specified above.

The reaction was incubated at room temperature for 1 hour with gentle mixing. Protein samples were solubilized by adding 200 µL of 10% SDS in PBS, gently mixed and individually sonicated (35 kHz, 2 min) to dissolve any precipitate formed during the click chemistry reaction. Equal amounts of samples were combined in a 2 mL lo-bind Eppendorf tube and treated with 1 µL of benzonase, followed by incubation at 37°C for 30 minutes at 800 rpm.

For labeling of live cells, 10^7 Mino cells were concentrated to 3 mL in complete RPMI-1640 (Cytiva). Media was then supplemented with 1,000X stocks of either probe or vehicle (final concentration 0.1% v/v). Cells were kept under standard conditions (37°C, 5% CO_2) in T25 flasks for 1 hour. Following labeling, cells were immediately washed with 3X 10 mL DPBS, concentrated,

flash-frozen in LN_2, and stored at -80°C overnight. The following morning, cell pellets were then lysed in DPBS and centrifuged (10,000 × g; 10 min) and kept on ice. The soluble fraction was separated, and the remaining precipitated membrane fraction was re-suspended in DPBS. Both fractions were quantified via BCA and concentrations of respective channels were then normalized for click reactions and solubilized under the same conditions as mentioned above.

For protein precipitation and cleanup, SP3 bead purification was employed. 200 µL Sera-Mag SpeedBeads (GE45152105050250, GE65152105050250), were combined in a 1:1 ratio, washed three times with 1 mL water, and resuspended in 250 µL of water before being added to the sample. After incubation at room temperature with shaking at 1000 rpm for 5 minutes, 1 mL of absolute ethanol was added, and the sample was incubated under the same conditions for another 5 minutes. The beads were collected on a magnetic rack, and the supernatant was removed. Beads were washed three times with 1 mL of freshly prepared 80% ethanol.

To prepare proteins for digestion, the beads were resuspended in 500 µL of fresh 2 M urea in 0.5% SDS in PBS. Proteins were reduced with 10 mM DTT at 65°C for 15 minutes and then alkylated with 20 mM iodoacetamide at 37°C for 30 minutes at 500 rpm. The proteins were re-precipitated with 1 mL ethanol and subjected to the same washing steps as before.

Proteins were resuspended in 500 µL of fresh 2 M urea in PBS, and trypsin was added at a 1:100 enzyme-to-protein (w/w) ratio for overnight digestion at 37°C with shaking at 500 rpm. Following digestion, peptides were transferred to a 15 mL falcon tube containing 10 mL acetonitrile to achieve >95% final volume ACN. Samples were incubated at room temperature with shaking at 1000 rpm for 10 minutes before peptides were collected by centrifugation at 1600 rcf for 5 minutes. The pellet was washed three times with 1 mL ACN, then transferred to a 1.5 mL lo-bind Eppendorf tube. The samples were placed on a magnetic rack and washed once more in 1 mL fresh ACN. After magnetic separation, ACN was removed and peptides were eluted twice using 100 µL of 2% DMSO in water, incubated at 37°C for 30 minutes at 1000 rpm.

For enrichment, 250 µL of high-capacity NeutrAvidin resin (Thermo Fisher, 29204) was washed three times with 10 mL of IAP buffer (50 mM MOPS pH 7.4, 10 mM sodium phosphate, and 50 mM NaCl), then resuspended in a buffer volume adjusted to achieve a final peptide concentration of 0.2 µg/µL. Peptides were incubated with the resin for 2 hours at room temperature on a rotator, then washed twice with PBS and twice with water, with centrifugation at 1600 rcf between each wash. The resin was transferred to a bio-spin column (Bio-Rad, 7326204), washed twice with 500 µL of water, and centrifuged at 800 rcf for 30 seconds.

Enriched peptides were eluted in 60 µL of 80% acetonitrile in water containing 0.1% formic acid, incubated for 10 minutes at room temperature, then centrifuged at 1600 rcf for 1 minute. This elution was repeated at 72°C for 10 minutes at 300 rpm, and a final elution was performed at room temperature, followed by centrifugation at 2600 rcf for 2 minutes. The combined eluates were dried using a SpeedVac.

Crude peptides were then resuspended in 22 µL ACN and sonicated (35 kHz, 10 min). 425 µL water and 2.3 µL trifluoroacetic acid were then added followed by another sonication. C18 resin

(Thermo Fisher, 89870) was activated twice with 200 µL of 50% methanol in water, followed by three washes with 5% ACN in water containing 0.1% TFA. Resin was then placed into 2 mL lo-bind tubes for sample loading. Samples were then added onto the resin over three 150 µL additions. After complete loading, 200 µL of flowthrough was re-added to the resin twice to ensure complete binding. Samples were then washed three times as mentioned before. All centrifugation steps were performed at 300 rcf for 15 seconds. Samples were then eluted twice with 20 µL 70% ACN in water containing 0.1% formic acid after a 1 minute incubation (1600 rcf, 1 min). A third elution was performed similarly, but at 2600 rcf for 2 minutes. Eluted, desalted peptides were then dried using a SpeedVac.

Samples were resuspended in 20 µL LC-LOAD (PreOmics) followed by vortexing and sonication (35 kHz, 10 min). Samples were centrifuged (21000 rcf, 10 min) and 15 µL were transferred into MS vials (Thermo Fisher, 200 046) for analysis by mass spectrometry.

Mass Spectrometry Data Acquisition A nanoElute2 was attached in line to a timsTOF Pro2 equipped with a CaptiveSpray Source (Bruker, Hamburg, Germany). Chromatography was conducted at 40°C through a 25 cm reversed-phase C18 column (PepSep, 1893476) at a constant flow rate of 0.5 µL min^{-1}. Mobile phase A was 98/2/0.1% water/MeCN/formic acid (v/v/v, Thermo Fisher, LS118, LS120) and phase B was MeCN with 0.1% formic acid (v/v). During a 120 min method, peptides were separated by a 3-step linear gradient (4% to 40% B over 95 min, 40% to 60% B over 10 min, 60% to 90% B over 10 min) followed by a 10 min isocratic flush at 95% before washing and a return to low organic conditions. Experiments were run as data-dependent acquisitions with ion mobility activated in PASEF mode. MS and MS/MS spectra were collected with m/z 100 to 1700 and ions with z = +1 were excluded.

FragPipe Open Search To comprehensively survey mass shifts present in peptides across the dataset, an Open Search was performed using MSFragger within FragPipe v22.0. The search was configured with the following parameters: precursor mass range −10 to +850 Da, fragment mass tolerance 40 amu, isotope error set to 0, and enzyme set to “trypsin” with cleavage after “KR” and not before “P” (C-terminal specificity). Enzymatic digestion was assumed, allowing up to 2 missed cleavages. Minimum and maximum peptide lengths were set to 7 and 50 residues, respectively, and peptide mass range from 500 to 5000 Da. N-terminal methionine clipping was enabled, and a minimum of 15 peaks and a 0.01 intensity ratio were required per spectrum. Fragment ions considered included *b* and *y* series, with a maximum fragment charge of 2+. Calibration and parameter optimization were disabled, and deisotoping and de-neutralloss filtering were enabled. No fixed modifications were applied, and variable modifications were included but not enforced in the initial search (used only for localization and PTM profiling downstream).PeptideProphet was enabled with the following command-line options: --nonparam --expectscore --decoyprobs --masswidth 1000.0 --clevel -2, and results from multiple replicates were combined. PTMProphet was disabled. ProteinProphet was run with the setting --maxppmdiff 2000000. PTM-Shepherd was enabled for post-processing and mass shift profiling. Peak picking parameters were set to a smoothing factor of 2 bins, prominence ratio of 0.3, and peak picking width of 0.002 Da. Precursor mass tolerance was 0.01 Da. Fragment ion types considered for modification annotation were *b* and *y*, with a maximum fragment charge of 2+. PTM-Shepherd was run with default UniMod annotations disabled to allow unsupervised delta mass discovery,

and glycan annotation was enabled but no glycan database was used. Glycan delta masses were removed before peak picking. Normalization of mass shifts was performed across PSMs (not scans). Report generation was enabled with the following options: --sequential --mapmods --prot 0.01. MS1-based quantification, TMT-Integrator, and Spectral Library generation were disabled. For downstream analysis, the global.modsummary.tsv file from PTM-Shepherd was used to extract the number of PSMs associated with each observed mass shift. These counts were plotted against the theoretical mass shift to visualize the landscape of modifications in the range of +200 to +750 Da.

FragPipe Closed Search Mass spectrometry data were analyzed using FragPipe v22.0. Closed Search was performed using MSFragger with the following parameters: precursor mass tolerance −50 to +50 ppm, fragment mass tolerance 50 amu, isotope error set to 0/1/2, enzyme name “stricttrypsin”, cleavage at “KR” but not before “P” (C-terminal cleavage), enzymatic cleavage with up to 2 missed cleavages, peptide length between 7 and 50 residues, and peptide mass range from 500 to 5000 Da. Fragment ion types included *b* and *y* ions, with a maximum fragment charge of 2+. N-terminal methionine clipping was enabled. The number of variable modifications per peptide was limited to 4, and the number of allowed variable modification combinations was capped at 5000. Spectra were required to have a minimum of 15 peaks and a minimum peak intensity ratio of 0.01. Mass calibration was set to “Mass Calibration and Parameter Optimization,” and deisotoping and de-neutralloss processing were enabled.

Variable modifications included oxidation of methionine (+15.9949 Da), N-terminal acetylation (+42.0106 Da), and a pair of arginine labels (+695.2942 Da and +701.3014 Da) each with a maximum of 1 occurrence. Cysteine was either unmodified or labeled with a variable modification of +57.02146 Da (up to 3 occurrences). All other modifications used in the experiment are listed in Table 5. Fixed modifications were not applied except in specific runs evaluating cysteine, where carbamidomethylation (+57.02146 Da) was fixed on C.

Crystal-C, PTMProphet, and TMT-Integrator were disabled. PeptideProphet was also disabled. ProteinProphet was enabled and run with the following setting: --maxppmdiff 2000000. Percolator was used for peptide-spectrum match (PSM) validation with the following command-line options: --only-psms --no-terminate --post-processing-tdc, and a minimum probability threshold of 0.5.

MS1-based quantification was enabled using IonQuant with the following parameters: m/z tolerance of 10 ppm, retention time (RT) tolerance of 0.4 min, ion mobility tolerance 0.05 (although ion mobility was not used), labeling mode enabled with two arginine labels (+695.2942 Da and +701.3014 Da), re-quantification enabled, minimum frequency of 0.5, a minimum of 2 isotopes and 1 scan per feature, top 3 ions used for quantification, and no normalization applied. Match-between-runs was disabled. Label-free quantification was not used.

MSBooster, PTMShepherd, SAINTexpress, Skyline, and Spectral Library generation were disabled. Report generation was enabled with the following options: --sequential --razor --mapmods --prot 0.01, with decoys and known contaminants removed. Summary tables were generated at both peptide and protein levels.

Conditional Enrichment of Probe Modification by Amino Acid Raw MS data were searched using a closed-search strategy in MSFragger, and peptide-spectrum matches (PSMs) were filtered to a 1% false discovery rate (FDR). The resulting PSM table was used as input for conditional enrichment analysis. Probe-modified PSMs were identified by the presence of the expected mass-shift corresponding to the probe adduct for heavy-isoDTB, 1mM treatments in RAP experiments. Results were calculated according to the following formula:

$$Enrichment_R = \left(\frac{p(Mod \mid HasResidue)}{p(Mod \mid NoResidue)} \right)$$

Analyses were performed at the PSM level without collapsing to unique peptides, enabling assessment of residue-dependent trends in probe reactivity across the full dataset. For N-terminal proteins, PSMs were filtered as starting at protein position 1 or 2.

SASA Analysis Pipeline Output modified peptides from Fragpipe were processed using a custom Python-based pipeline to identify modified residues based on their sequence position in UniProt. Protein identifiers were initially provided as UniProt names, which were converted to their corresponding UniProt accession numbers using the UniProt API (https://rest.uniprot.org/). Structural models were then downloaded from the European Bioinformatics Institute (EBI) AlphaFold Database (https://alphafold.ebi.ac.uk/). Each protein structure was parsed using Bio.PDB (Biopython) and the Shrake-Rupley algorithm[4] was applied to compute solvent-accessible surface area (SASA) at the atomic level. Residue positions were adjusted by shifting input values down by one to ensure alignment with AlphaFold numbering. To ensure the reliability of structural predictions, residues with a predicted local distance difference test (pLDDT) score below 70 were excluded from the analysis. The relative solvent accessibility (RSA) was then calculated by normalizing SASA values against a reference maximum exposure of 274 $Å^2$. Computational storage was optimized by removing downloaded PDB files immediately following analysis, and results were iteratively written to an output file. This automated workflow enabled high-throughput extraction of solvent accessibility data from AlphaFold models, facilitating the structural interpretation of specific residues within predicted protein conformations.

For experimental structures, PDB accessions were retrieved from UniProt cross-references and corresponding mmCIF files were downloaded using Biopython's MMCIFParser. Structures with ribosomal content or more than 10 chains were excluded. Each PDB structure was aligned to the UniProt sequence to correctly map the modified residue to the structural coordinates. SASA values were computed using the same Shrake-Rupley algorithm, and RSA values were calculated using the same normalization scheme. In cases where multiple structures existed for a given protein, all qualifying entries were processed independently, and the mean and standard deviation of RSA values for each residue were calculated and used in downstream analysis.

PDB Proximal Ligand Search Pipeline To assess the structural context of modified arginine residues in proteins, an in-house computational pipeline was developed to map modified arginines from the Fragpipe output onto full-length UniProt sequences, align them to resolved PDB structures, and evaluate their spatial proximity to bound ligands. The input dataset consisted of a CSV file containing UniProt accession numbers and peptide sequences with modified arginine

residues denoted as R[701.3014]. Other modifications annotated in brackets, such as oxidation or carbamidomethylation, were ignored. The modified residue's position within the peptide was extracted, and all bracketed modifications were removed to generate a cleaned peptide sequence for further processing. To determine the position of the modified arginine in the full-length protein sequence, the cleaned peptide was aligned to the UniProt sequence using Biopython's PairwiseAligner with the BLOSUM62 substitution matrix, and affine gap penalties (-10 for opening, -0.5 for extension). The modified residue's position in the peptide was mapped to its corresponding position in the aligned UniProt sequence, yielding the residue number in the full-length protein. The script then queried the UniProt cross-references to retrieve all associated PDB accession numbers for each protein. Structures were downloaded in the PDBx/mmCIF format using MMCIFParser from Biopython. To improve computational efficiency and ensure relevance to the analysis, structures containing ribosomal components were excluded, and entries with more than 10 chains were filtered out. For structural mapping, the UniProt sequence was aligned to the sequences of all polypeptide chains in each PDB structure, allowing for potential discrepancies in residue numbering between sequence databases and structural models. The modified residue's position in the PDB structure was identified based on this alignment, ensuring that subsequent analyses were performed on the correct structural residue. To assess the molecular environment of modified arginines, the spatial relationship between the CZ atom of the modified residue and bound ligands was analyzed. NeighborSearch from Biopython[5] was used to determine whether any ligand atoms were within 6.0 Å of the arginine CZ atom. Water molecules (residue name 'HOH') were excluded to focus the analysis on small molecules and cofactors. If a ligand met the distance threshold, its residue name and chain position were recorded. To ensure robustness in handling large datasets, results were written incrementally to a CSV file containing the UniProt ID, PDB ID, chain identifier, mapped residue position in the PDB, and the nearest ligand within the specified distance.

AlphaMissense Pathogenicity Annotation To evaluate the potential functional relevance of modified arginines, AlphaMissense[6] pathogenicity scores were incorporated into the dataset. UniProt accession numbers and residue positions for all modified arginines were used to generate a queryable index. A custom Python script was developed to query the AlphaMissense API (https://alphamissense.hegelab.org/hotspotapi) for each protein and residue pair using concurrent requests with up to 10 worker threads to accelerate the process. Isoform-specific identifiers (e.g., P12345-2) were excluded to ensure consistency with canonical AlphaMissense annotations. For each query, the API returned a predicted mean pathogenicity score and a classification (e.g., 'likely pathogenic', 'likely benign'). If a classification was not explicitly returned, a custom threshold-based scheme was applied: scores ≤ 0.34 were labeled 'likely benign', scores ≥ 0.564 were labeled 'likely pathogenic', and intermediate values were designated 'ambiguous'. All results were written to a CSV file including the protein ID, residue position, score, and classification.

Secondary Structure Assignment via DSSP To assess local structural context of modified residues, DSSP-based secondary structure annotations were computed for both AlphaFold-predicted and experimentally resolved PDB structures. For AlphaFold models, UniProt IDs were first mapped to primary accessions using the UniProt API. Structural models were downloaded from the AlphaFold Protein Structure Database. For each protein, the corresponding PDB file was

parsed using Biopython's PDBParser, and DSSP secondary structure assignments were computed using Bio.PDB.DSSP. Modified residue positions were matched to AlphaFold residue indices (adjusted for 0-based numbering), and residues with a predicted local distance difference test (pLDDT) score below 75 were excluded. For residues passing the pLDDT filter, a ±4 residue sliding window was used to collect local secondary structure context. Both the DSSP code for the modified residue and its surrounding window were recorded.

For experimental structures, UniProt cross-references were used to identify relevant PDB accessions. Structures were downloaded in mmCIF format and parsed using Biopython's MMCIFParser. Resolution and structural complexity filters were applied: only structures with ≤10 chains and ≤3.0 Å resolution were retained. The best-aligned chain was selected by global pairwise sequence alignment using Biopython's PairwiseAligner, and modified residues were mapped from UniProt positions to structure-specific indices. DSSP was run on each selected PDB model to obtain secondary structure codes. For each modified residue, the DSSP code and ±3 residue sequence context were recorded. Results were aggregated across all qualifying structures for each protein, and the most frequently observed DSSP code (majority vote) was assigned as the representative secondary structure for that residue. Confidence scores were calculated as the frequency of the major assignment relative to total observations.

Motif Enrichment Analysis via pLogo To investigate sequence preferences surrounding modified arginines, motif enrichment analysis was performed using pLogo[7] (https://plogo.uconn.edu). Foreground sequences were generated from a list of experimentally identified arginine modification sites, with UniProt accession numbers and residue positions extracted from FragPipe output. A custom Python script queried the UniProt API to retrieve the full protein sequences and extracted a 21-residue window (±10 amino acids) centered on each modified arginine. If a full window could not be extracted due to proximity to sequence termini, the missing positions were padded with 'X' characters to maintain fixed length. Background sequences were generated separately from the full UniProt human proteome FASTA (including isoforms). For each protein sequence, a sliding window approach was used to extract 21-residue sequences centered on every amino acid position, again padding with 'X' as needed. This resulted in an unfiltered and comprehensive background set. Due to file size limitations on the pLogo web interface, the background set was downsampled to 1,000,000 sequences using Python's random.sample() method. Foreground and background sets were submitted to the pLogo web interface, specifying arginine (R) as the central anchor residue. A binomial statistical model with Bonferroni correction was used to evaluate motif significance. Enrichment scores and residue logos were downloaded and replotted using custom scripts to standardize styling across figures.

Salt Bridge Identification in AlphaFold Structures To identify potential salt bridge interactions between modified arginines and acidic residues, AlphaFold-predicted structural models were analyzed using a custom Python pipeline. UniProt IDs and modified residue positions were extracted from a CSV input file. Isoform identifiers were excluded. For each canonical UniProt ID, the AlphaFold API was queried to retrieve the corresponding structure metadata and download the associated PDB file. Models were stored locally and parsed using Biopython's PDBParser. Each arginine residue was scanned for proximity to nearby aspartate (ASP) or glutamate (GLU) side chain atoms (OD1/OD2 or OE1/OE2, respectively). Only models with pLDDT scores ≥ 70 for

both arginine and acidic residues were considered. Salt bridges were defined by the presence of at least one NE, NH1, or NH2 atom from the modified arginine within 4.0 Å of the target carboxylate atom. When multiple atoms satisfied the distance criterion, the closest atom pair was selected. Electrostatic interaction energies were estimated using a simplified Coulombic potential: , assuming unit charges, a dielectric constant of 20.0, and interatomic distance in Å. For each interaction, the chain ID, residue names and numbers, interacting atoms, interatomic distance, pLDDT scores, and computed Coulombic energy were recorded. Following analysis, downloaded PDB files were removed to conserve disk space. Final results were compiled into a CSV file for downstream analysis and visualization.

Cation–π Interaction Identification in AlphaFold Structures To identify potential cation–π interactions involving modified arginines, AlphaFold-predicted structures were evaluated using a geometry-based Python workflow. For each UniProt ID and arginine position, the corresponding AlphaFold model was downloaded from the EBI database. PDB files were parsed with Biopython's PDBParser, and residues were scanned for interactions between arginine CZ atoms and aromatic residues (PHE, TYR, TRP, HIS). For each aromatic residue, centroid coordinates were computed from annotated ring atoms (e.g., CZ, CE1, CE2, etc.), and the Euclidean distance to the arginine CZ atom was calculated. Interactions within 6.0 Å were considered candidates. If angular filtering was enabled, the orientation between the CZ–centroid vector and the ring plane normal was used to classify geometry as parallel (face-to-face) or T-shaped (edge-to-face), with thresholds of ≤30° and 90°±20°, respectively. Models with pLDDT scores below 70 for either the arginine or aromatic residue were excluded. For each identified interaction, the protein ID, chain IDs, residue positions and types, distance, angle, geometry classification, and pLDDT scores were recorded. Only one interaction was logged per arginine to avoid multiple counting. Results were written to two CSV outputs: a detailed interaction table and a simplified summary indicating whether each modified arginine participates in a cation–π interaction.

Salt Bridge and Cation–π Interaction Analysis in Experimental PDB Structures To validate and expand upon AlphaFold-based interaction predictions, salt bridges and cation–π interactions were also identified using experimentally resolved PDB structures. For each UniProt ID and modified residue position, all associated PDB structures were retrieved via UniProt cross-references. Structures were excluded if they represented ribosomal proteins, large macromolecular complexes (taxonomy count > 15), NMR structures, or models containing more than 10 chains. PDBx/mmCIF files were downloaded and parsed using Biopython's MMCIFParser. For each candidate arginine, neighboring residues were identified using NeighborSearch, and interactions were classified as salt bridges or cation–π based on geometric and electrostatic criteria. Salt bridges were defined as any acidic residue (ASP, GLU) within 4.0 Å of NE, NH1, or NH2 atoms of the arginine. Cation–π interactions were defined as cases where the arginine CZ atom was within 8.0 Å of the centroid of an aromatic side chain (PHE, TYR, TRP, HIS). When enabled, angular filtering was used to categorize interaction geometries as face-to-face (≤30°) or T-shaped (90° ± 20°). All interactions were annotated with residue identity, chain location, distance, geometry (if applicable), whether the partner residue was part of the same polypeptide chain, and optionally, an estimated electrostatic energy. Final results were compiled into a single CSV table containing all identified intermolecular interactions from experimental PDB models.

pKa Estimation for Arginines Using Experimental PDB Structures To estimate side-chain protonation states of modified arginines, PROPKA 3.0 was used to calculate predicted pKa values from experimental structures in the Protein Data Bank (PDB). Modified arginines were identified by UniProt accession and residue position. Isoforms and ribosomal proteins were excluded. Associated PDB structures were retrieved via UniProt cross-references and filtered to exclude NMR structures, entries with >10 polymer entities, or structures with >10 chains. Structures were downloaded in mmCIF format and parsed using Biopython's MMCIFParser. Each structure was aligned to the canonical UniProt sequence using PairwiseAligner to identify the best-matching chain and map UniProt residue indices to structural coordinates. Only arginines with a resolved CZ atom and a B-factor ≥ 30.0 were considered. Structures were converted to PDB format using Biopython's PDBIO to ensure compatibility with PROPKA. The PROPKA executable was run via subprocess call, and predicted pKa values were parsed from the resulting .pka files. For each modified arginine, the output included UniProt ID, PDB ID, chain ID, UniProt and author residue numbers, and pKa value. Structures and temporary output files were deleted after use to conserve disk space.

pKa Estimation for Arginine, Lysine or Cysteine from AlphaFold Models To complement structure-based pKa estimation, PROPKA was also applied to AlphaFold-predicted structures. AlphaFold models were downloaded in PDB format for each UniProt accession. Only residues with pLDDT confidence scores >70 were considered for analysis. For each AlphaFold structure, PROPKA 3.0[8] was executed using the PDB file as input. Predicted pKa values were extracted using a custom parser that accommodated multiple possible output formats. Residue-level pKa values were reported for each modified arginine, cysteine or lysine, alongside the corresponding pLDDT score. Proteins for which PROPKA failed to produce valid output were excluded. Temporary AlphaFold PDB and PROPKA output files were deleted after processing.

Intrinsic Disorder Prediction via IUPred2A To evaluate the intrinsic disorder propensity of modified arginine residues, disorder scores were retrieved using the IUPred2A web API (https://iupred2a.elte.hu). A CSV file containing UniProt IDs and modified residue positions served as input. For each protein, the full-length disorder profile was fetched in JSON format and parsed to extract per-residue IUPred2 scores. Modified arginine positions were indexed using 1-based UniProt residue numbering. Prior to extraction, each index was converted to 0-based to match Python list conventions. If a position fell within the length of the retrieved disorder profile, the corresponding IUPred2 score was recorded. Otherwise, 'NA' was assigned. Each entry in the output file included the UniProt ID, residue position, and corresponding disorder score. Results were written iteratively to a CSV file for downstream integration with structural and reactivity data.

Molecular Docking and Covalent Adduct Modeling Molecular docking was performed using the Molecular Operating Environment (MOE2024[9]) software. The crystal structures of aconitase (PDB: 1B0J) and cyclophilin A (PDB: 6GJI) were obtained, with all crystallographic ions removed. Each structure was solvated in a 10 Å water sphere to mimic physiological conditions before preparation using MOE's QuickPrep feature, which optimized hydrogen placement, assigned protonation states, and performed an initial energy minimization. For aconitase, the docking site was defined based on the known binding site of isocitrate, and the dehydrate of ninhydrin was docked using MOE's docking protocol. For cyclophilin A, the ligand **CA-Nin-Alk** was docked using

MOE's template-based docking to ensure proper positioning. In both cases, using the triangle matcher placement/rigid receptor refinement method, returning 1000 poses and retaining the top 30 poses based on GBVI/WSA dG scoring function, the top-scoring poses were selected and loaded into the MOE Window, where a covalent adduct was manually constructed using the builder module by building bonds from the proximal face of the ninhydrin to the nearest guanidinium nitrogens of the target arginine residue. The resulting adduct geometry was refined by energy minimization performed using the Amber10:EHT force field with gradient-based optimization until the root mean square gradient reached 0.05 kcal/(mol·Å) allowing bond lengths and angles to relax to chemically reasonable values. Visualization and figure preparation were performed using ChimeraX 1.6.1[10].

Cyclophilin A Inhibition Assay To measure **CA-Nin-Alk** inhibition of cyclophilin A (CypA) activity, a peptidyl-proline isomerase (PPIase) assay was utilized following a previously established protocol[11]. Recombinant human CypA (R&D Systems) was diluted to a concentration of 1 μM in a reaction buffer of 20 mM HEPES, 100 mM NaCl, and 0.5 mM TCEP (pH 7.5) and kept on ice. Chymotrypsin was diluted to 60 mg/mL in 2 mM $CaCl_2$, 1 mM HCl in H_2O. To test inhibition, the inhibitor, **CA-Nin-Alk,** was diluted in DMSO to generate 100X stock solutions (25 μM, 10 μM). These were added to CypA solutions (final DMSO = 1% v/v) and incubated for 1 hour at room temperature. The substrate, suc-AAPF-pNA (Thomas Scientific), was freshly prepared at 10 mM in 470 mM LiCl/TFE. The spectrophotometer (Cary UV-Vis Spectrophotometer) was cooled to 4°C before use. For uncatalyzed reactions, 980 μL of reaction buffer and 10 μL of chymotrypsin were mixed in a cuvette and placed in the spectrophotometer. The reaction was initiated with 10 μL substrate and A390 was monitored every 0.1s for 5 minutes. For CypA catalyzed reactions, 970 μL of the reaction buffer, 10 μL of chymotrypsin, and 10 μL of CypA solution (pre-incubated ± inhibitor) were combined prior to substrate addition and identical data collection. All assays were performed in triplicate. To ensure calculations depended solely on the first-order reaction of isomerization and not residual trans-substrate in the solution, the absorbance traces, excluding the first 10s, were fit to a single-exponential function to obtain the rate constants for uncatalyzed (k_{uncat}) or CypA-catalyzed (k_{obs}) isomerization. k_{cat}/K_m values were then calculated via the following equation:

$$\frac{k_{cat}}{K_M} = \frac{k_{obs} - k_{uncat}}{[CypA]}$$

using a final enzyme concentration of $1*10^{-8}$M.

IC_{50} calculations Apparent IC_{50} values were estimated using a two-point linear interpolation. Percent inhibition of CypA catalytic efficiency (k_{cat}/K_m) was measured at two compound concentrations (25 μM, 10 μM). A linear regression of inhibition (y) vs compound concentration (x) was used to approximate the relationship ($y = mx + b$), and the concentration corresponding to 50% inhibition was calculated as:

$$IC_{50} = \frac{(50 - b)}{m}$$

Code Availability Statement All code is available on the ZaroLab GitHub. Code is available for academic use, all other non-academic usage is restricted to those with permission from the corresponding author.

Statement of Data Availability: All raw mass spectrometry datasets are available on MASSive under the ID: MSV000100438. Other raw data and processed data essential to this work are provided in the Supplementary Information and Source Data file. Source data are provided with this paper via 10.5281/zenodo.15579687.

Methods References

(1) Hanwell, M. D.; Curtis, D. E.; Lonie, D. C.; Vandermeersch, T.; Zurek, E.; Hutchison, G. R. Avogadro: An Advanced Semantic Chemical Editor, Visualization, and Analysis Platform. *J. Cheminform.* **2012**, *4* (1), 17.
(2) Perri, M. J.; Weber, S. H. Web-Based Job Submission Interface for the GAMESS Computational Chemistry Program. *J. Chem. Educ.* **2014**, *91* (12), 2206–2208.
(3) Böhme, A.; Thaens, D.; Paschke, A.; Schüürmann, G. Kinetic Glutathione Chemoassay to Quantify Thiol Reactivity of Organic Electrophiles--Application to Alpha,beta-Unsaturated Ketones, Acrylates, and Propiolates. *Chem. Res. Toxicol.* **2009**, *22* (4), 742–750.
(4) Shrake, A.; Rupley, J. A. Environment and Exposure to Solvent of Protein Atoms. Lysozyme and Insulin. *J. Mol. Biol.* **1973**, *79* (2), 351–371.
(5) Cock, P. J. A.; Antao, T.; Chang, J. T.; Chapman, B. A.; Cox, C. J.; Dalke, A.; Friedberg, I.; Hamelryck, T.; Kauff, F.; Wilczynski, B.; de Hoon, M. J. L. Biopython: Freely Available Python Tools for Computational Molecular Biology and Bioinformatics. *Bioinformatics* **2009**, *25* (11), 1422–1423.
(6) Cheng, J.; Novati, G.; Pan, J.; Bycroft, C.; Žemgulytė, A.; Applebaum, T.; Pritzel, A.; Wong, L. H.; Zielinski, M.; Sargeant, T.; Schneider, R. G.; Senior, A. W.; Jumper, J.; Hassabis, D.; Kohli, P.; Avsec, Ž. Accurate Proteome-Wide Missense Variant Effect Prediction with AlphaMissense. *Science* **2023**, *381* (6664), eadg7492.
(7) O'Shea, J. P.; Chou, M. F.; Quader, S. A.; Ryan, J. K.; Church, G. M.; Schwartz, D. pLogo: A Probabilistic Approach to Visualizing Sequence Motifs. *Nat. Methods* **2013**, *10* (12), 1211–1212.
(8) Olsson, M. H. M.; Søndergaard, C. R.; Rostkowski, M.; Jensen, J. H. PROPKA3: Consistent Treatment of Internal and Surface Residues in Empirical pKa Predictions. *J. Chem. Theory Comput.* **2011**, *7* (2), 525–537.
(9) Molecular Operating Environment (MOE), 2024.0601 Chemical Computing Group ULC. **2026**, 910–1010.
(10) Meng, E. C.; Goddard, T. D.; Pettersen, E. F.; Couch, G. S.; Pearson, Z. J.; Morris, J. H.; Ferrin, T. E. UCSF ChimeraX: Tools for Structure Building and Analysis. *Protein Sci.* **2023**, *32* (11), e4792.
(11) Kofron, J. L.; Kuzmic, P.; Kishore, V.; Colón-Bonilla, E.; Rich, D. H. Determination of Kinetic Constants for Peptidyl Prolyl Cis-Trans Isomerases by an Improved Spectrophotometric Assay. *Biochemistry* **1991**, *30* (25), 6127–6134.

Uncropped Gels

Uncropped gel images from Figure 2B.

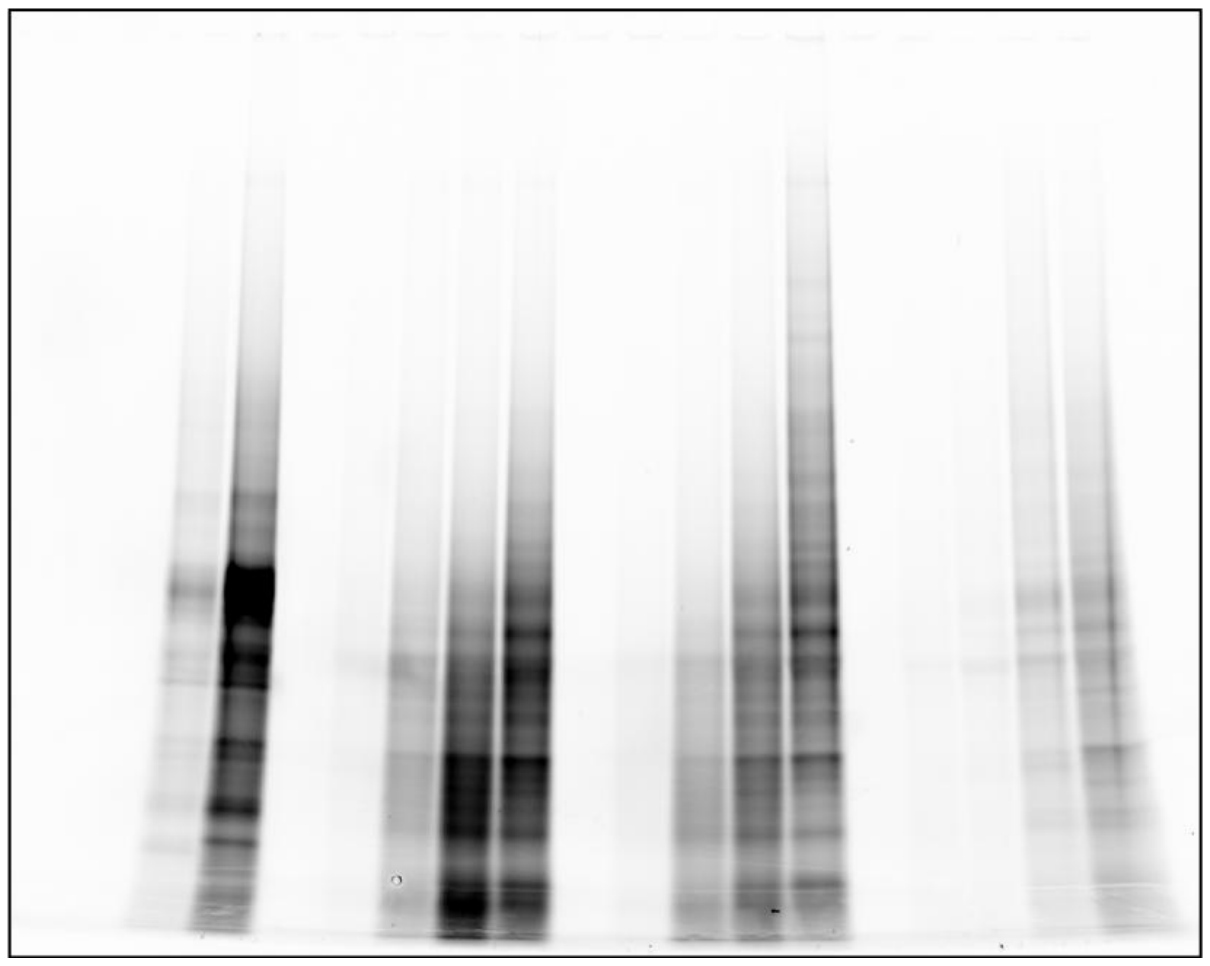

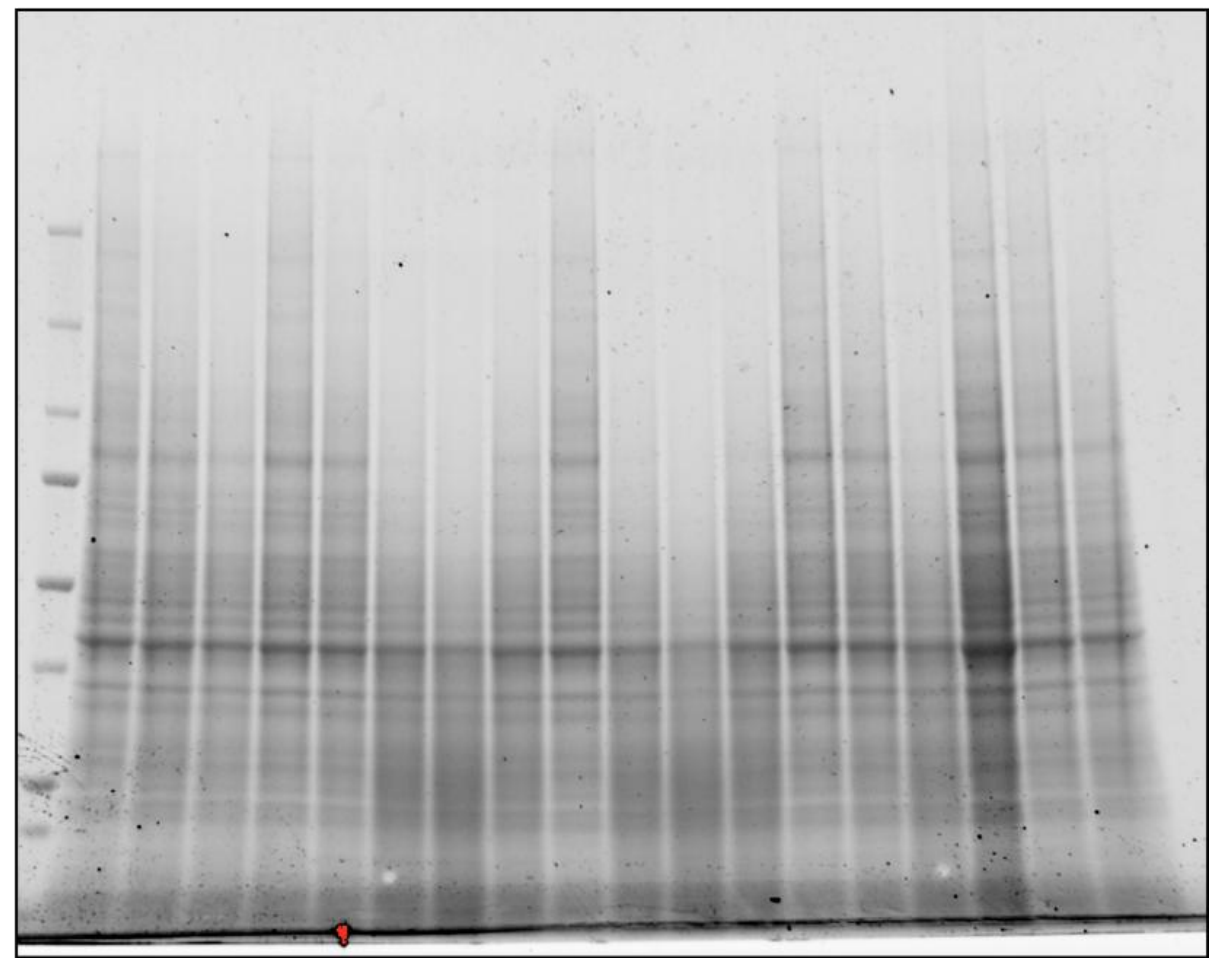

Qualitative, gel-based assessment of proteome-wide reactivity of alkyne-bearing ninhydrin probes in Mino cell lysate. Soluble proteome (1.5 μg μL^{-1}) was treated with each probe at increasing concentrations (1, 10, or 100 μM) for 1 h, followed by CuAAC conjugation with TAMRA-N_3, separation by SDS–PAGE, and in-gel fluorescence scanning.

Uncropped gel images from Figure 2C.

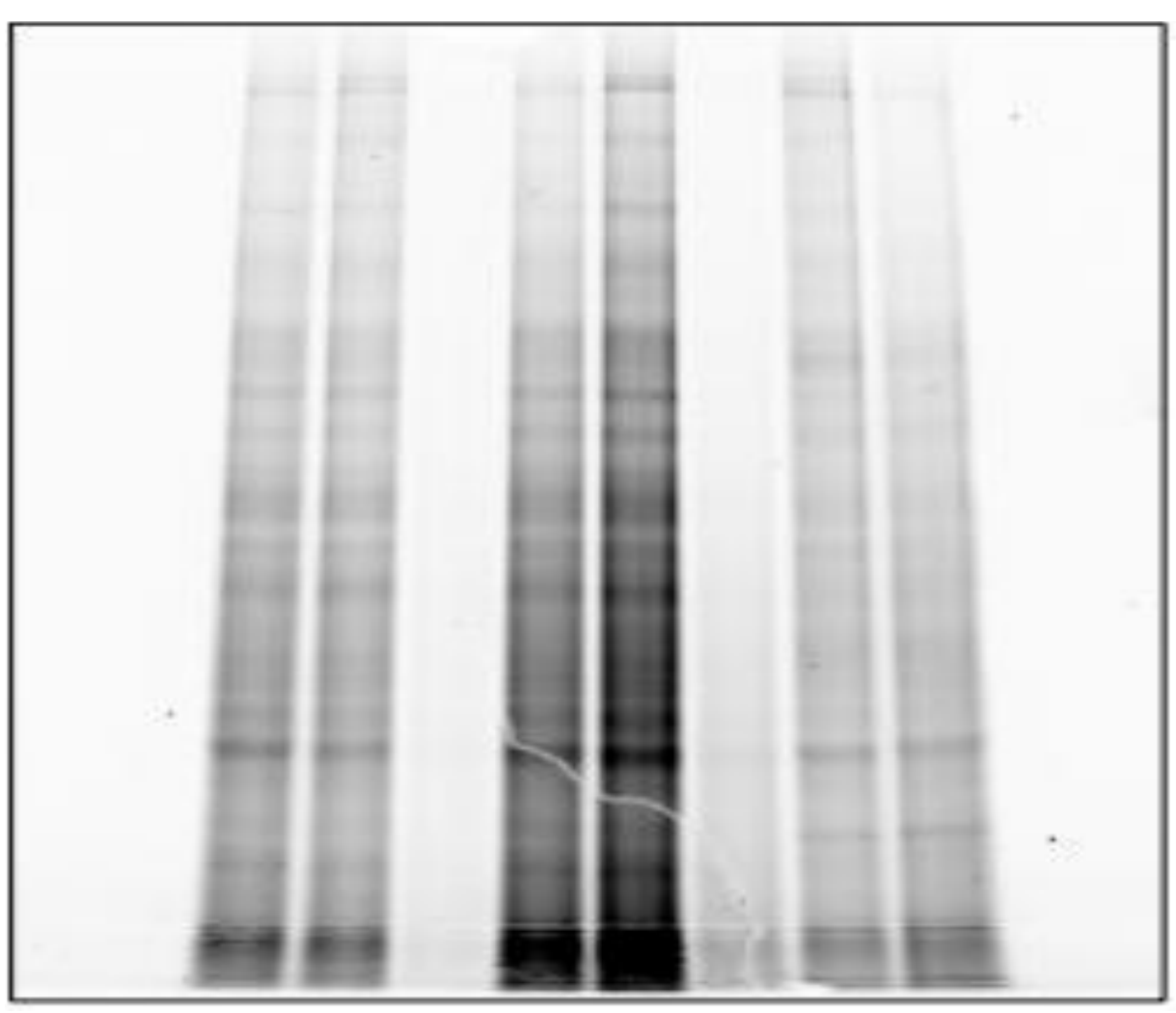

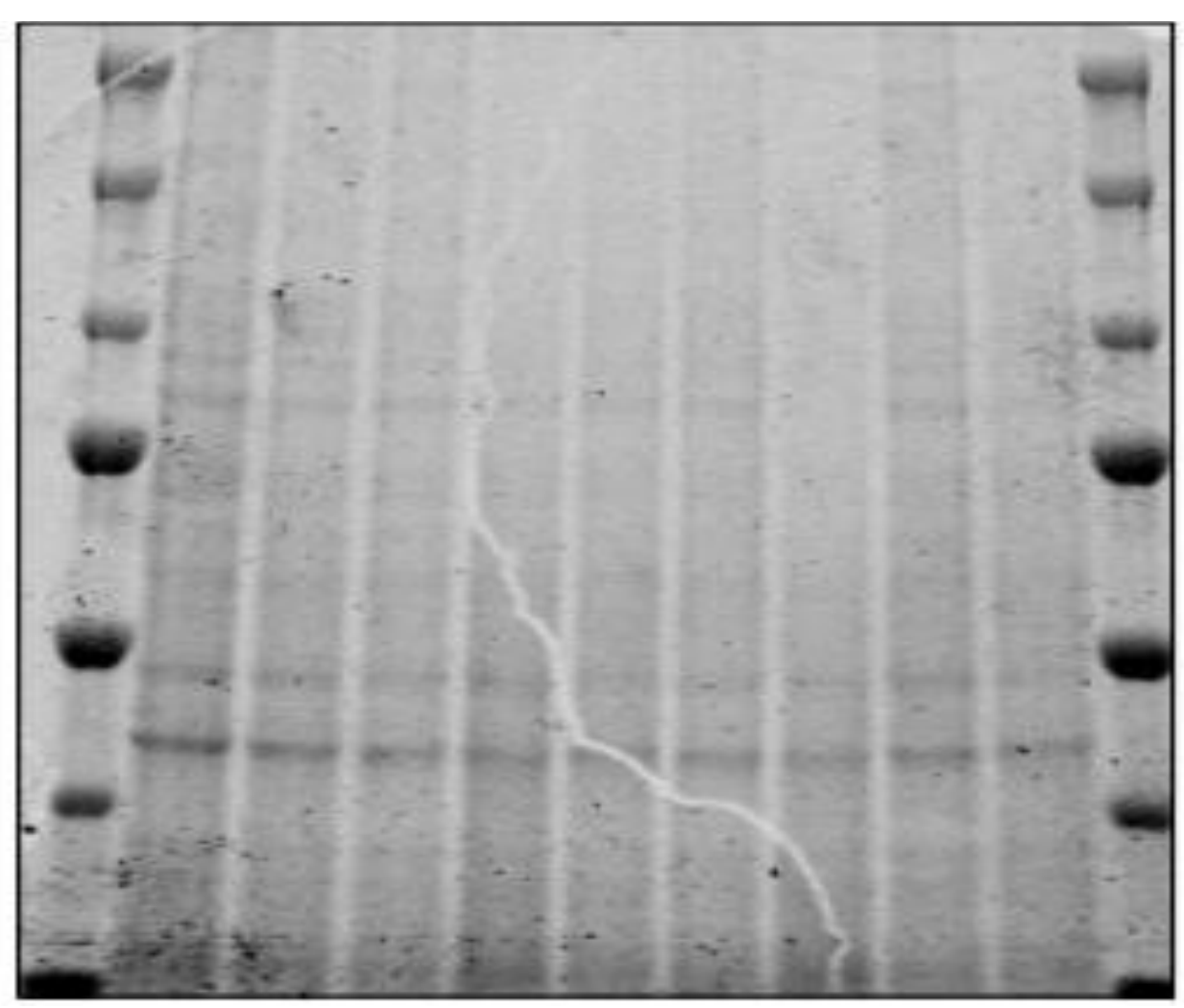

Qualitative time-dependent reactivity of 4-Nin-Alk, Nin-Alk, and PGO in live cells. Mino cells were treated with 100 μM of indicated probe for 0, 30, or 60 min followed by lysis, CuAAC conjugation with TAMRA-N_3, separation by SDS–PAGE, and in-gel fluorescence scanning.

Representative Traces

Representative Fmoc-AA-OH consumption assay HPLC traces.

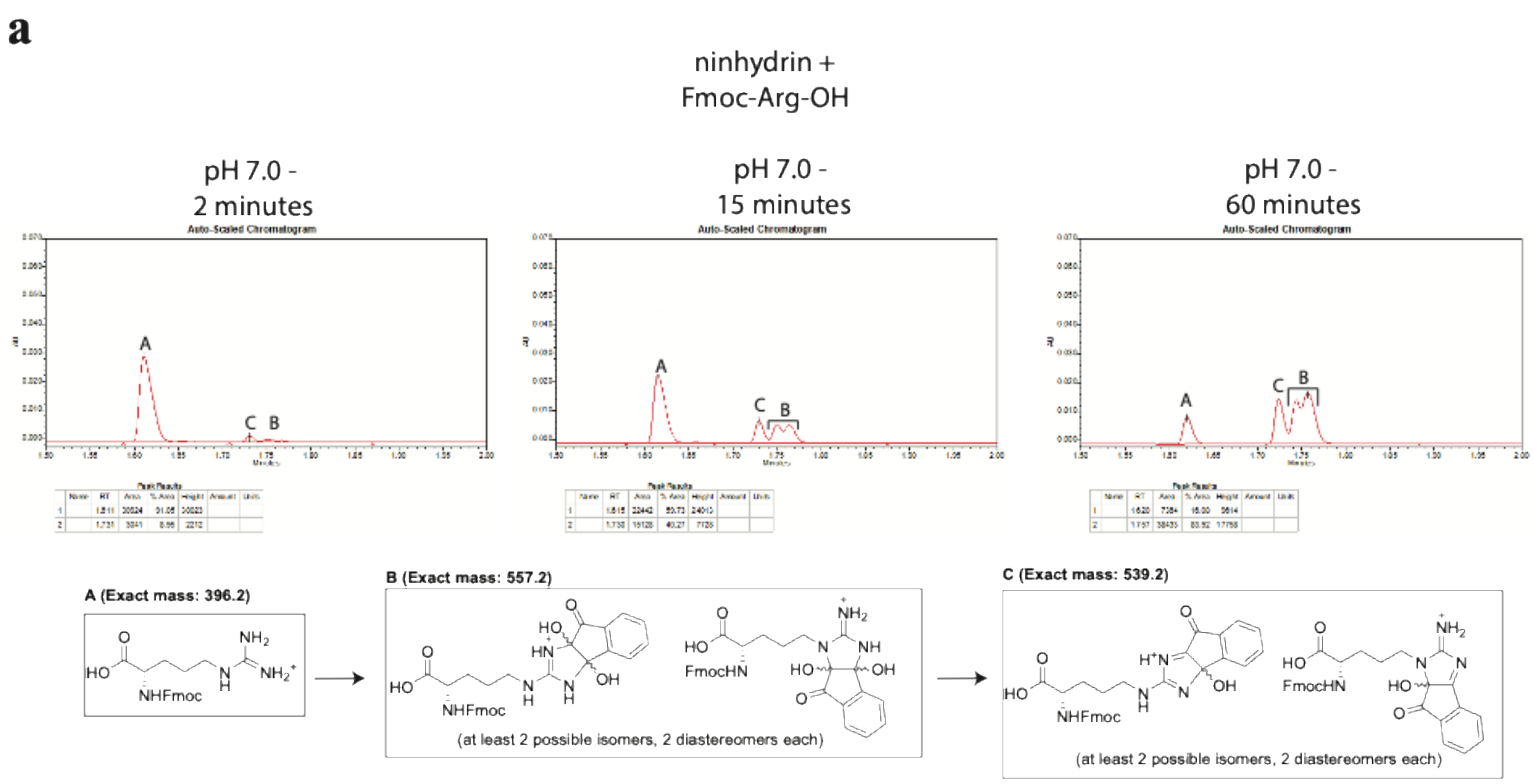

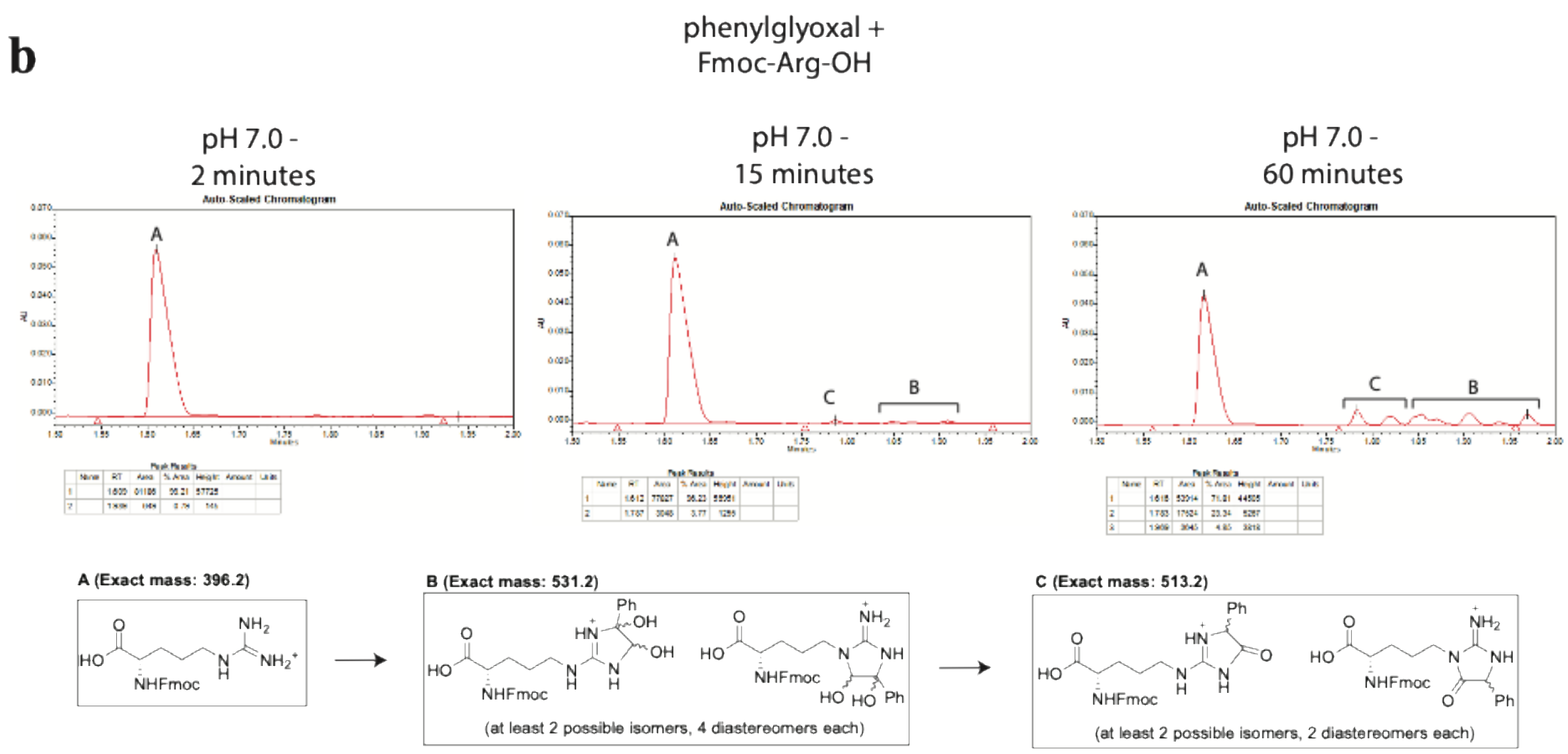

Representative HPLC traces for Fmoc-AA-OH consumption assay in Figure 1B, Figure 3C, Extended Data Figure 1A and Extended Data 3A. Kinetic analysis of pH- and time-dependent consumption of Fmoc-Arg by ninhydrin (**a**) or PGO (**b**) (15 mM, 30x) in pH 7.0 PBS at 20 °C. Fmoc-AA consumption was quantified by LC-MS HPLC traces at 300nm wavelength for samples quenched between 2 to 60 minutes. Each trace represents an individual replicate. Modified products are annotated by peak labels corresponding to exact mass species drawn explicitly below traces.

Representative Intact Protein Mass Spectrometry Deconvoluted Spectra.

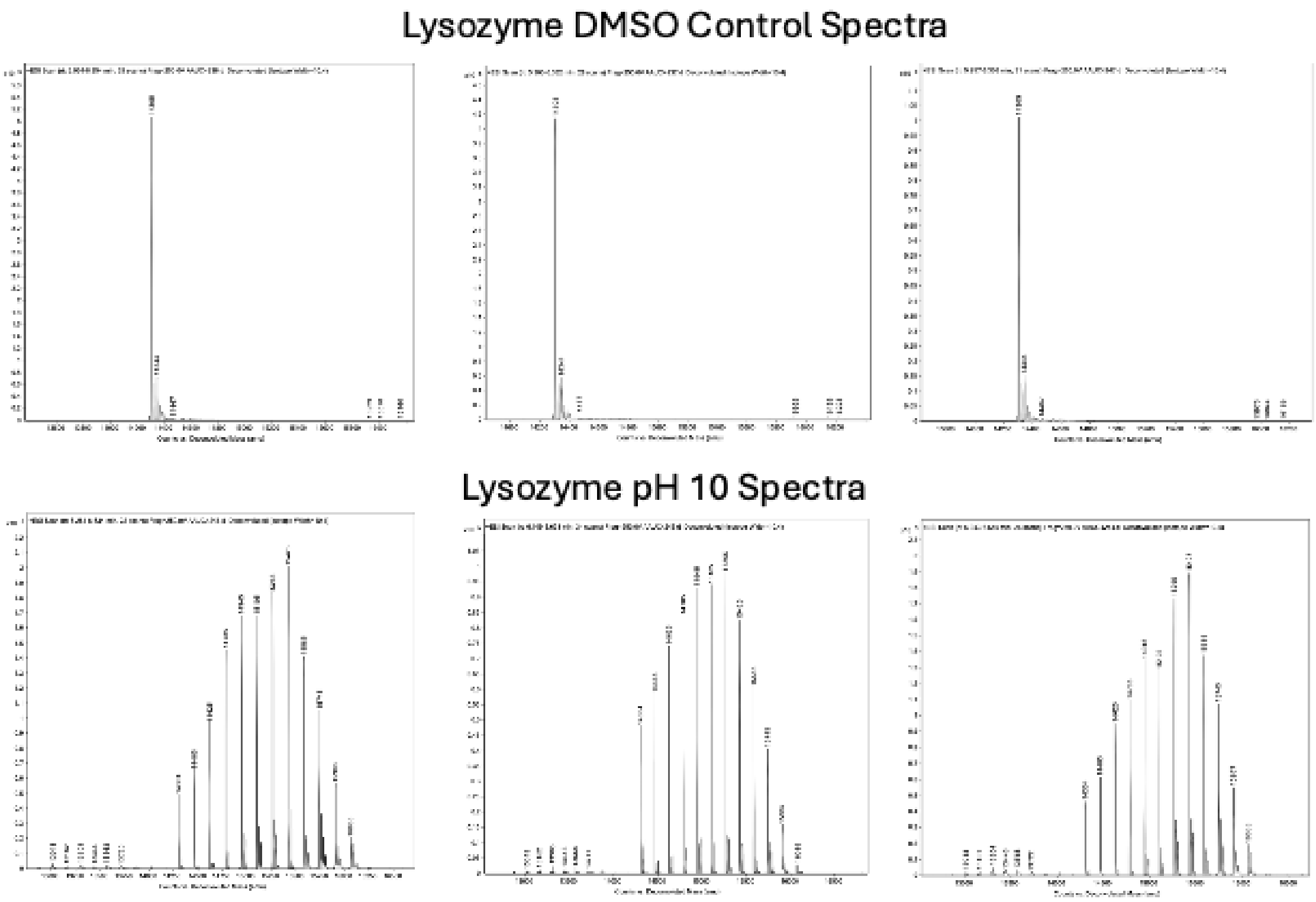

Representative intact protein mass spectrometry deconvoluted mass spectra as acquired for Figures 1D and Extended Data Figure 3. Intact protein mass spectrometry analysis of Lysozyme (1.5 µM) and 100 µM ninhydrin at 37 °C for 30 minutes across pH 4.0, 7.0 and 10.0. Reactions were quenched by dilution into 0.1% aqueous TFA and analyzed by LC–MS on an Agilent 6230 TOF system. Each spectra represents an individual replicate

Synthesis of Probes

General Experimental Procedures: All reactions were performed in flame- or oven-dried glassware fitted with rubber septa under a positive pressure of nitrogen or argon, unless otherwise noted. All reaction mixtures were stirred throughout the course of each procedure using Teflon-coated magnetic stir bars. Air- and moisture-sensitive liquids were transferred via syringe or stainless-steel cannula. Solutions were concentrated by rotary evaporation below 35 °C. Analytical thin-layer chromatography (TLC) was performed using glass plates pre-coated with silica gel (0.25-mm, 60-Å pore size, 230−400 mesh, SILICYCLE INC) impregnated with a fluorescent indicator (254 nm). TLC plates were visualized by exposure to ultraviolet light (UV), and then were stained by submersion in a basic aqueous solution of potassium permanganate or with an acidic ethanolic solution of ninhydrin, followed by brief heating.

Materials: Dichloromethane (DCM), dimethylformamide (DMF), tetrahydrofuran (THF), ethyl ether, and acetonitrile to be used in anhydrous reaction mixtures were dried by passage through activated alumina columns immediately prior to use. Hexanes used were ≥85% n-hexane. Other commercial solvents and reagents were used as received, unless otherwise noted.

Instrumentation: Unless otherwise noted, proton nuclear magnetic resonance (^{1}H NMR) spectra and carbon nuclear magnetic resonance (^{13}C NMR) spectra were recorded on a JEOL JNM-ECZ400R 400 MHz NMR equipped with 5 mm H/F/X Royal Probe or a Bruker AVIII HD 600 MHz NMR equipped with 5 mm CPDCH CryoProbe at 23 °C. Proton chemical shifts are expressed in parts per million (ppm, δ scale) and are referenced to residual protium in the NMR solvent ($CDCl_3$: δ 7.26, CD_3CN: δ 1.94). Carbon chemical shifts are expressed in parts per million (ppm, δ scale) and are referenced to the carbon resonance of the NMR solvent ($CDCl_3$: δ 77.16, CD_3CN: δ 118.26). Data are represented as follows: chemical shift, multiplicity (s = singlet, d = doublet, t = triplet, q = quartet, dd = doublet of doublets, dt = doublet of triplets, sxt = sextet, m = multiplet, br = broad, app = apparent), integration, and coupling constant (J) in hertz (Hz). Mass measurements for high-resolution mass spec (HRMS) were performed on a Waters Xevo G2-XS TOF calibrated against sodium formate clusters and using a LeuEnk lockmass. Expected monoisotopic masses were calculated using MassLynx 4.1 and the *m/z* values for calibrant and lockmass were MassLynx-default values.

Compound SI-1

DMF (13.5 mL) was added to a round-bottom flask containing K_2CO_3 (1.87 g, 13.50 mmol, 2.00 equiv) and 5-hydroxy-2,3-dihydro-1H-inden-1-one (1.00 g, 6.75 mmol, 1 equiv). Propargyl bromide (80 wt% in toluene, 829 µL, 1.10 g, 7.42 mmol, 1.10 equiv) was added dropwise over 1 minute. The reaction vessel was heated in a 60 °C oil bath for 16 h. After cooling to ambient temperature, the mixture was diluted with DCM (30 mL) and washed with DI water (3 × 30 mL) followed by brine (2 × 30 mL). The organic layer was dried over Na_2SO_4, filtered, and the filtrate

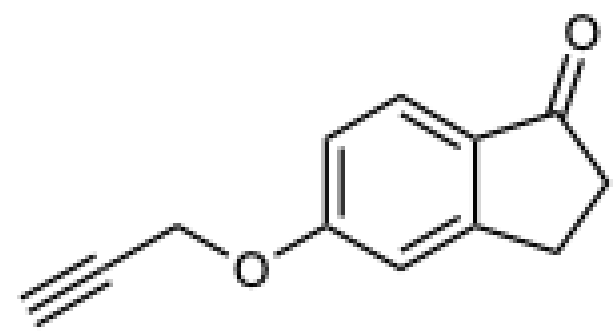

was concentrated under reduced pressure to afford **SI-1** as a brown solid, which was used in subsequent steps without further purification (1.22 g, 97% yield).

TLC (hexanes:ethyl acetate = 8:2): R_f = 0.25 (UV)

¹H NMR (400 MHz, $CDCl_3$) δ: 7.71 (d, *J* = 8.4 Hz, 1H), 7.00 (s, 1H), 6.97 (d, *J* = 8.7 Hz, 1H), 4.77 (t, *J* = 2.1 Hz, 2H), 3.11 (t, *J* = 5.4 Hz, 2H), 2.69 (t, *J* = 5.9 Hz, 2H), 2.57 (s, 1H).

¹³C NMR (101 MHz, $CDCl_3$) δ: 205.41, 163.06, 158.06, 131.22, 125.50, 115.88, 111.00, 77.74, 76.35, 56.05, 36.53, 25.99.

HRMS (ESI⁺): *m/z* calcd for $C_{12}H_{11}O_2^+$ ($[M+H]^+$): 187.0759; found: 187.0756.

Nin-Alk

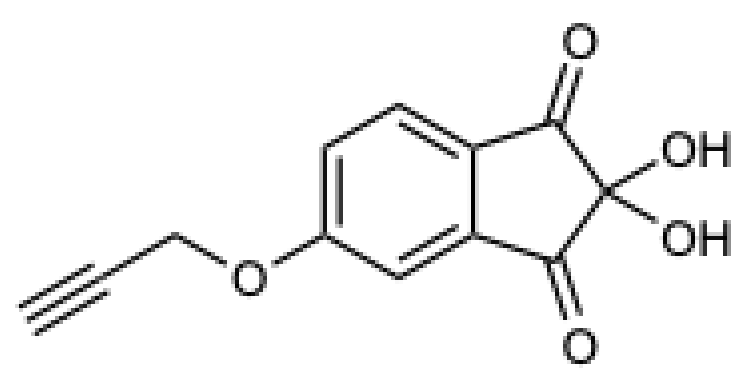

Selenium dioxide (745 mg, 6.71 mmol, 2.5 equiv) was added to a solution of **SI-1** (500 mg, 2.69 mmol, 1 equiv) in 1,4-dioxane (3.25 mL) and DI water (325 µL). The reaction mixture was heated to reflux in a 96 ºC oil bath for 4 hours. After cooling to ambient temperature, the mixture was diluted with ethyl acetate (15 mL) and washed successively with DI water (2 × 10 mL), 1 M aqueous HCl (2 × 10 mL). The organic layer was washed with brine (1 × 10 mL), dried over Na_2SO_4, filtered, and the filtrate was concentrated under reduced pressure. The crude residue was purified by flash chromatography (30–50% acetone in hexanes) to afford a red solid which was still contaminated with what may be some selenium byproducts. Final purification was performed by reverse-phase HPLC (2–25% MeCN with 0.1% TFA in milliQ water with 0.1% TFA), and lyophilization of the collected fractions yielded the product as a white powder (561 mg, 90% yield).

TLC (hexanes:acetone = 1:1): R_f = 0.34 (UV)

¹H NMR (400 MHz, 10:1 $CD_3CN + D_2O$) δ: 7.91 (d, J = 8.6 Hz, 1H), 7.47 (dd, J = 8.6, 2.5 Hz, 1H), 7.40 (d, J = 2.4 Hz, 1H), 4.88 (d, J = 2.4 Hz, 2H), 2.95 (t, J = 2.4 Hz, 1H)

¹³C NMR (151 MHz, 10:1 $CD_3CN + D_2O$) δ: 196.80, 195.28, 165.07, 142.06, 133.47, 126.88, 126.43, 107.55, 88.21, 77.88, 77.73, 57.22

HRMS (ESI⁺): *m/z* calcd for $C_{12}H_9O_5^+$ ($[M+H]^+$): 233.0450; found: 233.0448.

Compound SI-2

To a round-bottom flask containing K_2CO_3 (1.87 g, 13.50 mmol, 2.00 equiv) and 4-hydroxy-2,3-dihydro-1H-inden-1-one (1.00 g, 6.75 mmol, 1 equiv) was added anhydrous DMF (13.5 mL). Propargyl bromide (80 wt% in toluene, 829 µL, 1.10 g, 7.42 mmol, 1.10 equiv) was added dropwise over the course of 1 minute. The vessel was heated in a 60 °C oil bath for 16 h. After

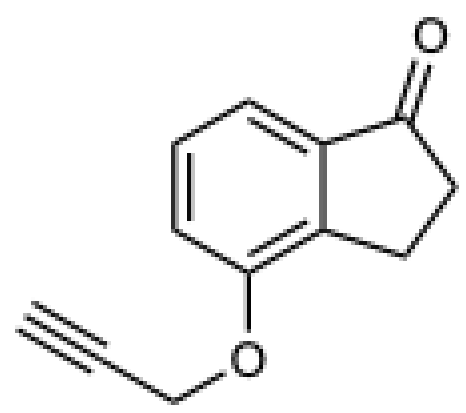

cooling to ambient temperature, the mixture was diluted with DCM (30 mL) and washed with DI water (3 × 30 mL) followed by brine (2 × 30 mL). The organic layer was dried over Na_2SO_4, filtered, and the filtrate was concentrated under reduced pressure to afford **SI-2** as a tan solid, which was used in subsequent steps without further purification (1.18 g, 94% yield).

TLC (hexanes:ethyl acetate = 8:2): R_f = 0.40 (UV)

^{1}H NMR (400 MHz, $CDCl_3$) δ: 7.40 (d, *J* = 7.6 Hz, 1H), 7.36 (t, *J* = 7.6 Hz, 1H), 7.17 (d, *J* = 7.7 Hz, 1H), 4.80 (s, 2H), 3.07 (t, *J* = 6.0 Hz, 2H), 2.69 (t, *J* = 6.0 Hz, 2H), 2.55 (s, 1H)

^{13}C NMR (100 MHz, $CDCl_3$) δ: 207.23, 155.10, 144.57, 139.04, 128.86, 116.50, 116.45, 78.18, 76.22, 56.04, 36.26, 22.70.

HRMS (ESI$^+$): *m/z* calcd for $C_{12}H_{11}O_2^+$ ([M+H]$^+$): 187.0759; found: 187.0757

4-Nin-Alk

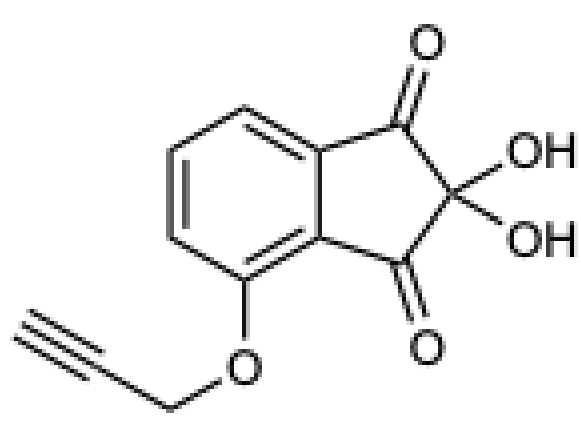

To a solution of **SI-2** (500 mg, 2.69 mmol, 1 equiv) in 1,4-dioxane (3.25 mL) and DI water (325 µL) was added selenium dioxide (745 mg, 6.71 mmol, 2.50 equiv). The reaction mixture was heated to reflux in a 96 ºC oil bath for 4 hours. After cooling to ambient temperature, the mixture was diluted with ethyl acetate (15 mL) and washed successively with DI water (2 × 10 mL) and 1 M aqueous HCl (2 × 10 mL). The organic layer was washed with brine (1 × 10 mL), dried over Na_2SO_4, filtered, and the filtrate was concentrated under reduced pressure. The crude residue was purified by flash chromatography (30–50% acetone in hexanes) to yield a red solid. Final purification was performed by reverse-phase HPLC (2–25% MeCN with 0.1% TFA in milliQ water with 0.1% TFA) to remove selenic acid byproducts yielding a white powder after lyophilization (532 mg, 2.29 mmol, 85% yield).

TLC (hexanes:acetone = 1:1): R_f = 0.36 (UV)

^{1}H NMR (400 MHz, 10:1 CD_3CN + D_2O) δ: 7.93 (dd, J = 8.3, 7.6 Hz, 1H), 7.58 (d, J = 7.0 Hz, 1H), 7.55 (d, J = 7.9 Hz, 1H), 4.96 (d, J = 2.4 Hz, 2H), 2.97 (t, J = 2.4 Hz, 1H)

^{13}C NMR (100 MHz, 10:1 CD_3CN + D_2O) δ: 197.57, 195.32, 156.97, 141.29, 140.27, 127.29, 121.95, 117.54, 88.0, 78.62, 78.24, 57.58

HRMS (ESI$^+$): *m/z* calcd for $C_{12}H_9O_5^+$ ([M+H]$^+$): 233.0450; found: 233.0446

Compound SI-3

A solution of 4-(trifluoromethyl)benzaldehyde (1.74 g, 10.0 mmol, 1 equiv), propargylamine (826 mg, 15.0 mmol, 2.50 equiv), sodium triacetoxyborohydride (5.30 g, 25.0 mmol, 2.50 equiv), and glacial acetic acid (600 mg, 10.0 mmol, 1 equiv) in DCM (95 mL) was stirred at ambient temperature for 2.5 h. The reaction mixture was diluted with DCM (40 mL) and saturated aqueous

$NaHCO_3$ (40 mL) was added, and the layers were mixed vigorously and then separated. The aqueous layer was extracted with DCM (2 × 20 mL), and the combined organic layers were concentrated under reduced pressure. The residue was dissolved in ether (50 mL) and extracted with 1.0 M aqueous HCl (3 × 30 mL). The combined aqueous extracts were basified to pH ~11–12 (as judged by pH strips) with 1.0 M aqueous NaOH and extracted with ether (4 × 20 mL). The combined organic layers were dried over Na_2SO_4, filtered, and the filtrate was concentrated. The crude residue was purified by flash chromatography (silica gel, gradient from 10-25% ethyl acetate in hexanes) to afford N-(4-(trifluoromethyl)benzyl)prop-2-yn-1-amine (2.05 g, 96% yield) as a colorless oil.

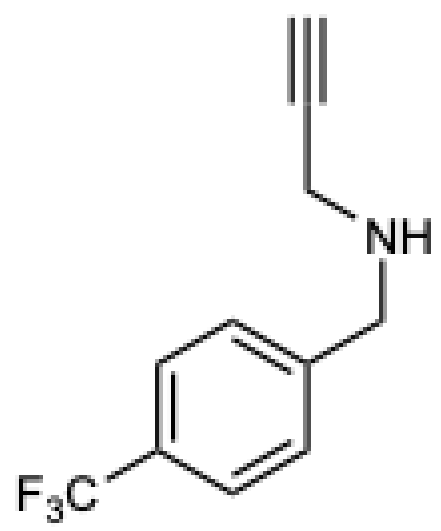

TLC (hexanes:ethyl acetate = 4:1): R_f = 0.32 (ninhydrin)
^{1}H NMR (400 MHz, $CDCl_3$) δ: 7.59 (d, J = 7.9 Hz, 2H), 7.48 (d, J = 7.9 Hz, 2H), 3.95 (s, 2H), 3.43 (s, 2H), 2.27 (s, 1H)
^{13}C NMR (101 MHz, $CDCl_3$) δ: 143.58(d, J = 32.57), 129.73, 128.67, 125.42(q, J= 3.8 Hz), 124.23 (q, J = 271 Hz) 81.78, 71.94, 51.68, 37.41
^{19}F NMR (376 MHz, $CDCl_3$) δ: -62.31
HRMS (ESI$^+$): *m/z* calcd for $C_{11}H_{11}F_3N^+$ ($[M+H]^+$): 214.0884; found: 214.0842

Compound SI-4

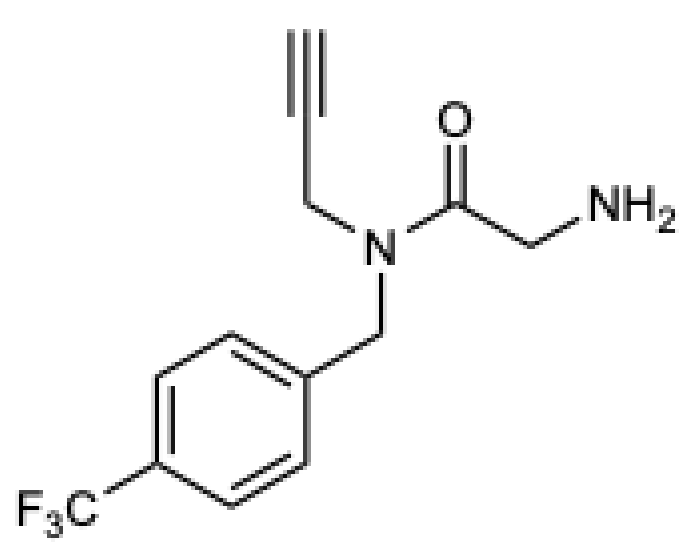

To a solution of **SI-3** (365 mg, 1.71 mmol, 1 equiv) in DMF (8.56 mL) was added DIPEA (664 mg, 895 µL, 5.14 mmol, 3.00 equiv), followed by (tert-butoxycarbonyl)glycine (330 mg, 1.88 mmol, 1.10 equiv) and PyAOP (976 mg, 2.57 mmol, 1.50 equiv). The mixture was briefly sonicated and stirred at ambient temperature for 1 hour. The reaction was diluted with ethyl acetate (50 mL) and sequentially washed with 1.0 M aqueous HCl (2 × 15 mL), DI water (2 × 15 mL), saturated aqueous $NaHCO_3$ (2 × 15 mL), and brine (2 × 15 mL). The organic layer was dried over Na_2SO_4, filtered, and the filtrate was concentrated under reduced pressure to afford the Boc-protected intermediate as a white solid. This material was then dissolved in 4 M aqueous HCl in dioxane and stirred for 3 hours at ambient temperature to yield the HCl salt of the product as a white solid after diluting in toluene and concentrating under reduced pressure. The HCl salt was freebased with 2 mL aqueous saturated $NaHCO_3$ and extracted with 20 mL ethyl acetate which was subsequently dried over Na_2SO_4, filtered, and the filtrate was concentrated under reduced pressure to yield pure **SI-4** (392.7 mg, 85% yield) as a white foam which was used in subsequent steps without further purification.

TLC (hexanes:ethyl acetate = 1:1): R_f = 0.35 (ninhydrin)

^{1}H NMR (400 MHz, $CDCl_3$) δ: 7.54 (d, *J* = 8.0 Hz, 1H), 7.49 (d, *J* = 8.0 Hz, 1H), 7.42 (d, *J* = 8.0 Hz, 1H), 7.33 (d, *J* = 8.0 Hz, 1H), 4.60 (s, 2H, Ha, major rotamer), 4.62 (s, 2H, Ha′, minor rotamer), 4.29 (s, 2H, Hb, major rotamer), 4.35 (s, 2H, Hb′, minor rotamer), 4.00 (s, 2H, Hc, major rotamer), 3.96 (s, 2H, Hc′, minor rotamer), 2.26 (s, 1H, Hd, major rotamer), 2.14 (s, 1H,

Hd′, minor rotamer), 1.91 (b, 3H, He, both rotamers overlapping). Signals marked with Ha/Ha′–Hd/Hd′ correspond to rotameric pairs arising from restricted rotation about the amide bond. Rotamers are observed in a ~55:45 ratio (major:minor).

^{13}C NMR (101 MHz, $CDCl_3$) δ: 173.25, 140.71, 139.54, 130.31, 129.97, 128.65, 127.07, 126.16(q, J = 3.8 Hz), 125.76 (q, J = 3.8 Hz), 124.14(q, J = 271 Hz),78.23, 77.35, 73.54, 72.82, 48.85, 43.51, 35.78, 35.11

^{19}F NMR (376 MHz, $CDCl_3$) δ: -62.58 (d, J = 36.0 Hz, 3F)

HRMS (ESI$^+$): *m/z* calcd for $C_{13}H_{14}F_3N_2O_3^+$ ($[M+H]^+$): 271.1058; found: 271.1050

CA-Ind-Alk

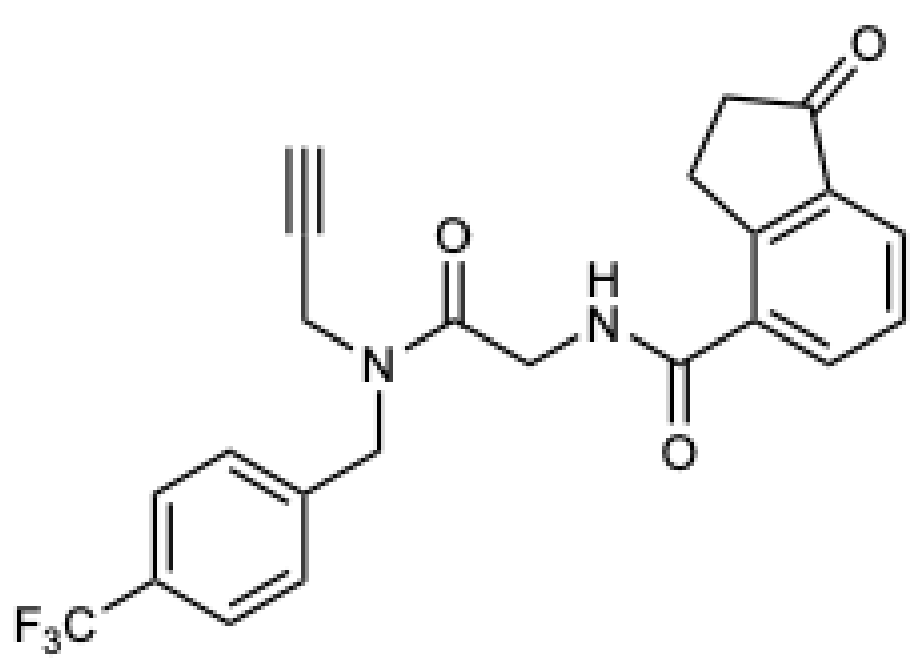

To a solution of **SI-4** (100 mg, 370 µmol, 1 equiv) in DMF was added DIPEA (191 mg, 270 µL, 1.48 mmol, 4.00 equiv), followed by 1-oxo-2,3-dihydro-1H-indene-4-carboxylic acid (72 mg, 407 µmol, 1.10 equiv) and HATU (211 mg, 555 µmol, 1.50 equiv). The reaction was stirred for 14 hours at ambient temperature. The reaction was diluted with ethyl acetate (50 mL) and washed with 1.0 M aqueous HCl (2 × 15 mL), DI water (2 × 15 mL), saturated aqueous $NaHCO_3$ (2 × 15 mL), and brine (2 × 15 mL). The organic layer was dried over Na_2SO_4, filtered, and the filtrate was concentrated under reduced pressure. Purification by flash chromatography (silica gel, gradient 40–60% ethyl acetate in hexanes) afforded **CA-Ind-Alk** as a white foam (137.7 mg, 87% yield) as a complex mixture of rotamers.

TLC (hexanes:ethyl acetate = 2:3): R_f = 0.31 (UV)

^{1}H NMR (600 MHz, $CDCl_3$) δ: 7.96 (m, 1H), 7.89 (m, 1H), 7.65 (d, J = 8.0 Hz, 1H), 7.60 (d, J = 7.9 Hz, 1H), 7.48 (t, J = 7.6 Hz, 1H), 7.42 – 7.34 (m, 2H), 7.24 (br, 1H), 4.80 (s, 2H, major rotamer), 4.76 (s, 2H, minor rotamer), 4.47 (d, J = 4.0 Hz, 2H major rotamer), 4.35 (d, J = 4.1 Hz, 1H), 4.28 (s, 1H), 4.02 (s, 1H), 3.49 – 3.42 (m, 2H), 2.72 (q, J = 5.4 Hz, 2H), 2.38 (t, J = 2.5 Hz, 1H major rotamer), 2.29 (t, J = 2.5 Hz, 1H major rotamer)

^{13}C NMR (101 MHz, $CDCl_3$) δ: 206.53, 168.55, 168.35, 166.81, 154.20, 139.98, 139.02, 138.45, 133.23, 132.53, 132.43, 130.54 (q, J = 32.74 Hz) 128.66, 127.90, 127.22, 126.80, 126.36 (q, J = 3.7 Hz), 125.94 (q, J = 3.7 Hz),124.00 (q, J = 272.21 Hz) 77.55, 76.76, 74.35, 73.36, 49.17, 49.01, 42.04, 41.98, 36.28, 36.19, 35.18, 26.38
^{19}F NMR (376 MHz, $CDCl_3$) δ: -62.50, -62.55
HRMS (ESI$^+$): *m/z* calcd for $C_{23}H_{20}F_3N_2O_3^+$ ($[M+H]^+$): 429.1426; found: 429.1437

Compound SI-6

To a solution of **SI-4** (60 mg, 222 μmol, 1 equiv) in DMF was added DIPEA (57 mg, 77 μL, 444 μmol, 4.00 equiv), followed by 1-oxo-2,3-dihydro-1H-indene-5-carboxylic acid (43 mg, 244 μmol, 1.10 equiv) and HATU (127 mg, 333 μmol, 1.50 equiv). The reaction mixture was stirred at ambient temperature for 14 hours. The reaction mixture was diluted with ethyl acetate (50 mL) and sequentially washed with 1.0 M aqueous HCl (2 × 15 mL), DI water (2 × 15 mL), saturated aqueous $NaHCO_3$ (2 × 15 mL), and brine (2 × 15 mL). The organic layer was dried over Na_2SO_4, filtered, and the filtrate was concentrated under reduced pressure. The crude product was purified by flash column chromatography (silica gel, gradient 40–60% ethyl acetate in hexanes) to afford the product as a white foam (87.6 mg, 92% yield) as a complex mixture of rotamers.

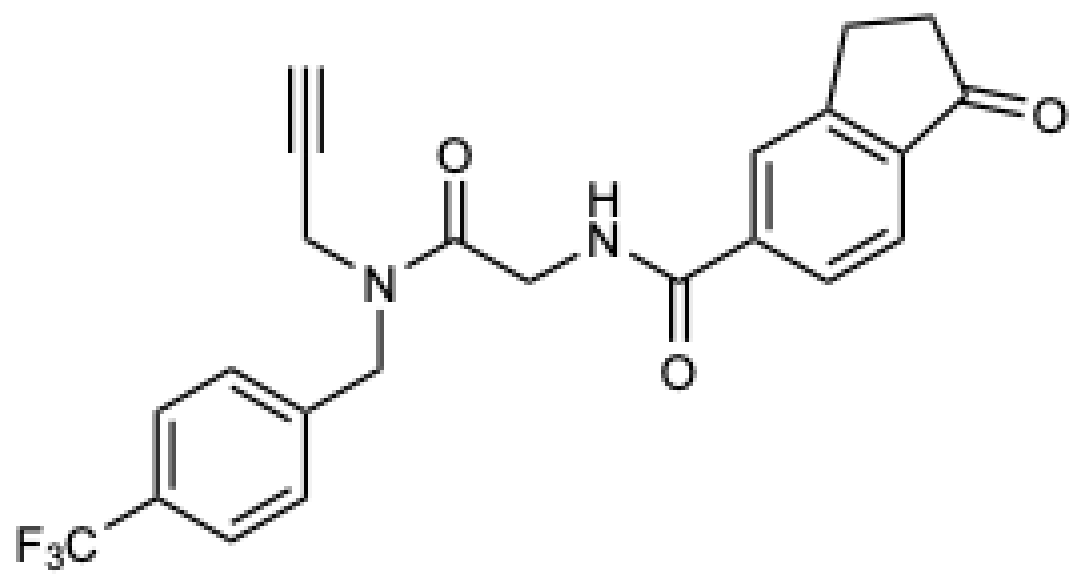

TLC (hexanes:ethyl acetate = 2:3): R_f = 0.29 (UV)

^{1}H NMR (600 MHz, $CDCl_3$) δ: 8.12 (m, 1H), 7.85 (m, 1H), 7.59 (m, 3H), 7.41 (m, 3H), 4.78 (s, 2H, major rotamer), 4.76 (s, 2H, minor rotamer), 4.45 (t, J = 4.3 Hz, 2H, major rotamer), 4.33 (t, J = 4.1 Hz, 2H, minor rotamer), 4.26 (t, J = 2.6 Hz, 2H, minor rotamer), 4.02 (t, J = 3.1 Hz, 1H, major rotamer), 3.22 – 3.13 (m, 2H), 2.77 – 2.67(m, 2H), 2.40 – 2.33 (m, 1H, minor rotamer), 2.31 – 2.24 (m, 1H, major rotamer)

^{13}C NMR (150 MHz, $CDCl_3$) δ: 206.42, 168.61, 168.43, 166.66, 166.37, 158.54, 155.38, 140.11, 139.55, 139.40, 139.16, 137.38, 133.81, 133.31, 130.28, 128.67, 127.22, 126.32 (q, *J* = 3.7 Hz), 125.89 (q, *J* = 3.2 Hz), 124.08, 123.94 (q, J = 279 Hz), 122.16, 77.67, 77.58, 76.79, 74.33, 74.24, 73.31, 73.25, 49.13, 48.97, 42.13, 42.06, 36.58, 36.19, 35.08, 26.04, 25.91

^{19}F NMR (376 MHz, $CDCl_3$) δ: -62.48(major rotamer), -62.54 (minor rotamer)
HRMS (ESI$^+$): *m/z* calcd for $C_{23}H_{20}F_3N_2O_3^+$ ([M+H]$^+$): 429.1426; found: 429.1438

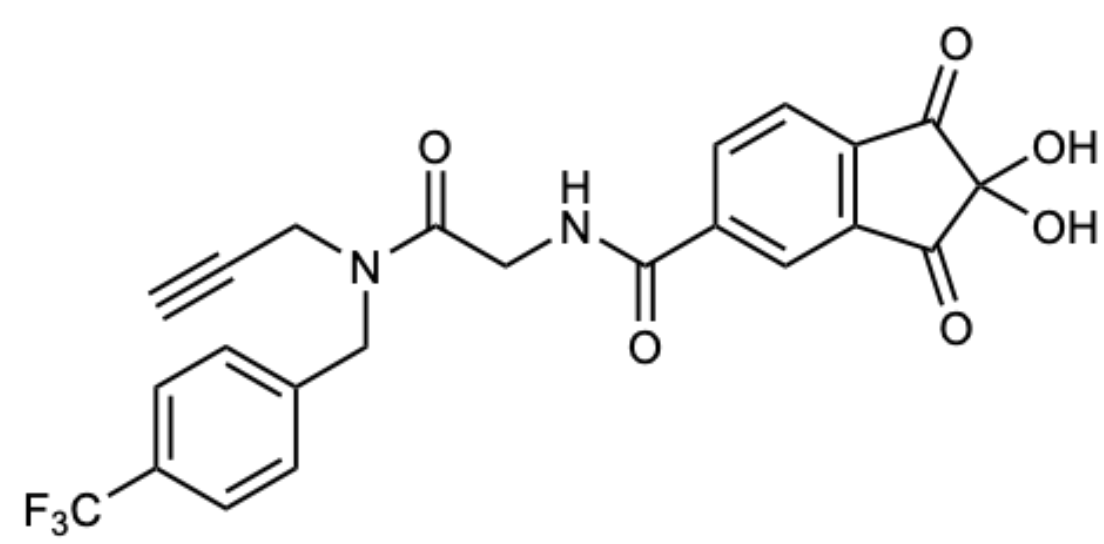

isoCA-Alk

Selenium dioxide (79 mg, 716 μmol, 2.1 equiv) was added to a solution of **SI-6** (146 mg, 341 μmol, 1 equiv) in 1,4-dioxane (1.5 mL) and DI water (150 μL). The reaction mixture was heated to reflux in a 96 ºC oil bath for 4 hours. After cooling to ambient temperature, the mixture was diluted with ethyl acetate (15 mL) and washed successively with DI water (2 × 10 mL), 1 M aqueous HCl (2 × 10 mL). The organic layer was washed with brine (1 × 10 mL), dried over Na_2SO_4, filtered, and the filtrate was concentrated under reduced pressure. The crude mixture was purified by reverse-phase HPLC (25-50% MeCN with 0.1% TFA in milliQ water with 0.1% TFA), and lyophilization of the collected fractions yielded the product as a white powder (18 mg, 11% yield) as a complex mixture of rotamers.

TLC (hexanes:acetone = 1:2): R_f = 0.39 (UV)

^{1}H NMR (600 MHz, 10:1 $CD_3CN + D_2O$) δ 8.38 (1H, m), 8.30 (1H, m), 8.07 (1H, m), 7.65 (2H, dd, J = 13.8, 7.9 Hz), 7.48 (2H, dd, *J* = 24.9, 7.9 Hz), 4.79 (s, 2H, minor rotamer), 4.71 (s, 2H, major rotamer), 4.40 (s, 2H, major rotamer), 4.23 (s, 2H, minor rotamer), 4.16 (2H, s), 2.69 (1H, s, major rotamer), 2.55 (1H, s, minor rotamer)

^{13}C NMR (151 MHz, 10:1 $CD_3CN + D_2O$) δ: 169.78, 169.64, 169.19, 168.83, 166.68, 153.87, 142.62, 141.88, 136.91, 136.70, 133.61, 131.39, 130.95, 130.53, 129.58, 129.21, 128.41, 126.43, 126.13, 124.30, 123.56, 79.43, 78.86, 74.53, 73.36, 50.20, 49.94, 42.32, 42.16, 37.46, 35.89

^{19}F NMR (376 MHz, CD_3CN + 10% D_2O) δ: -63.68(major rotamer), -63.71 (minor rotamer), -76.55 (reference TFA)

HRMS (ESI$^+$): *m/z* calcd for $C_{23}H_{18}F_3N_2O_6^+$ ($[M+H]^+$): 475.1117; found: 475.1113.

CA-Alk

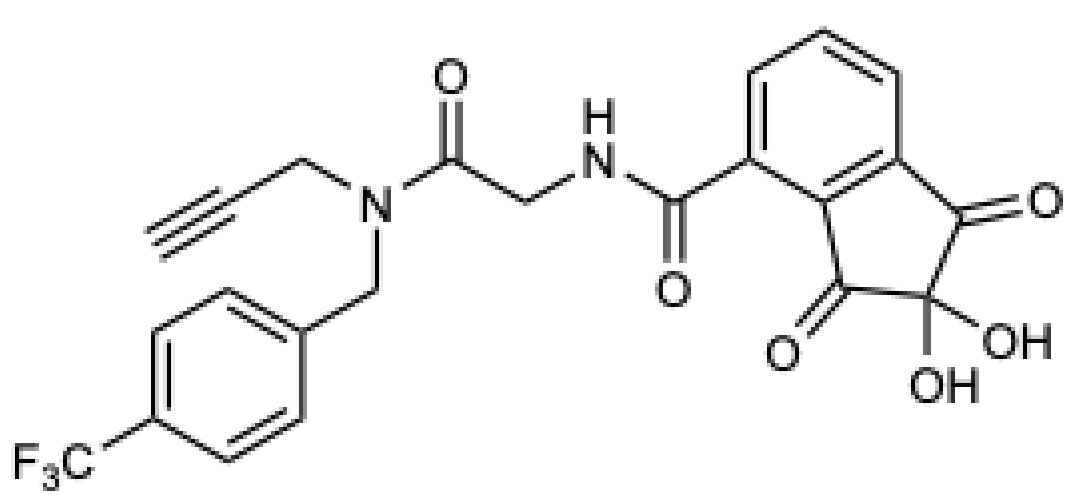

Selenium dioxide (140 mg, 1.3 mmol, 2.2 equiv) was added to a solution of **CA-Ind-Alk** (245 mg, 590 μmol, 1 equiv) in 1,4-dioxane (2.6 mL) and DI water (260 μL). The reaction mixture was heated to reflux in an oil bath at 96 ºC for 4 hours. After cooling to ambient temperature, the mixture was diluted with ethyl acetate (15 mL) and washed successively with DI water (2 × 10 mL), 1 M aqueous HCl (2 × 10 mL). The organic layer was washed with brine (1 × 10 mL), dried over Na_2SO_4, filtered, and the filtrate was concentrated under reduced pressure. The crude mixture was purified by reverse-phase HPLC (25-50% MeCN with 0.1% TFA in milliQ water with 0.1% TFA), and lyophilization of the collected fractions yielded the product as a white powder (26 mg, 10 % yield) as a complex mixture of rotamers.

TLC (hexanes:acetone = 1:2): R_f = 0.42 (UV)

^{1}H NMR (600 MHz, 10:1 $CD_3CN + D_2O$) δ: 8.35-7.9(3H, m), 7.65 (2H, m), 7.49 (2H, m), 4.79 (2H, s, minor rotamer), 7.72 (2H, s, major rotamer), 4.47 (2H, s, major rotamer), 4.31 (2H, s, minor rotamer), 4.17 (2H, m, both rotamers), 2.70 (1H, m, major rotamer), 2.56 (1H, m, minor rotamer)

^{13}C NMR (151 MHz, 10:1 $CD_3CN + D_2O_3$) δ: 197.56, 196.36, 170.13, 166.60, 166.46, 142.43, 141.74, 140.89, 138.70, 138.65, 138.40, 136.25, 136.16, 134.63, 134.49, 132.32, 131.76, 129.40, 128.69, 128.63 127.41,127.16 (q, J = 272 Hz) 126.66, 126.35, 88.13, 79.37, 78.82, 75.11, 74.08, 73.94, 50.73, 50.30, 43.08, 42.91, 37.84, 36.42.

^{19}F NMR (376 MHz, CD_3CN + 10% D_2O) δ: -63.71(major rotamer), -63.74 (minor rotamer), -76.55 (reference TFA)

HRMS (ESI$^+$): *m/z* calcd for $C_{23}H_{18}F_3N_2O_6^+$ ($[M+H]^+$): 475.1117; found: 475.1122

Compound SI-7

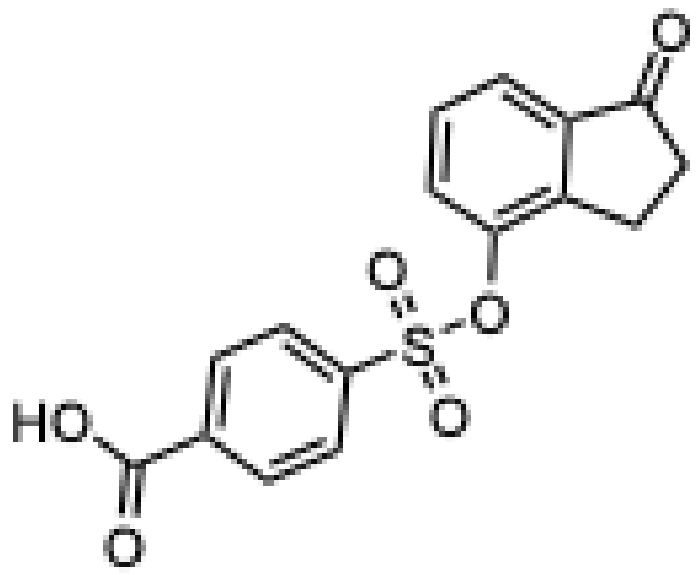

4-(Chlorosulfonyl)benzoic acid (882 mg, 4.00 mmol, 1.00 equiv) and 4-hydroxy-2,3-dihydro-1H-inden-1-one (622 mg, 4.20 mmol, 1.05 equiv) were added to a flame-dried 25 mL round-bottom flask equipped with a magnetic stir bar. The vessel was sealed with a rubber septum and placed under an argon atmosphere via balloon. Anhydrous DCM (20.0 mL) was added, followed by triethylamine (1.01 g, 1.39 mL, 10.0 mmol, 2.50 equiv). The reaction mixture was stirred at ambient temperature for 4 hours, then quenched by the addition of 1 M aqueous HCl (15 mL). The mixture was transferred to a separatory funnel and the organic layer was separated. The aqueous layer was re-extracted with DCM (3 × 15 mL). The combined organic layers were washed with saturated aqueous NaCl (1 × 15 mL), dried over Na_2SO_4, filtered through a cotton plug, and the filtrate was concentrated by rotary evaporation to afford a tan solid. The crude material was purified by flash chromatography (silica gel, gradient 0–10% MeOH in DCM + 0.1% AcOH) to afford **SI-7** as a white solid (1.29 g, 3.88 mmol, 97% yield).

TLC

^{1}H NMR (600 MHz, $CDCl_3$) δ: 8.30 (2H, d, *J* = 8.1 Hz), 8.03 (2H, d, *J* = 8.1 Hz), 7.71 (1H, d, *J* = 7.6 Hz), 7.35 (1H, t, *J* = 7.7 Hz), 7.17 (1H, d, *J* = 7.9 Hz), 3.00 (1H, t, *J* = 5.6 Hz), 2.66 (1H, t, *J* = 6.0 Hz)

^{13}C NMR (151 MHz, $CDCl_3$) δ: 205.47, 167.87, 147.79, 147.16, 140.21, 140.09, 131.21, 129.25, 128.77, 127.93, 123.14, 36.08, 22.94

HRMS (ESI$^+$): *m/z* calcd for $C_{16}H_{12}O_6S^+$ ([M+H]$^+$): 333.0433 found: 333.0432

Compound SI-8

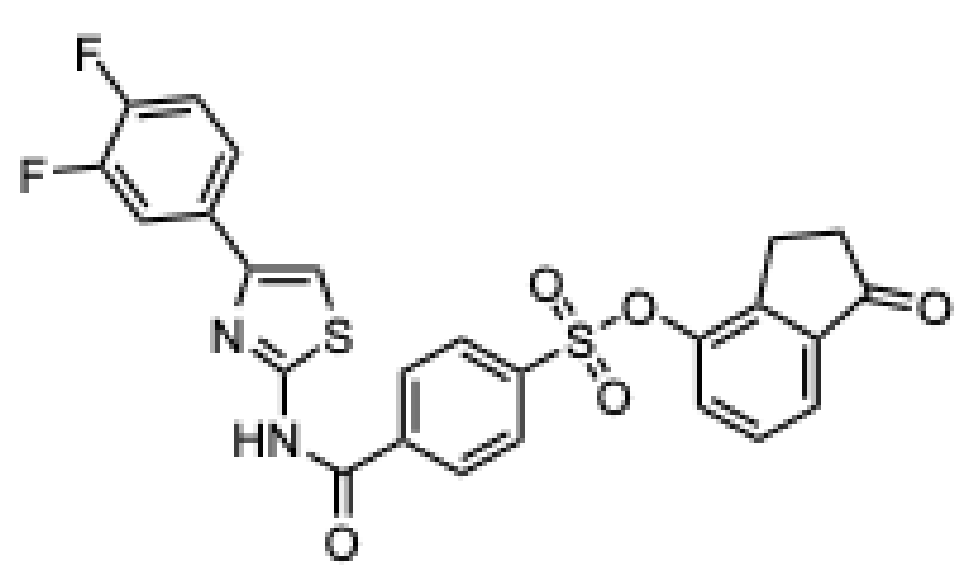

To a solution of **SI-7** (400 mg, 963 μmol, 1.00 equiv) in anhydrous DMF (4.81 mL) was added triethylamine (97.4 mg, 134 μL, 963 μmol, 1.00 equiv), followed by HATU (549 mg, 1.44 mmol, 1.50 equiv) and 4-(3,4-difluorophenyl)thiazol-2-amine (225 mg, 1.06 mmol, 1.10 equiv). The reaction mixture was stirred at ambient temperature for 14 hours. The reaction was diluted with ethyl acetate (50 mL) and sequentially washed with 1.0 M aqueous HCl (2 × 15 mL), DI water (2 × 15 mL), saturated aqueous $NaHCO_3$ (2 × 15 mL), and brine (2 × 15 mL). The organic layer was dried over Na_2SO_4, filtered, and the filtrate was concentrated under reduced pressure. The crude product was purified by flash column chromatography (silica gel, gradient 20–40% ethyl acetate in hexanes) to afford **SI-8** as a white solid (473 mg, 898 μmol, 93.3% yield).

TLC (hexanes:ethyl acetate = 1:1): R_f = 0.46 (UV, DNP stain)

^{1}H NMR (600 MHz, $(CD_3)_2SO$) δ: 8.34 (2H, d, *J* = 8.5 Hz), 8.11 (2H, d, *J* = 8.6 Hz), 7.97 (1H, ddd, *J* = 12.1, 7.8, 2.2 Hz), 7.86 (1H, s), 7.81 (1H, ddd, *J* = 7.7, 4.3, 2.3 Hz), 7.65 (1H, dd, *J* = 7.6, 0.9 Hz), 7.52 (3H, m), 7.37 (1H, dd, *J* = 8.0, 0.9 Hz), 2.82 (3H, t, *J* = 5.7 Hz), 2.60 (2H, t, *J* = 5.7 Hz)

^{13}C NMR (151 MHz, $(CD_3)_2SO$) δ 205.40, 164.42, 158.83, 150.15 (dd, J_{CF} = 243.6, 13.6 Hz), 149.53 (dd, J_{CF} = 246.6, 12.3 Hz), 147.75, 147.04, 140.04, 138.37, 137.98, 132.38, 130.31, 130.06, 128.89, 128.49, 123.02, 122.90, 118.45 (d, J_{CF} = 17.6 Hz), 115.06 (d, J_{CF} = 18.1 Hz), 110.54, 36.09, 22.83.

^{19}F NMR (376 MHz, $(CD_3)_2SO$) δ -138.10.63, -139.52

HRMS (ESI$^+$): *m/z* calcd for $C_{25}H_{15}F_2N_2O_5S_2^-$ ($[M-H]^-$): 525.0390; found: 525.0395

Compound Lig-Nin

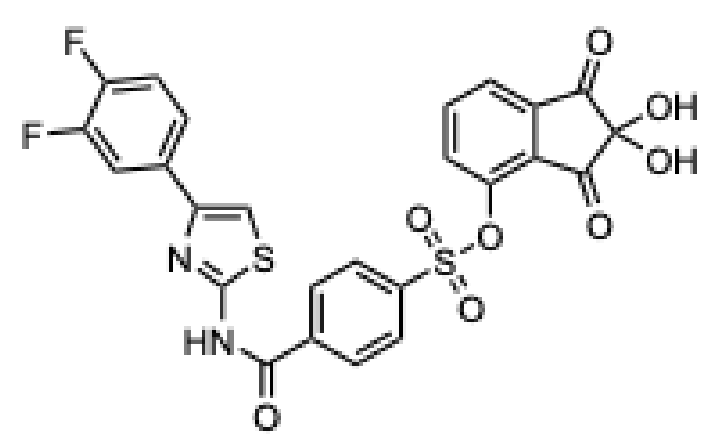

Selenium dioxide (52.7 mg, 475 µmol, 2.50 equiv) was added to a solution of **SI-8** (100 mg, 190 µmol, 1 equiv) in 1,4-dioxane (345 µL) and DI water (34.5 µL). The reaction mixture was heated to reflux in a 96 °C oil bath for 4 hours. After cooling to ambient temperature, the mixture was diluted with ethyl acetate (15 mL) and washed successively with DI water (2 × 10 mL) and 1 M aqueous HCl (2 × 10 mL). The organic layer was washed with brine (1 × 10 mL), dried over Na_2SO_4, filtered, and the filtrate was concentrated under reduced pressure. The crude residue was purified by flash chromatography (silica gel, gradient 30–70% acetone in hexanes) to afford a pink solid. Final purification was performed by reverse-phase HPLC (5–50% MeCN with 0.1% TFA in milliQ water with 0.1% TFA), and lyophilization of the collected fractions yielded **Lig-Nin** as a fluffy pink powder (25 mg, 44 µmol, 23% yield).

TLC (hexanes:acetone = 1:1): R_f = 0.64 (UV)

^{1}H NMR (600 MHz, 10:1 CD_3CN + D_2O) δ: 8.14 (2H, d, *J* = 8.6 Hz), 8.00 (2H, d, *J* = 8.6 Hz), 7.98 (1H, t, *J* = 7.9 Hz), 7.96 (1H, dd, *J* = 7.7, 1.2 Hz), 7.83 (1H, m), 7.71 (1H, m), 7.67 (1H, dd, *J* = 7.8, 1.2 Hz), 7.44 (1H, s), 7.31 (1H, dd, *J* = 10.7, 8.3 Hz)

^{13}C NMR (151 MHz, 10:1 CD_3CN + D_2O_3) δ: 195.42, 193.19, 164.81, 158.96, 150.81 (dd, J_{CF} = 246.3, 13.2 Hz), 150.38 (dd, J_{CF} = 246.5, 12.7 Hz), 148.22, 145.72, 141.04, 139.43, 138.80, 137.96, 132.27, 131.86, 131.36, 129.62, 129.55, 124.02, 123.02, 118.59, 115.46(d, J_{CF} = 18.7 Hz), 109.93, 87.39.

^{19}F NMR (376 MHz, CD_3CN + 10% D_2O) δ: -76.55 (reference TFA), -140.63, -141.83

HRMS (ESI$^+$): *m/z* calcd for $C_{25}H_{15}F_2N_2O_8S_2^+$ ($[M+H]^+$): 572.0238; found: 572.0253

Spectral Data

SI-1 1H

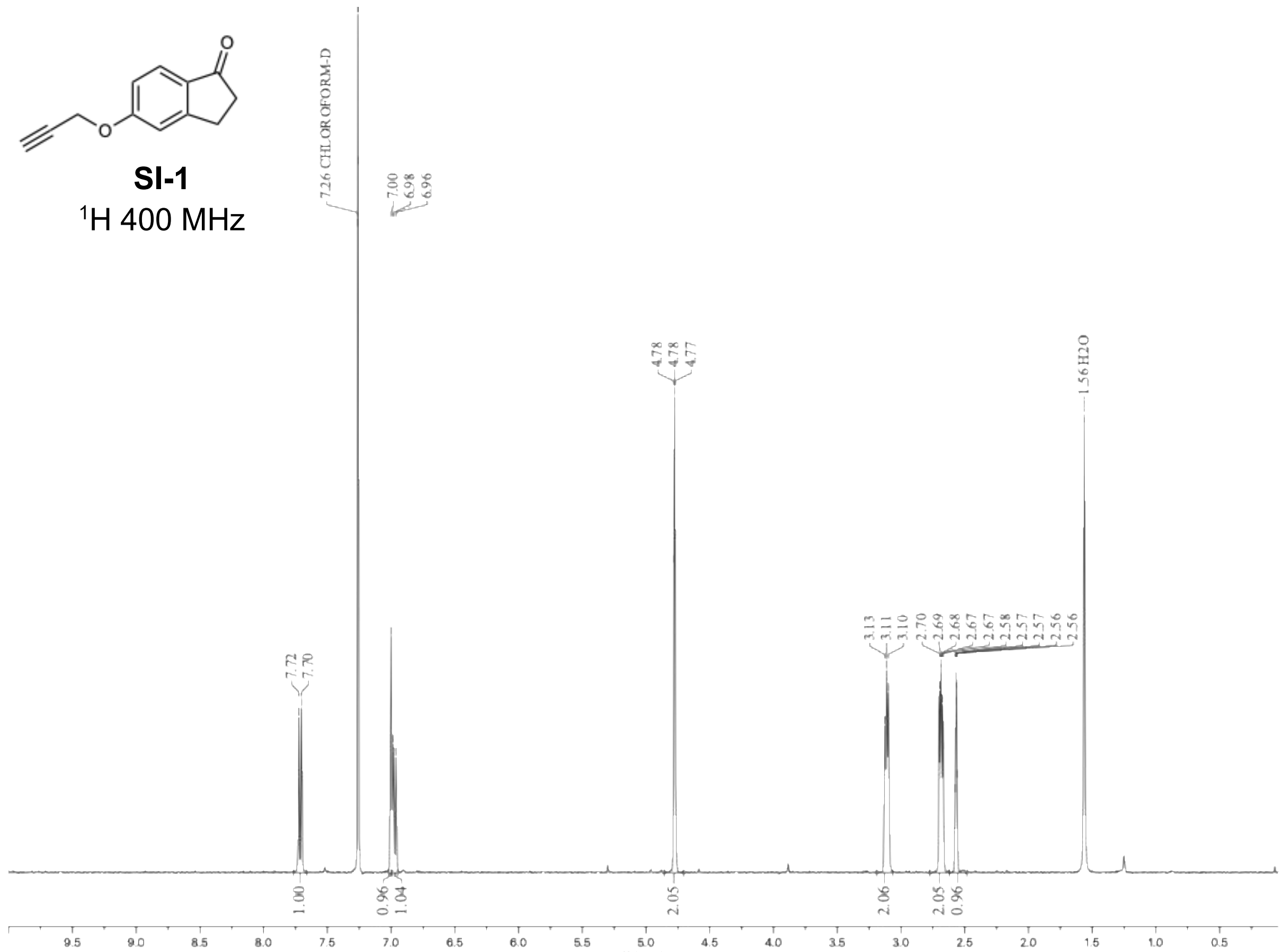

SI-1 13C

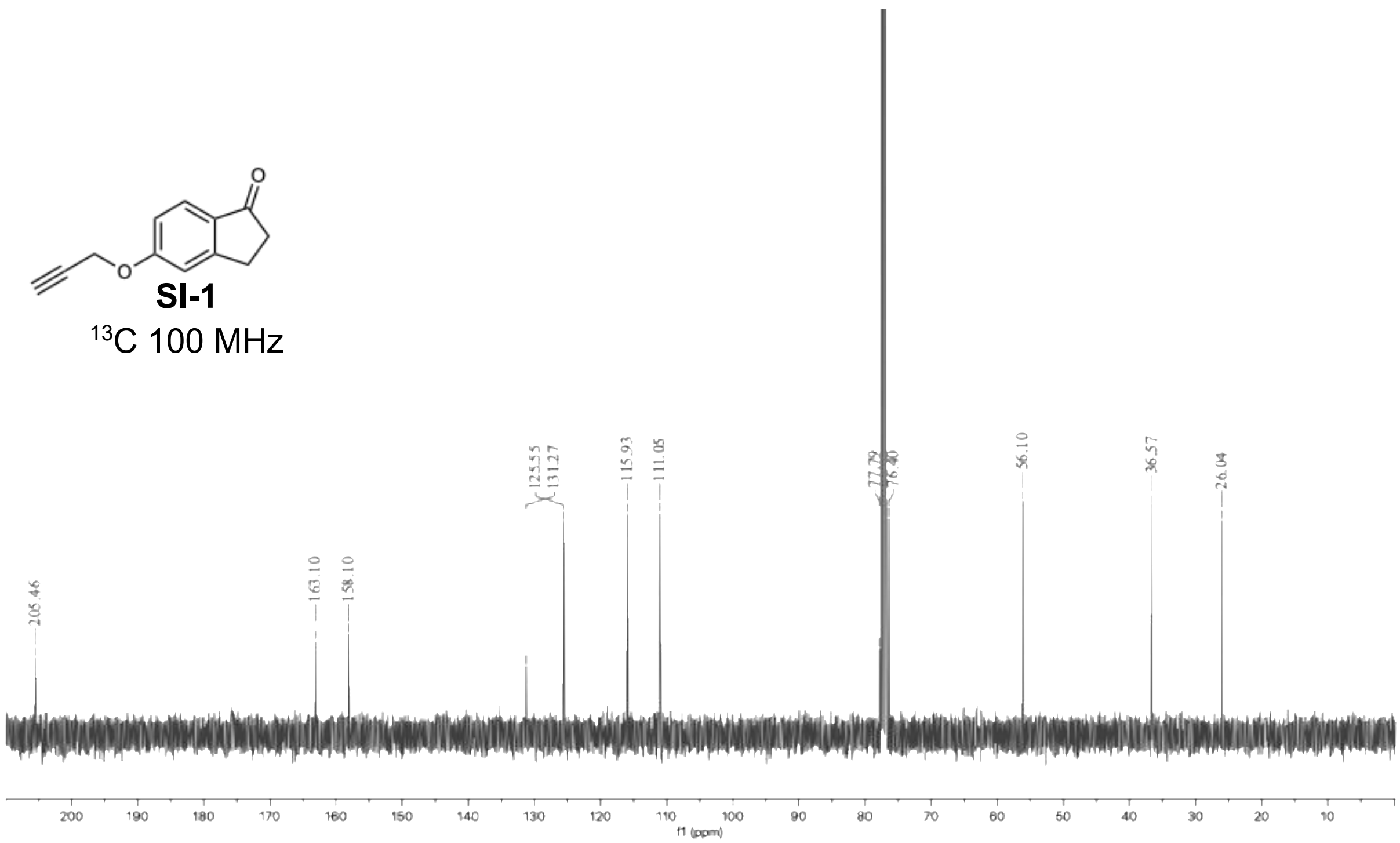

SI-1
^{13}C 100 MHz
205.46
163.10
158.10
125.55
131.27
115.93
111.05
77.79
76.40
56.10
36.57
26.04
f1 (ppm)

Nin-Alk 1H

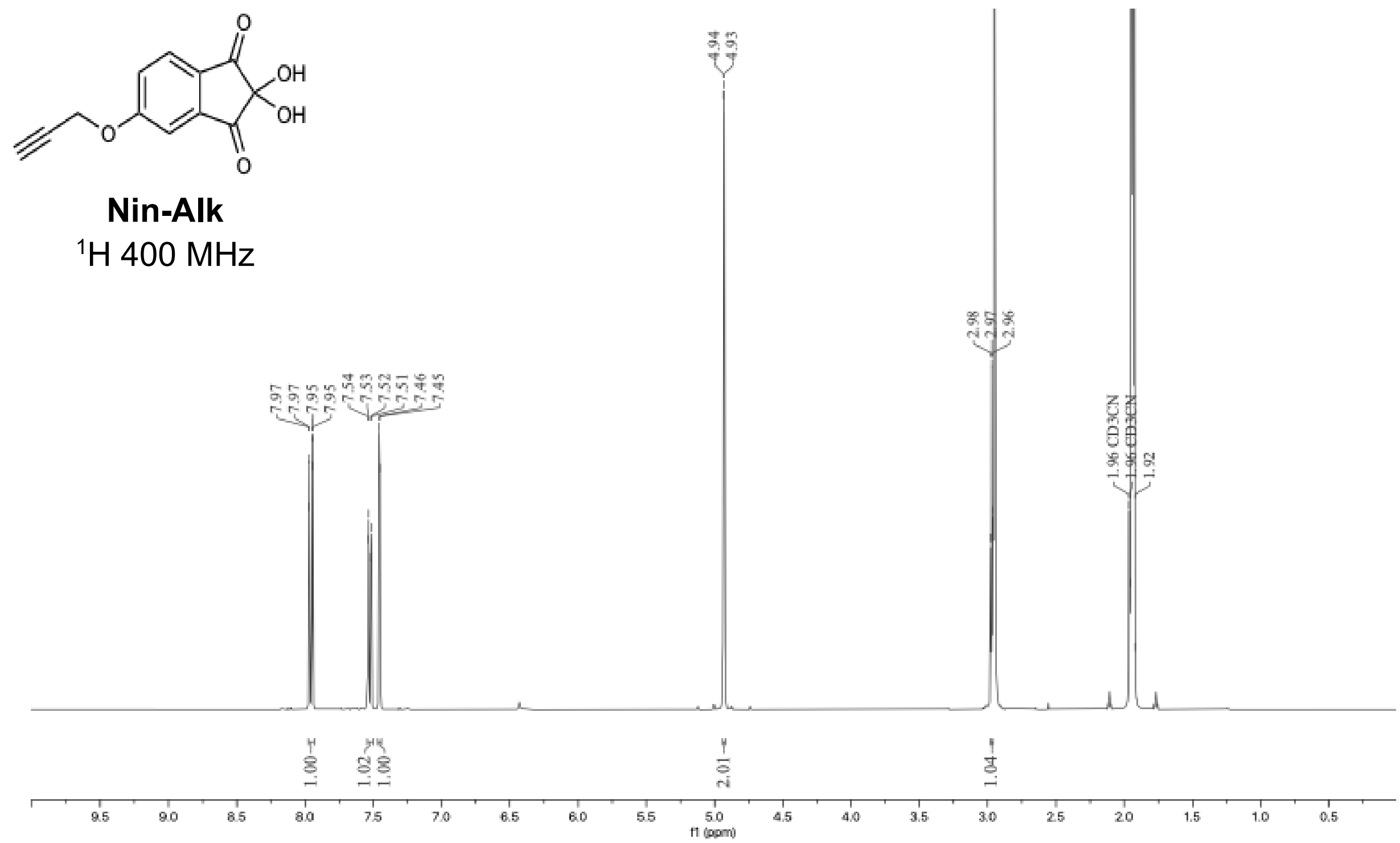

Nin-Alk 13C

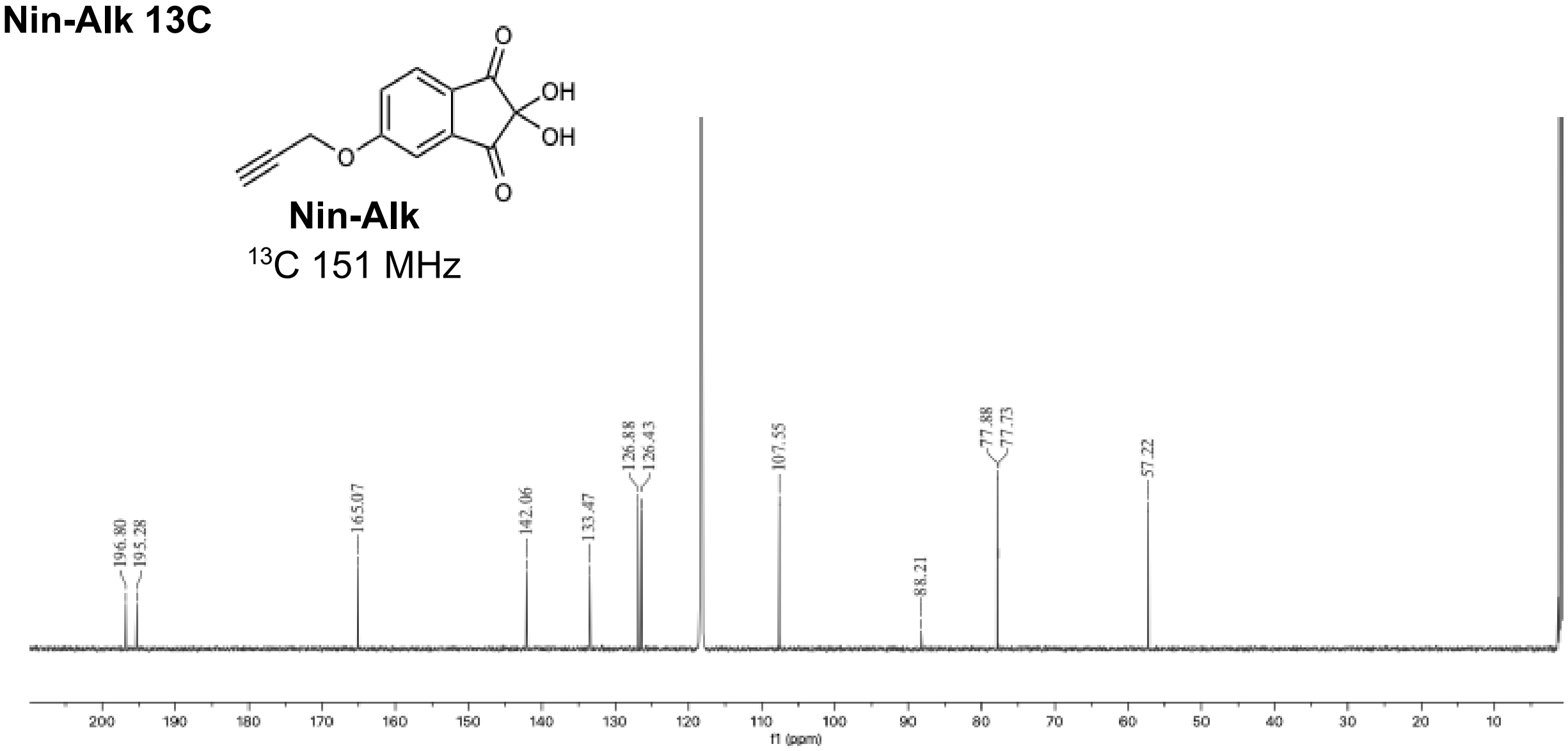

SI-2 1H

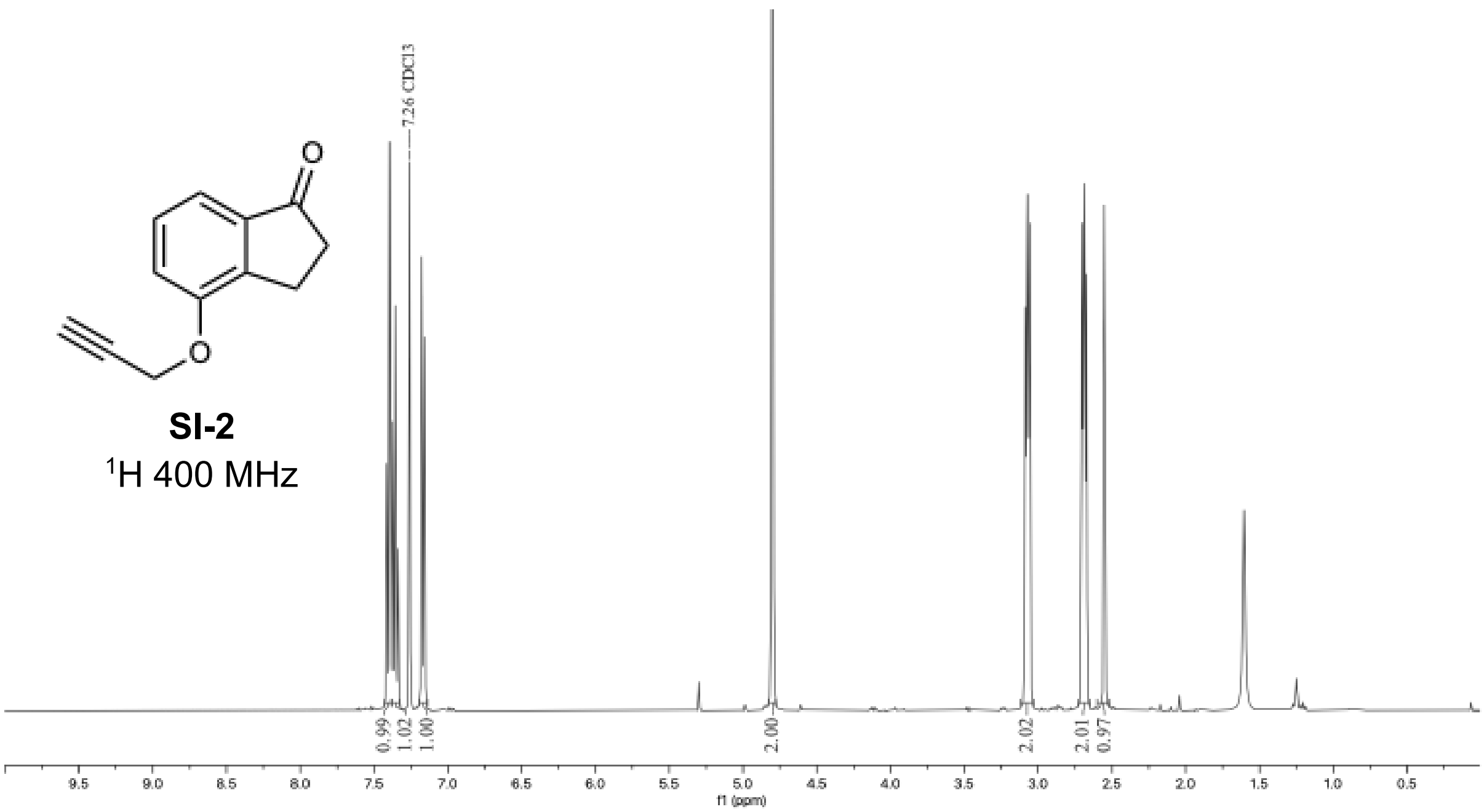

SI-2 13C

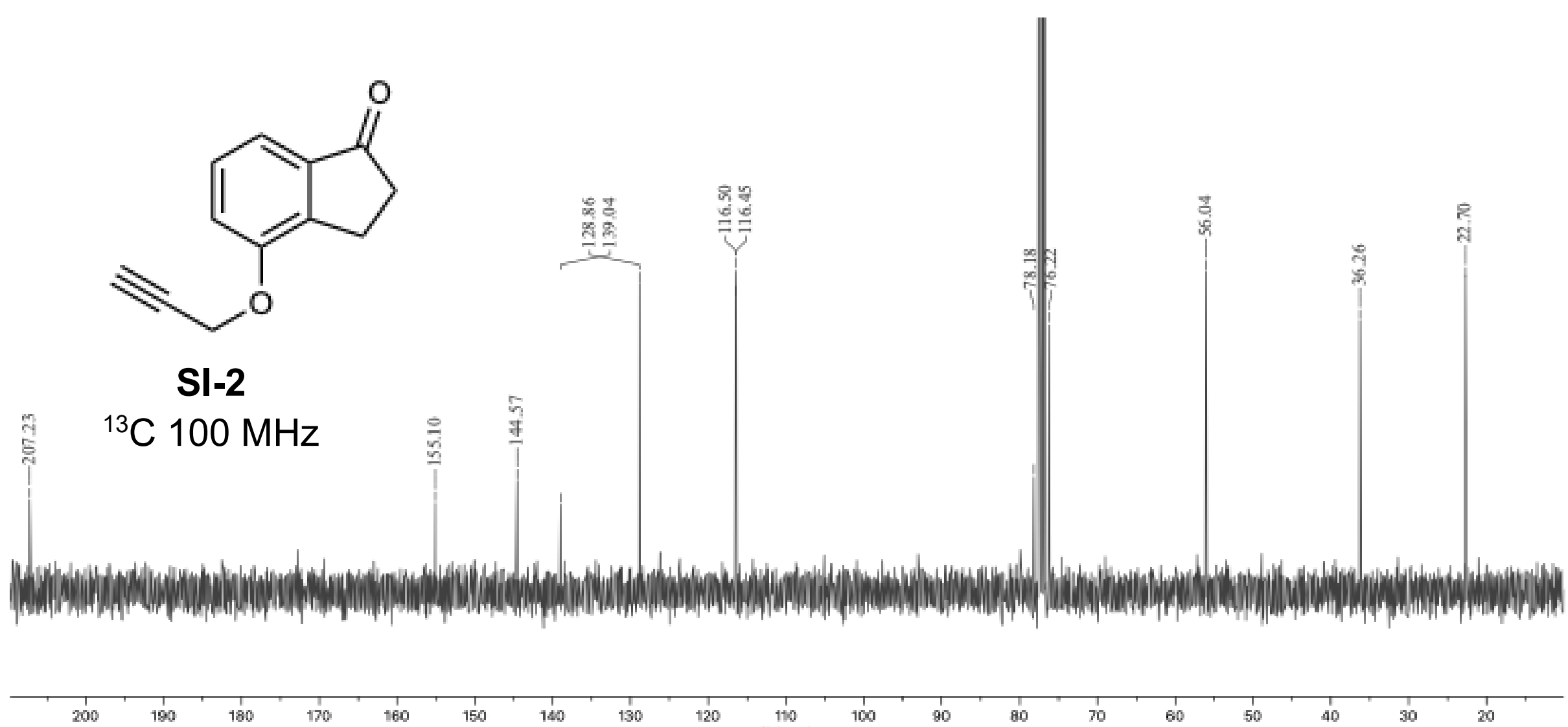

4-Nin-Alk 1H

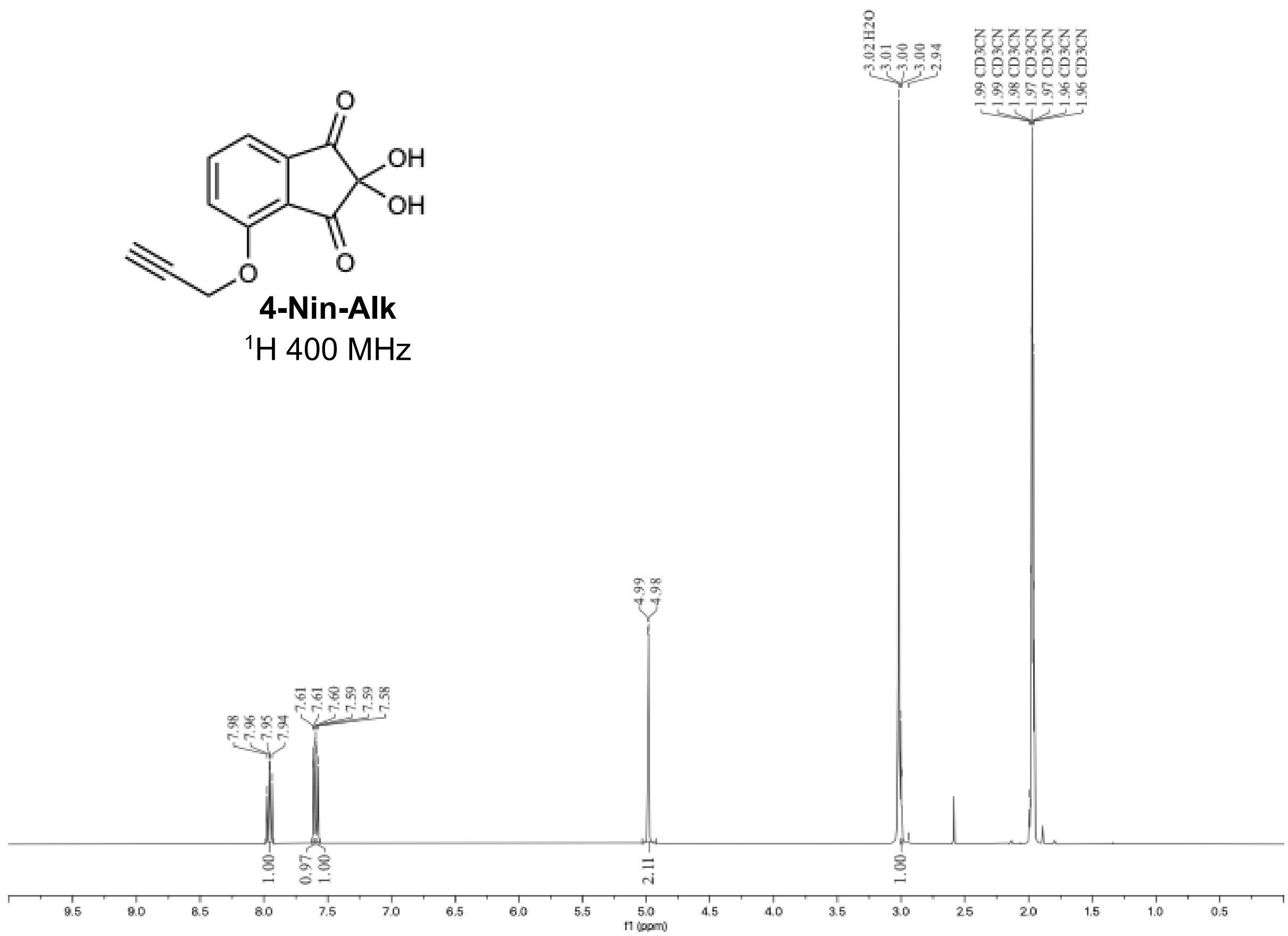

4-Nin-Alk
^{1}H 400 MHz
3.02 H2O
3.01
3.00
3.00
2.94
1.99 CD3CN
1.99 CD3CN
1.98 CD3CN
1.97 CD3CN
1.97 CD3CN
1.96 CD3CN
1.96 CD3CN
4.99
4.98
7.98
7.96
7.95
7.94
7.61
7.61
7.60
7.59
7.59
7.58
1.00
0.97
1.00
2.11
1.00
f1 (ppm)

4-Nin-Alk 13C

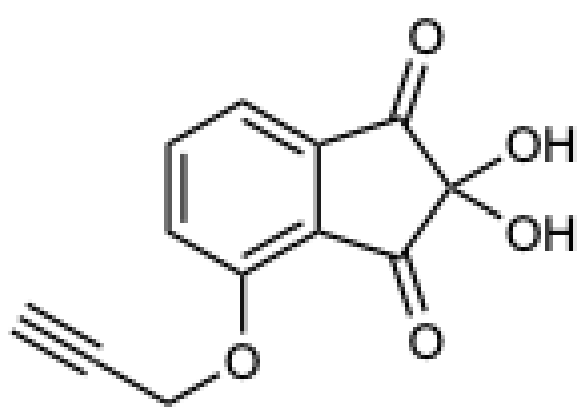

4-Nin-Alk

^{13}C 151 MHz

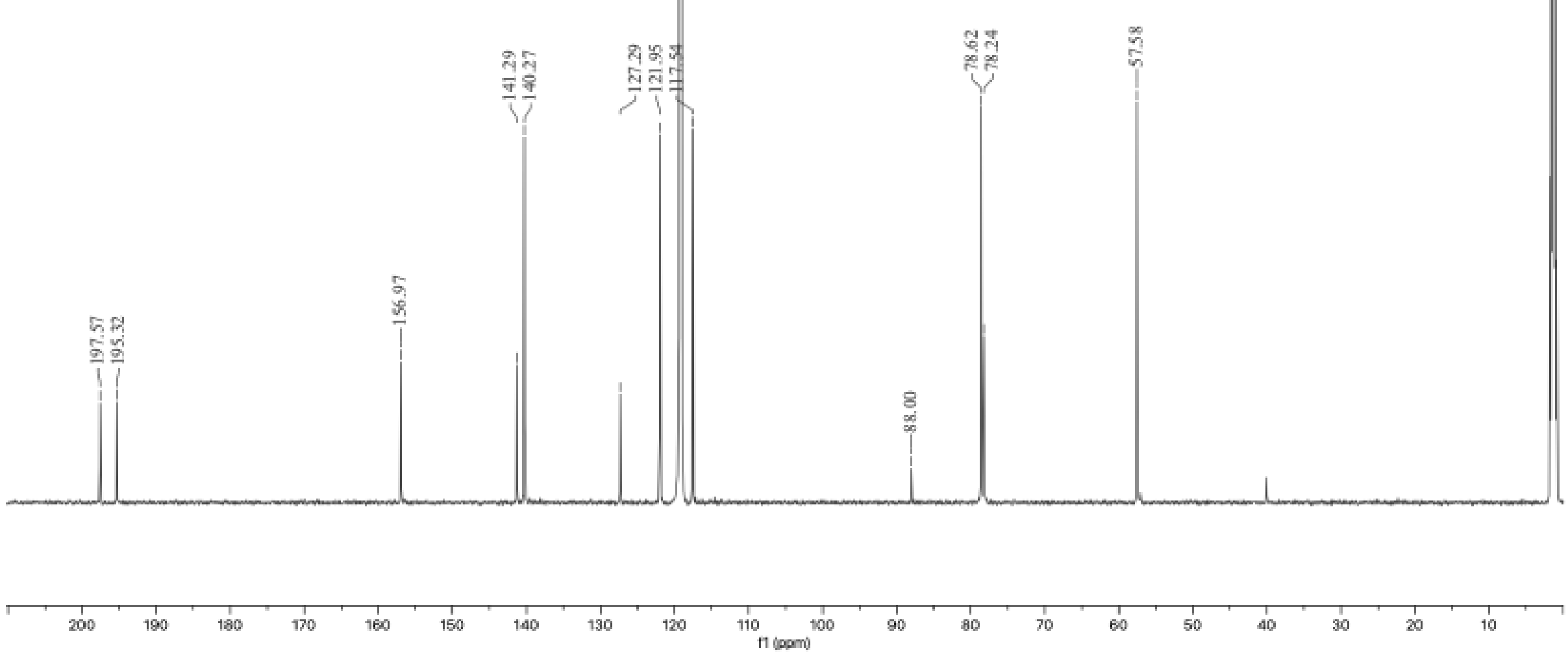

SI-3 1H

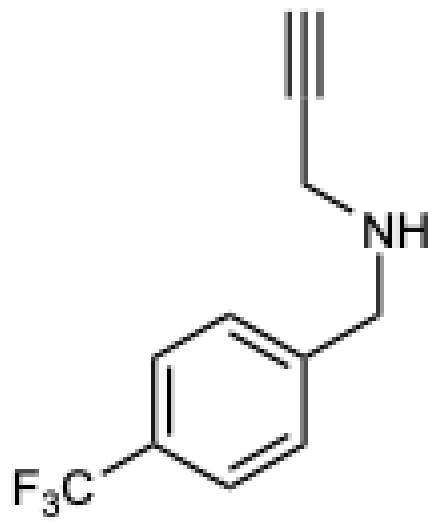

SI-3
^{1}H 400 MHz

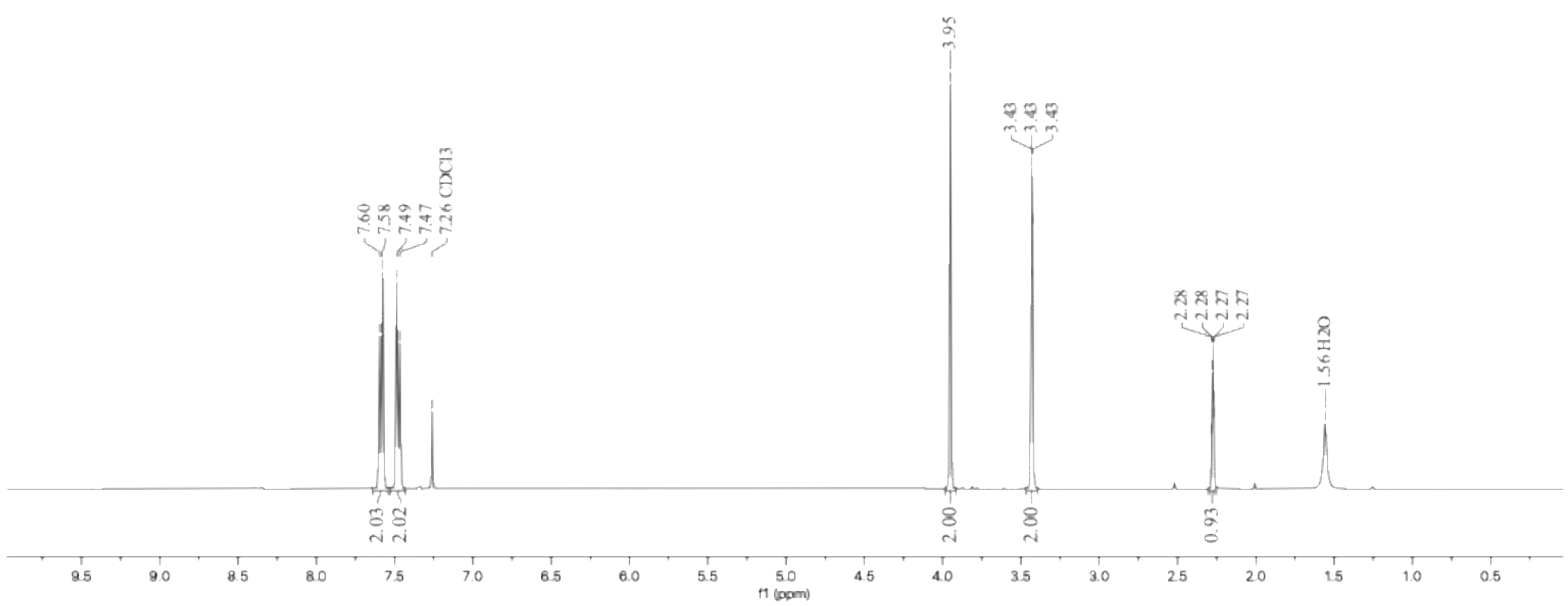

SI-3 13C

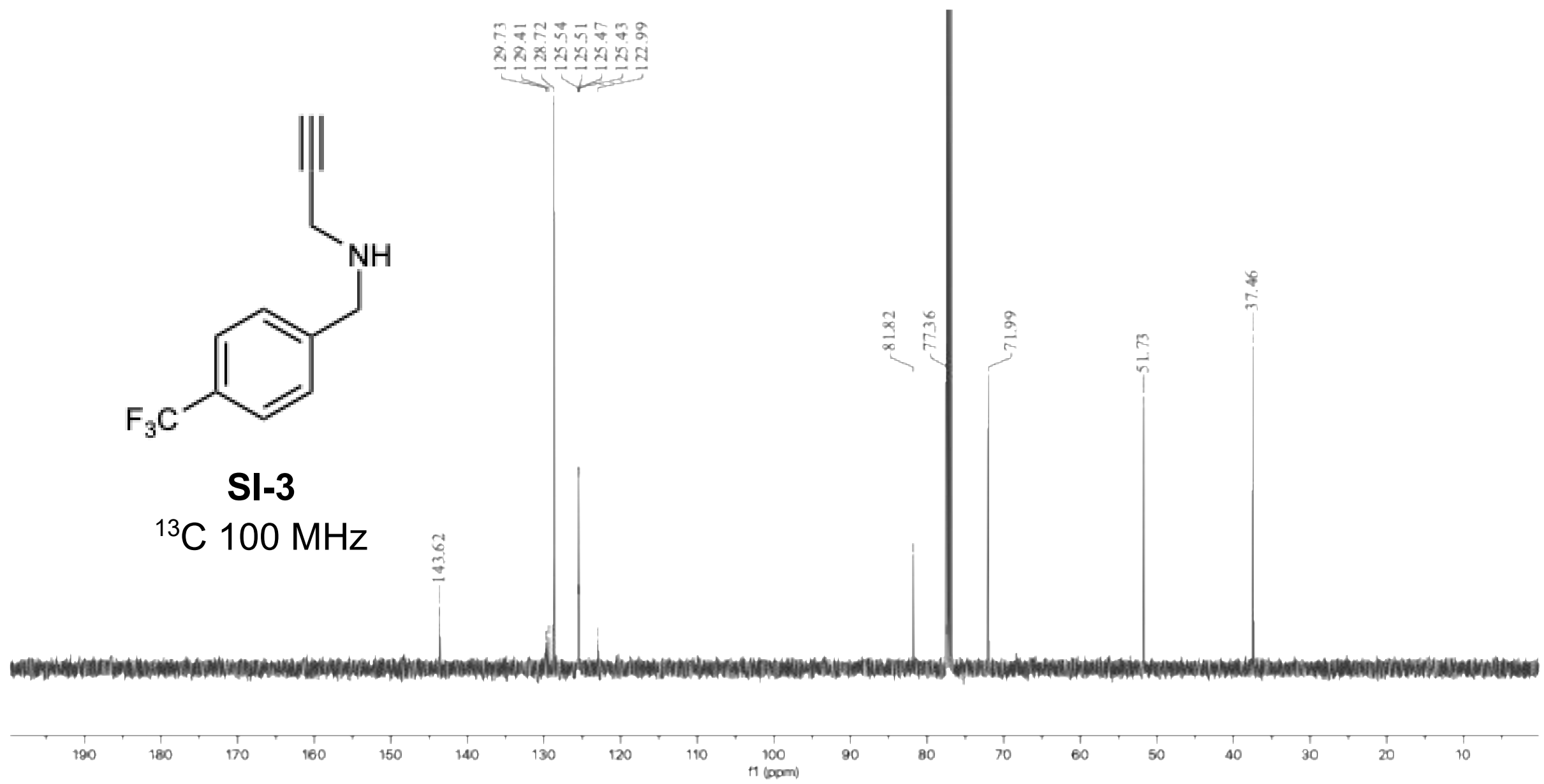

SI-3 19F

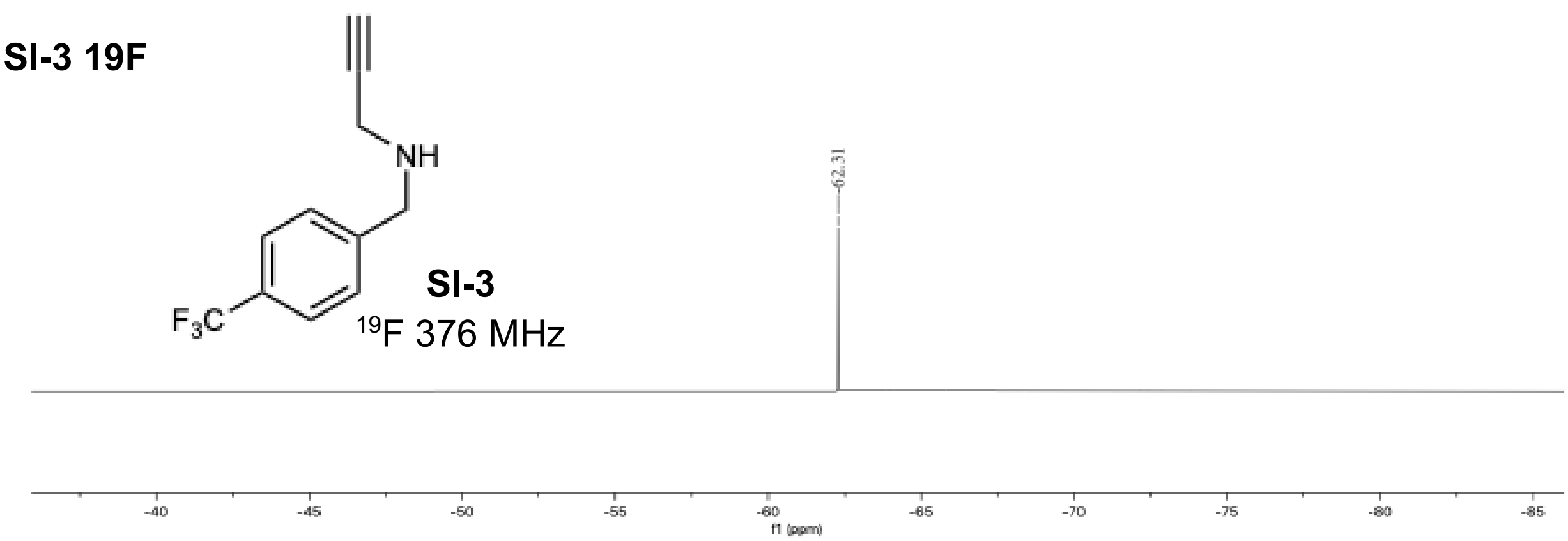

SI-4 1H

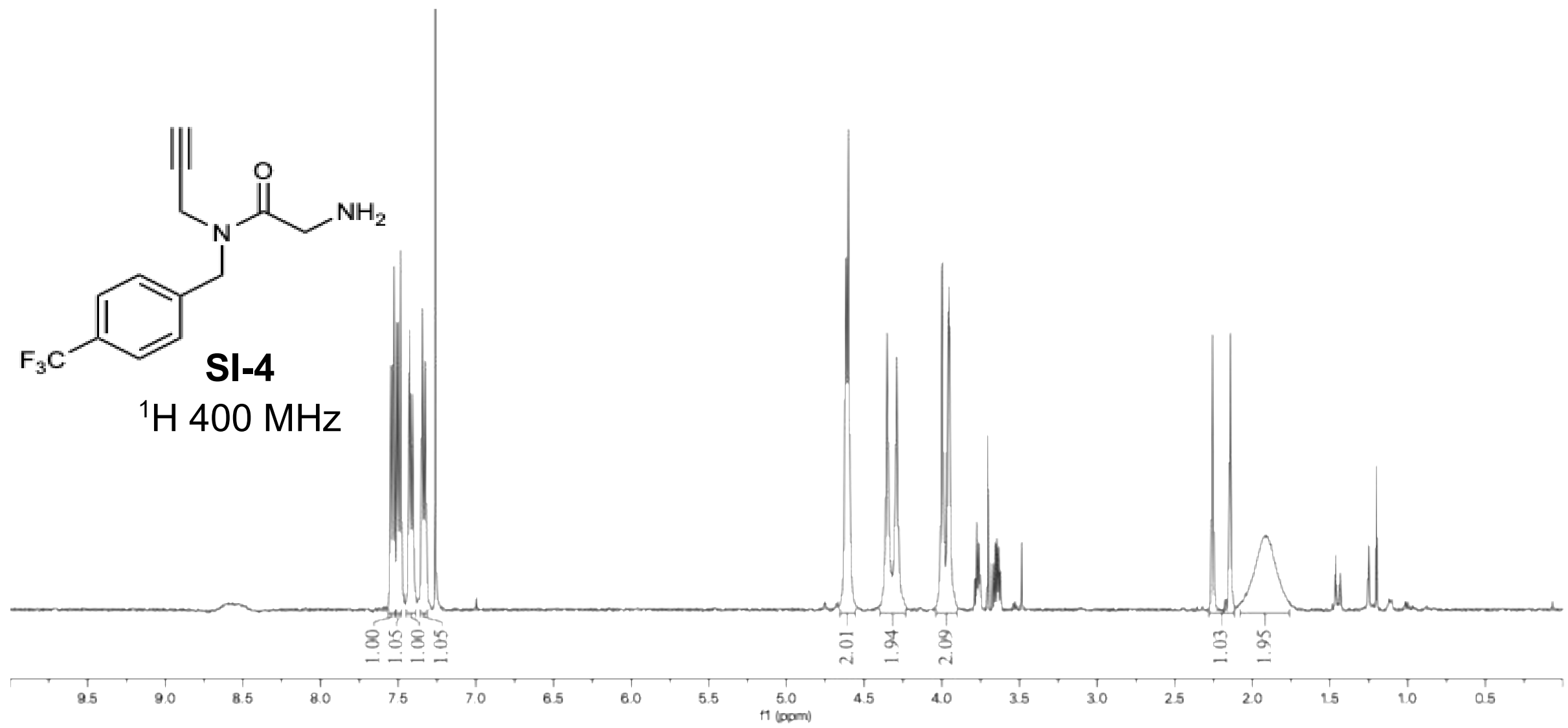

SI-4 13C

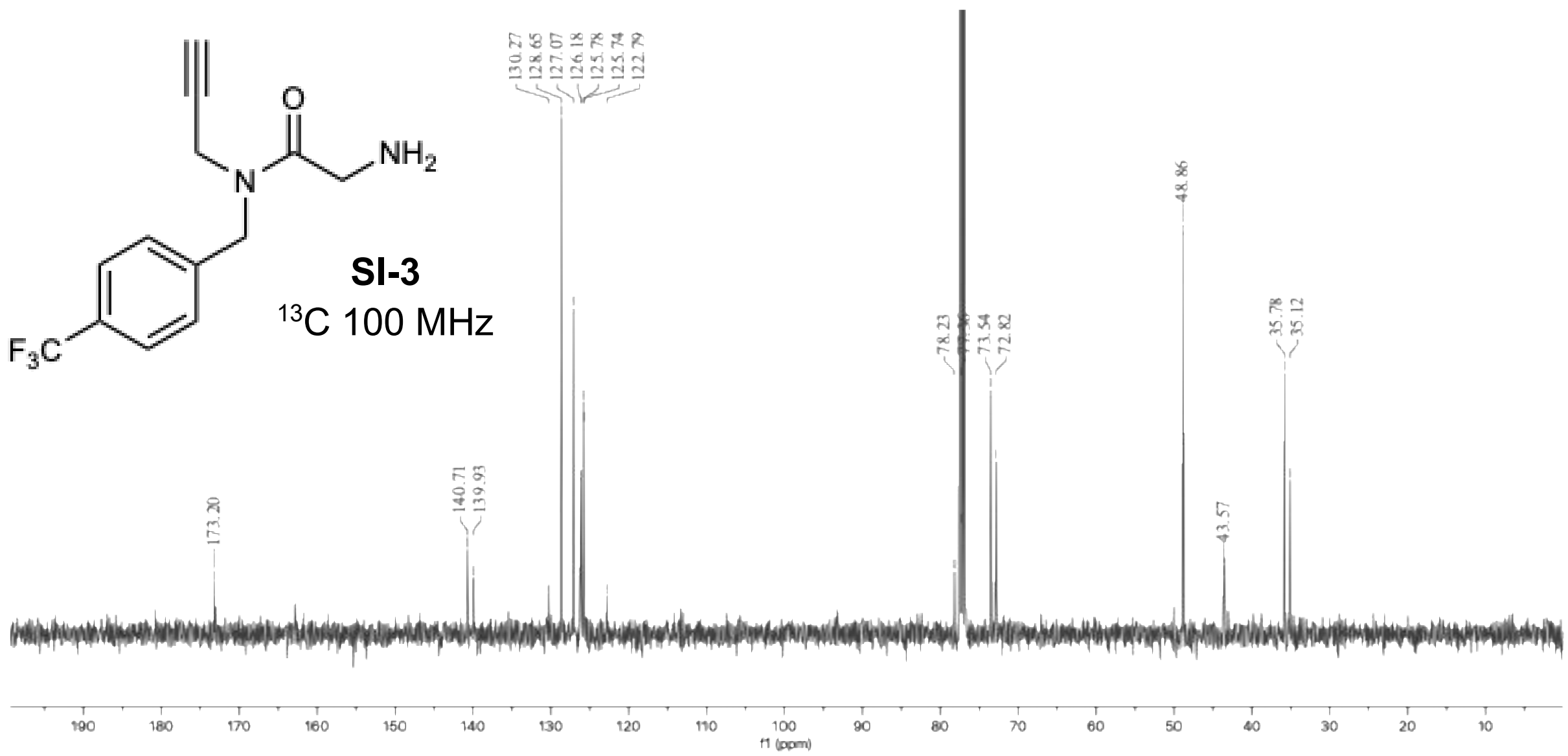
NH2
F3C
SI-3
13C 100 MHz
130.27
128.65
127.07
126.18
125.78
125.74
122.79
173.20
140.71
139.93
78.23
73.54
72.82
48.86
43.57
35.78
35.12
f1 (ppm)

SI-4 19F

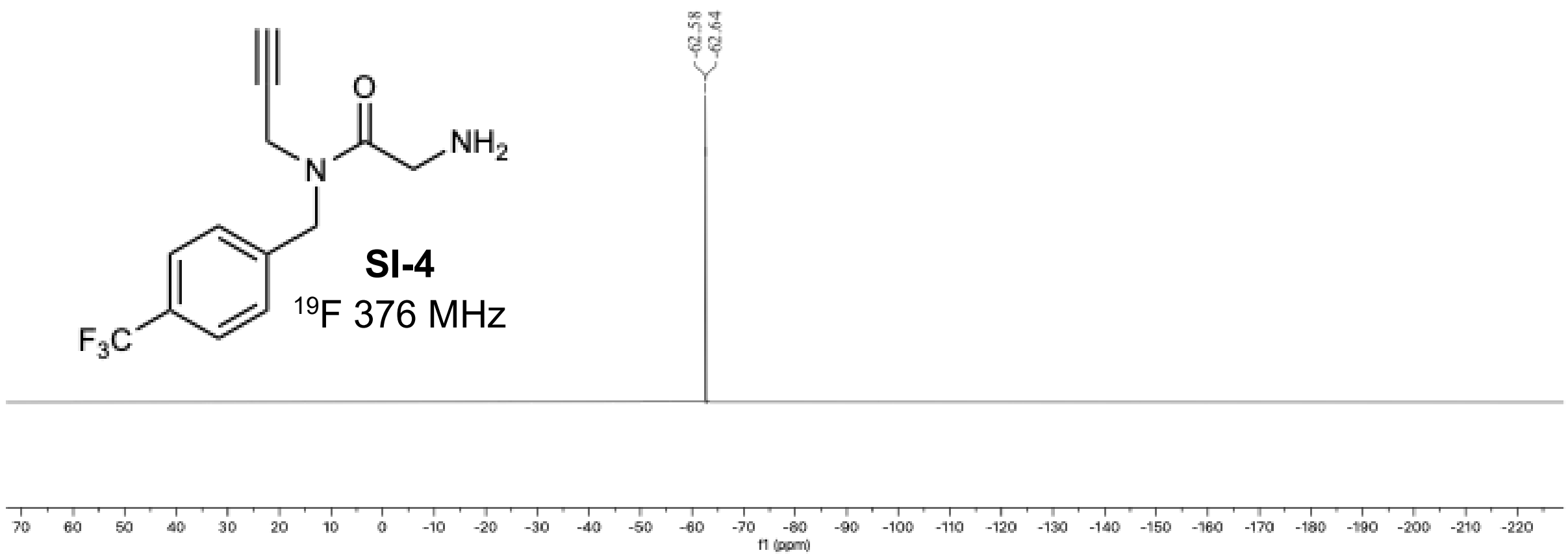

-62.58
-62.64
SI-4
19F 376 MHz
70 60 50 40 30 20 10 0 -10 -20 -30 -40 -50 -60 -70 -80 -90 -100 -110 -120 -130 -140 -150 -160 -170 -180 -190 -200 -210 -220
f1 (ppm)

CA-Ind-Alk 1H

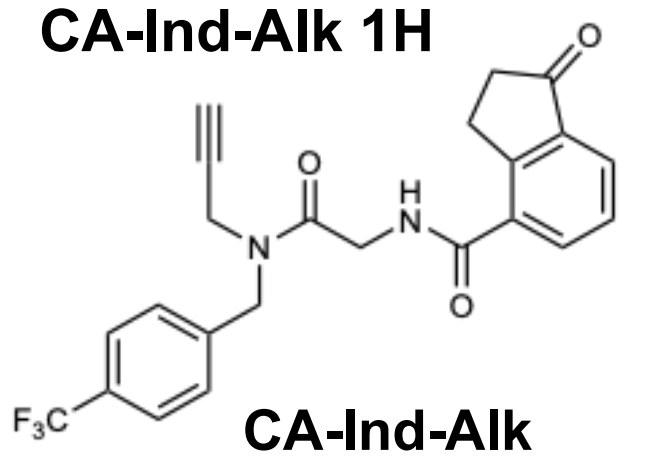

CA-Ind-Alk

^{1}H 600 MHz

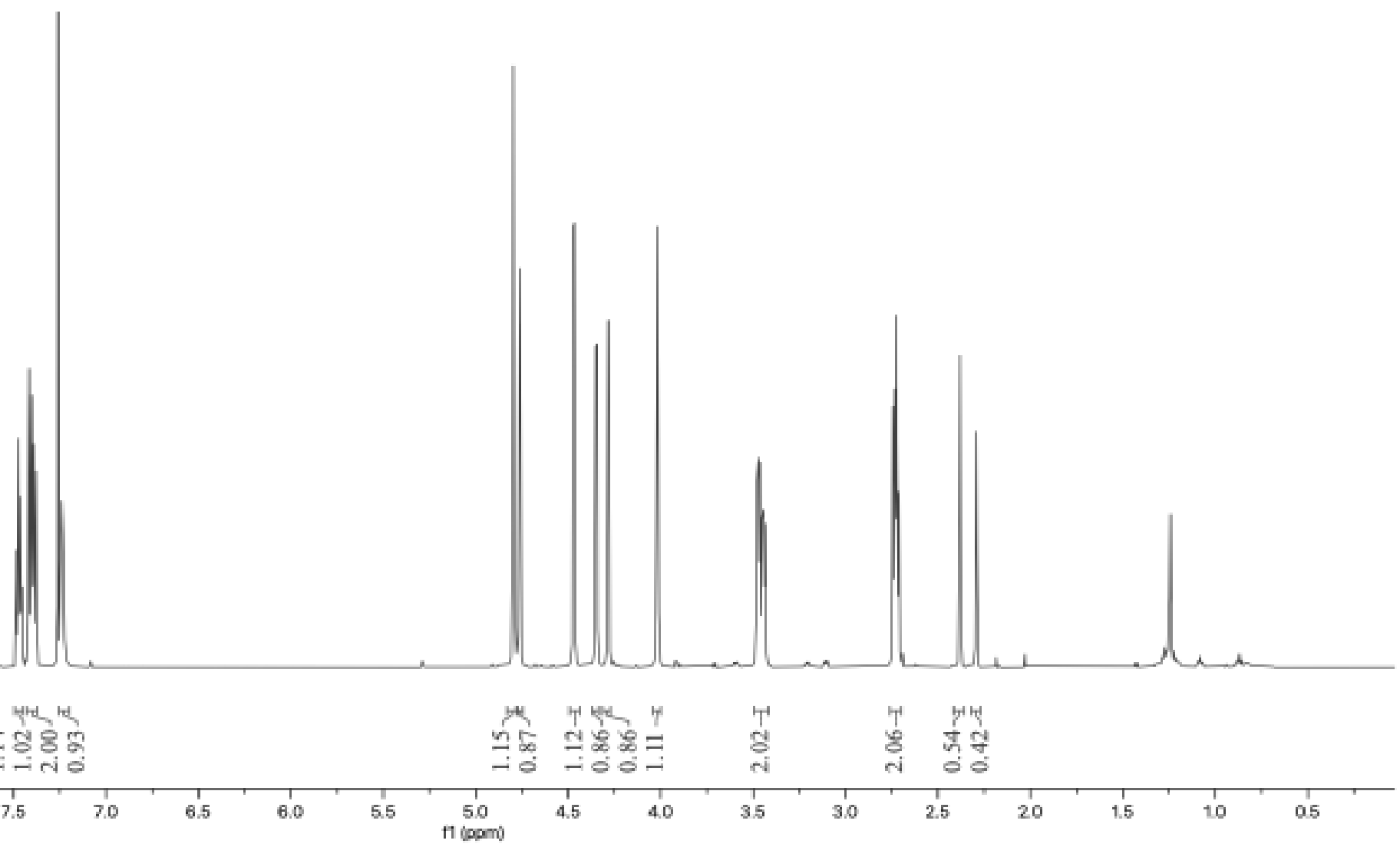

CA-Ind-Alk 13C

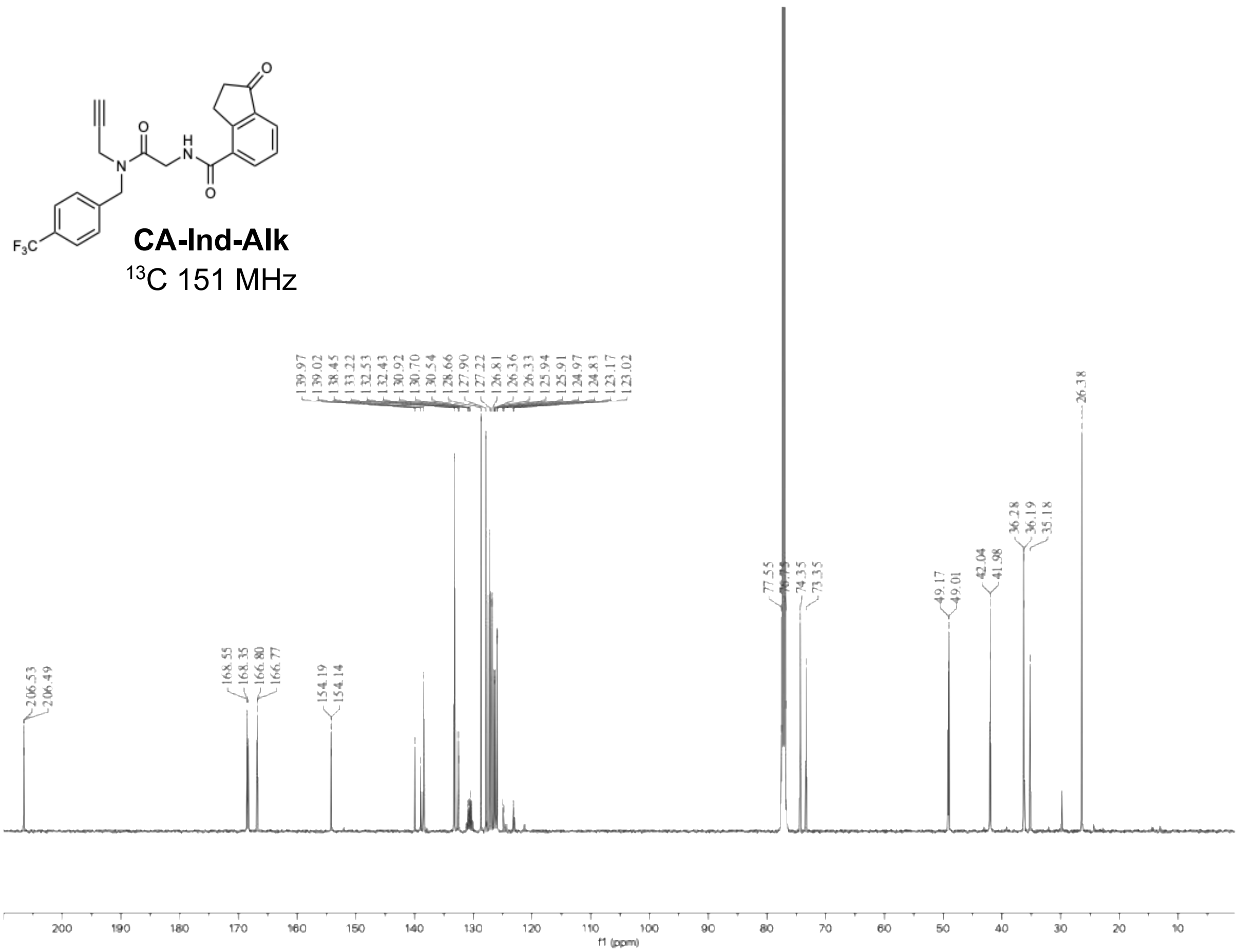

CA-Ind-Alk 19F

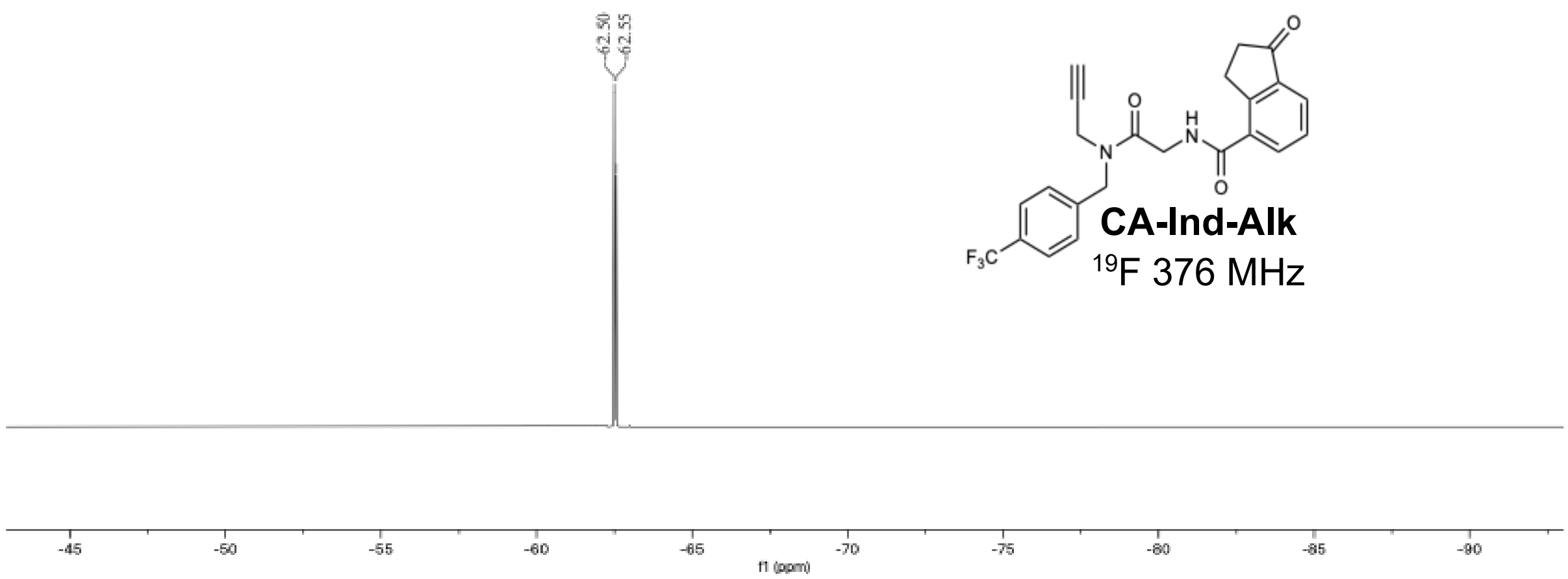

SI-6 1H

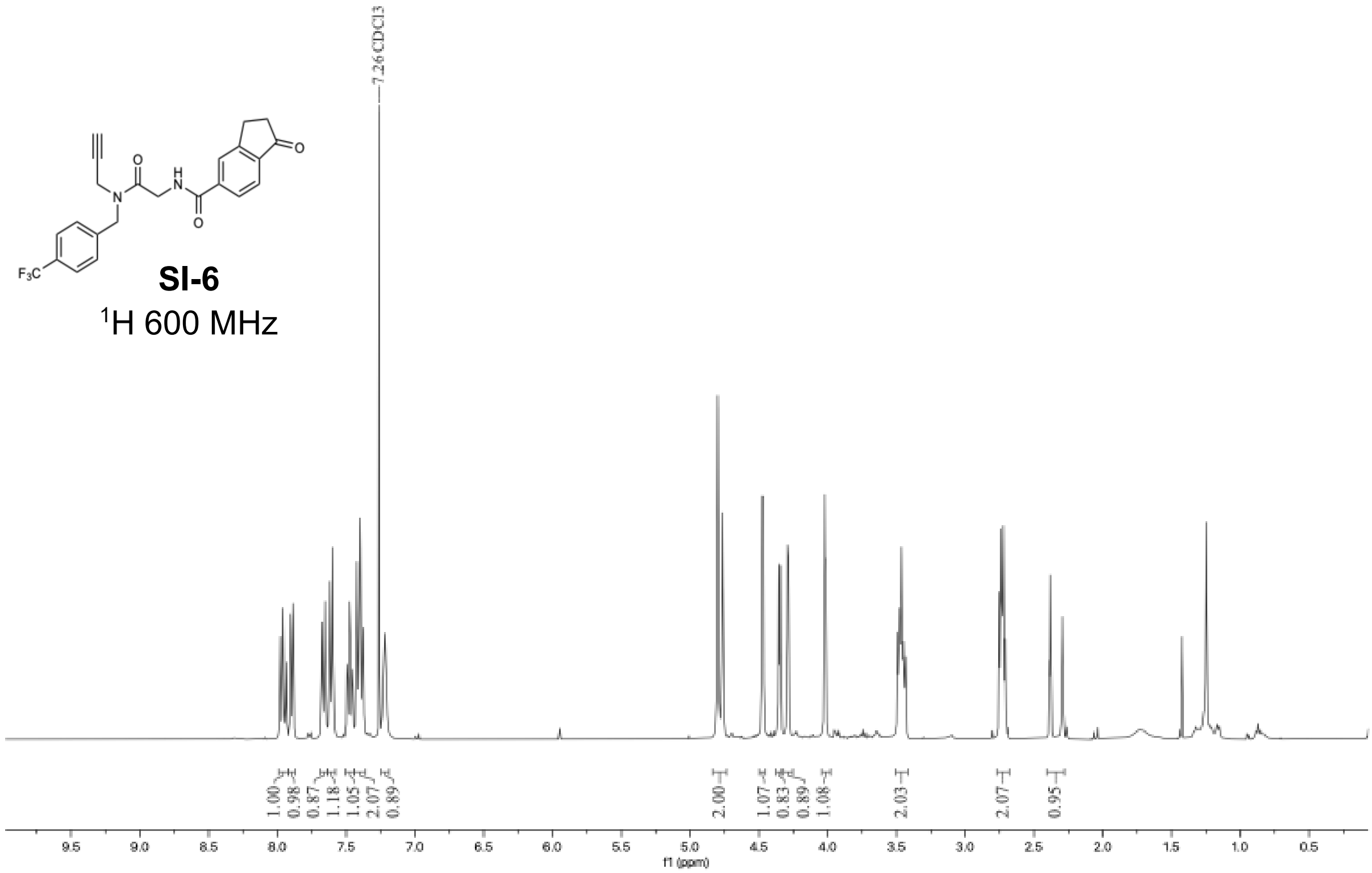

SI-6 13C

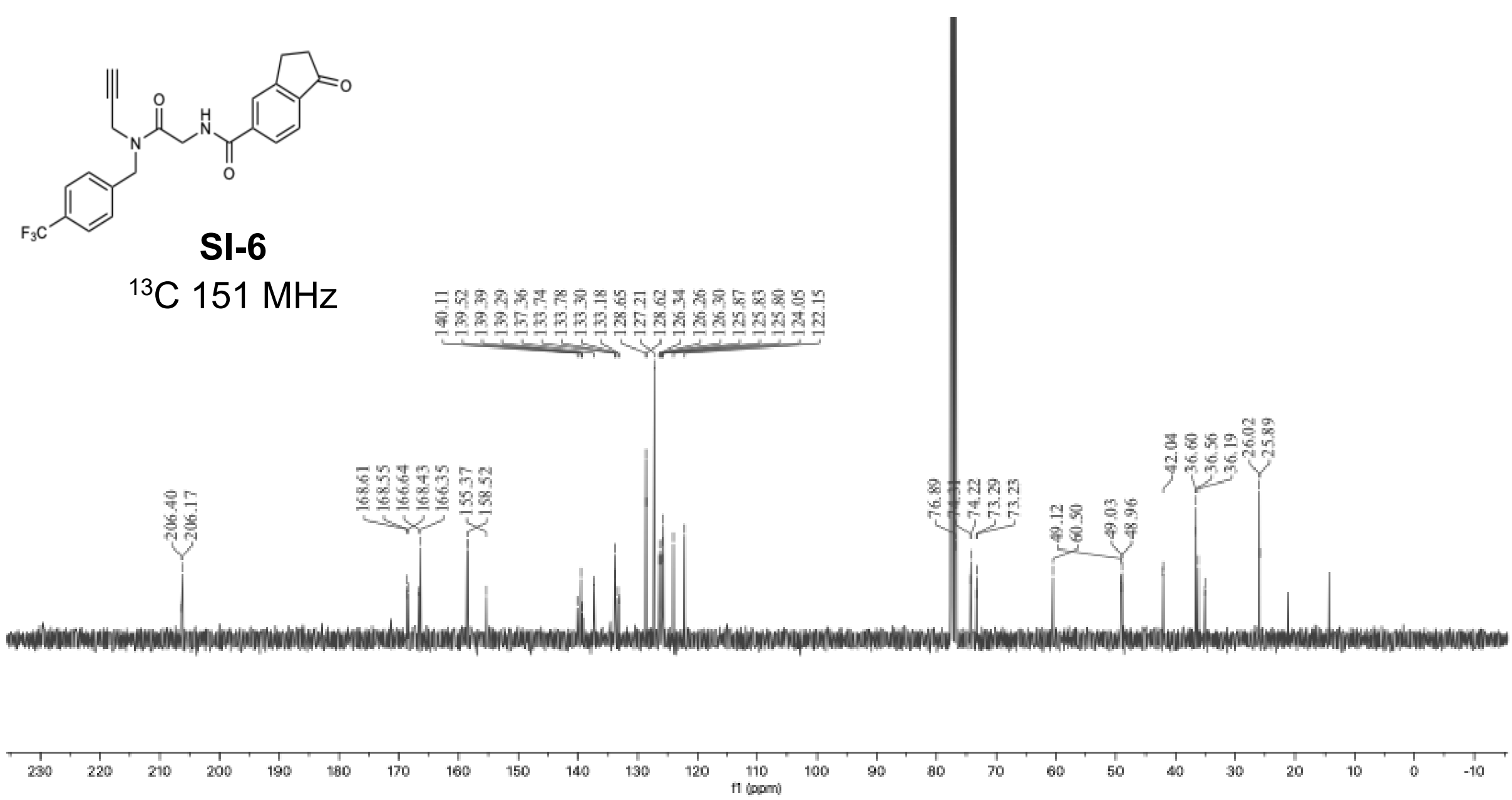

SI-6 19F

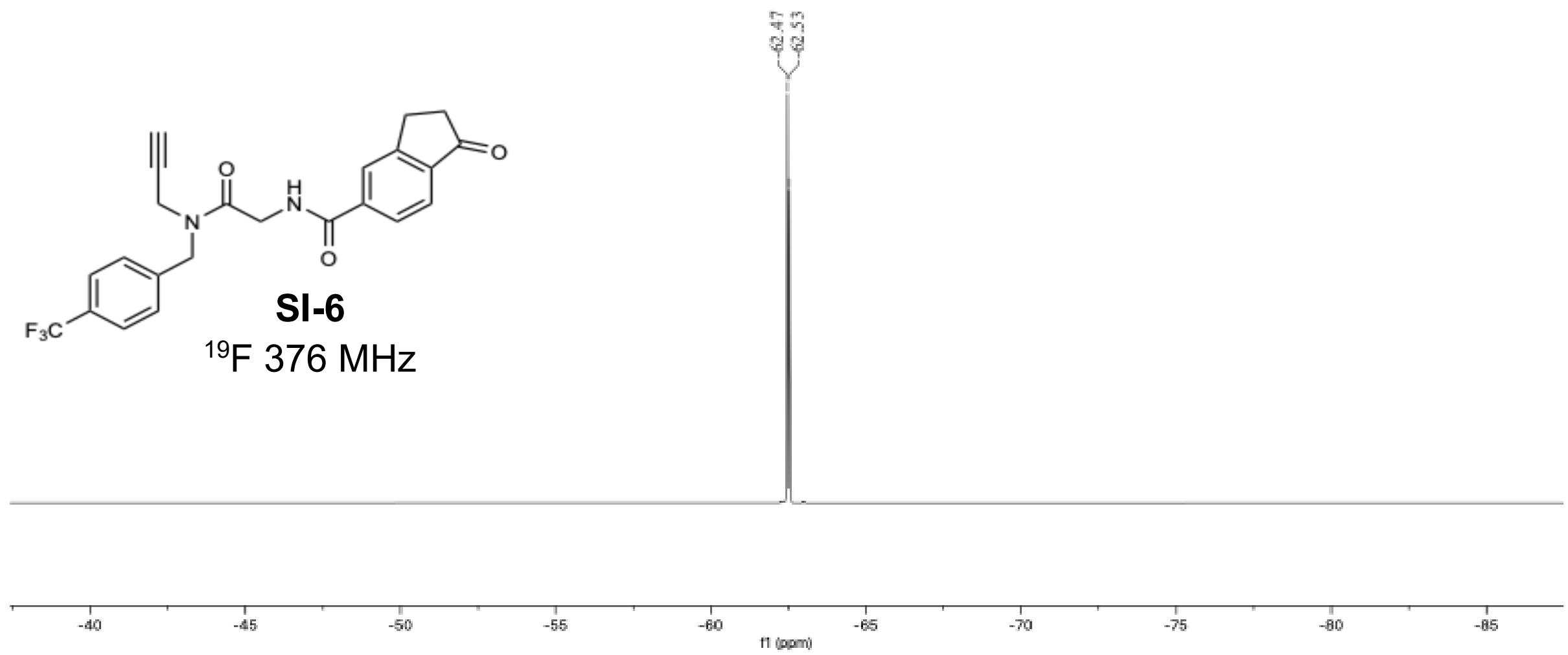
-62.47
-62.53
O
H
N
N
O
O
F3C
SI-6
19F 376 MHz
-40
-45
-50
-55
-60
-65
-70
-75
-80
-85
f1 (ppm)

isoCA-Alk 1H

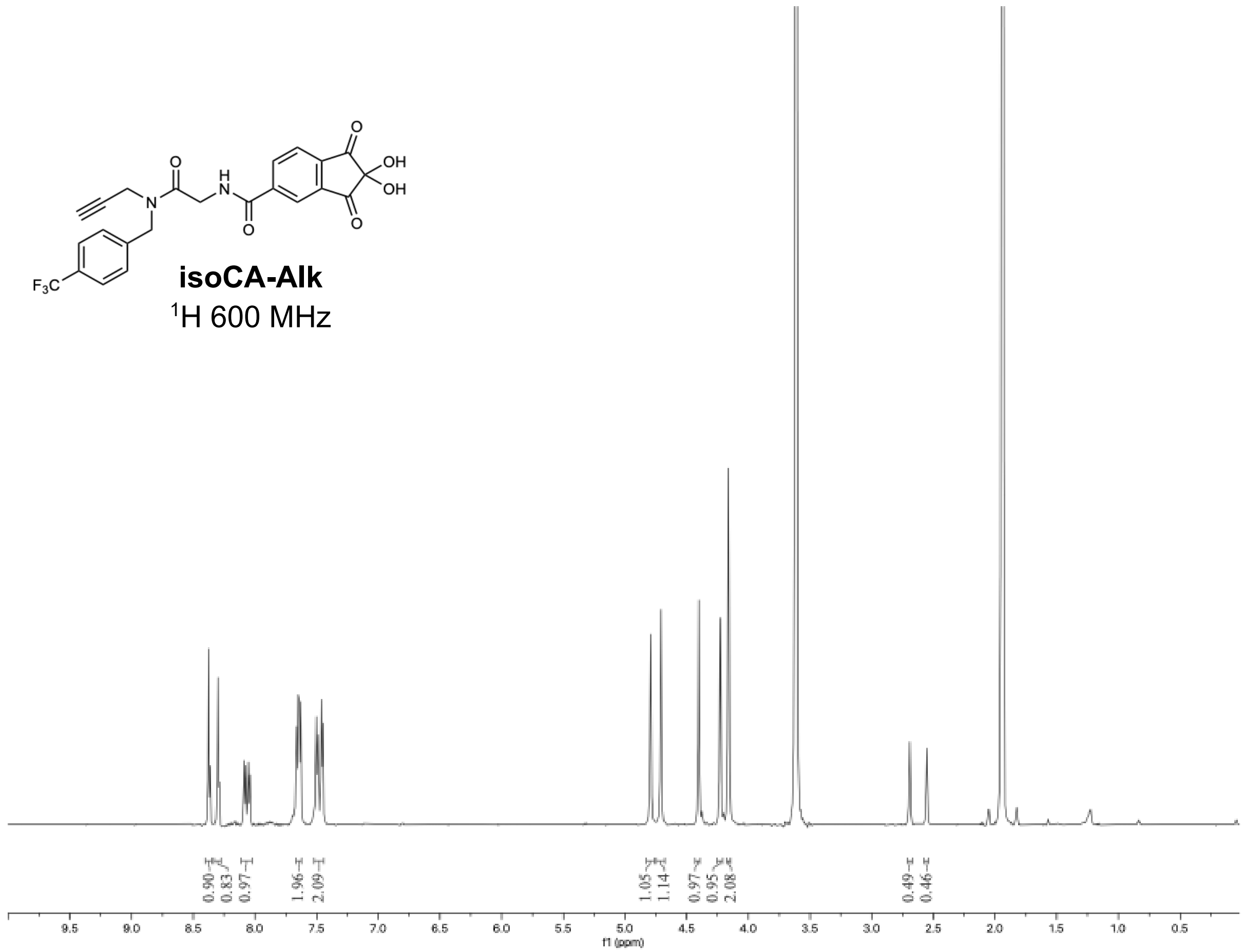

isoCA-Alk

^{1}H 600 MHz

isoCA-Alk 13C

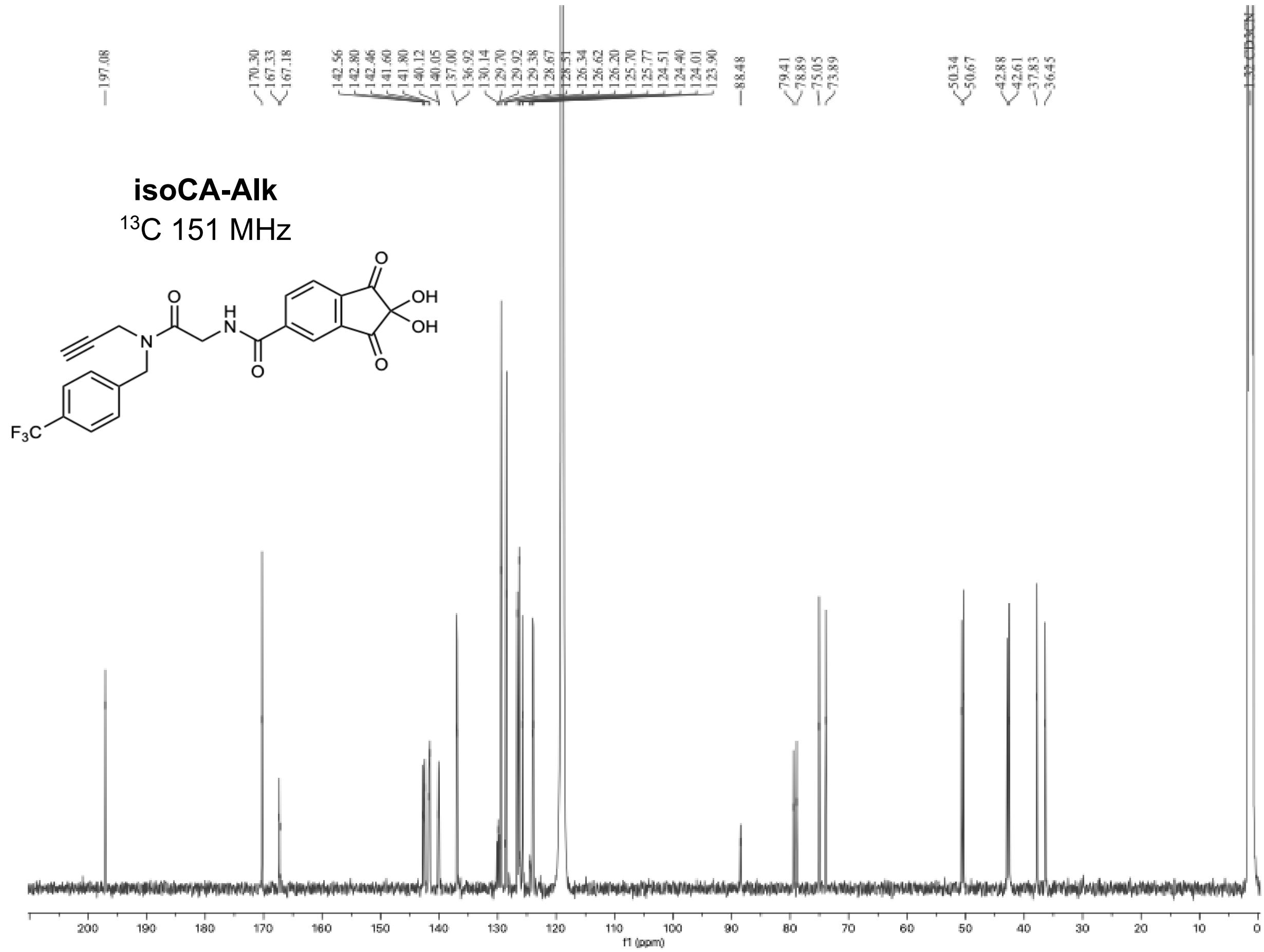

isoCA-Alk 19F

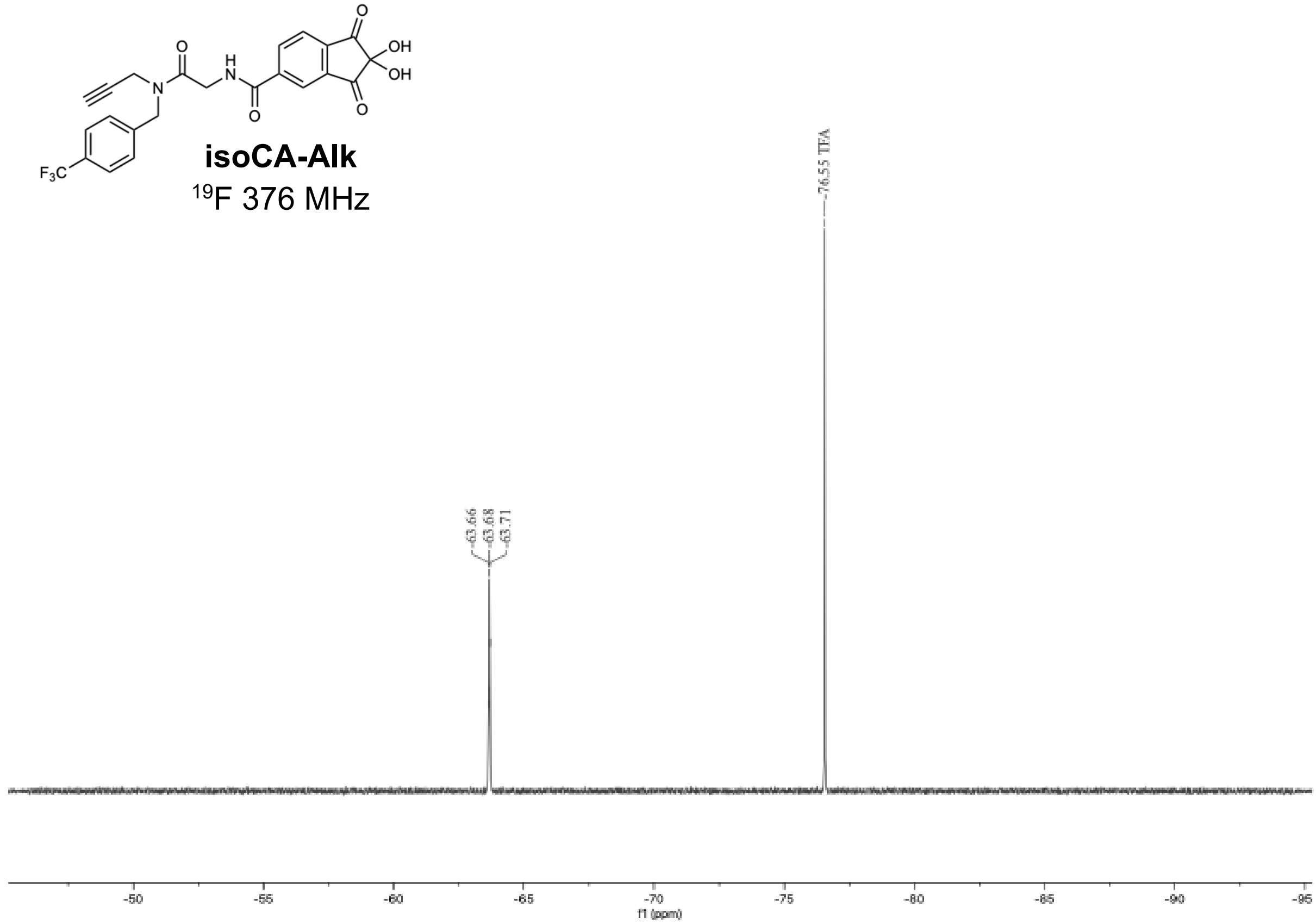

isoCA-Alk HSQC

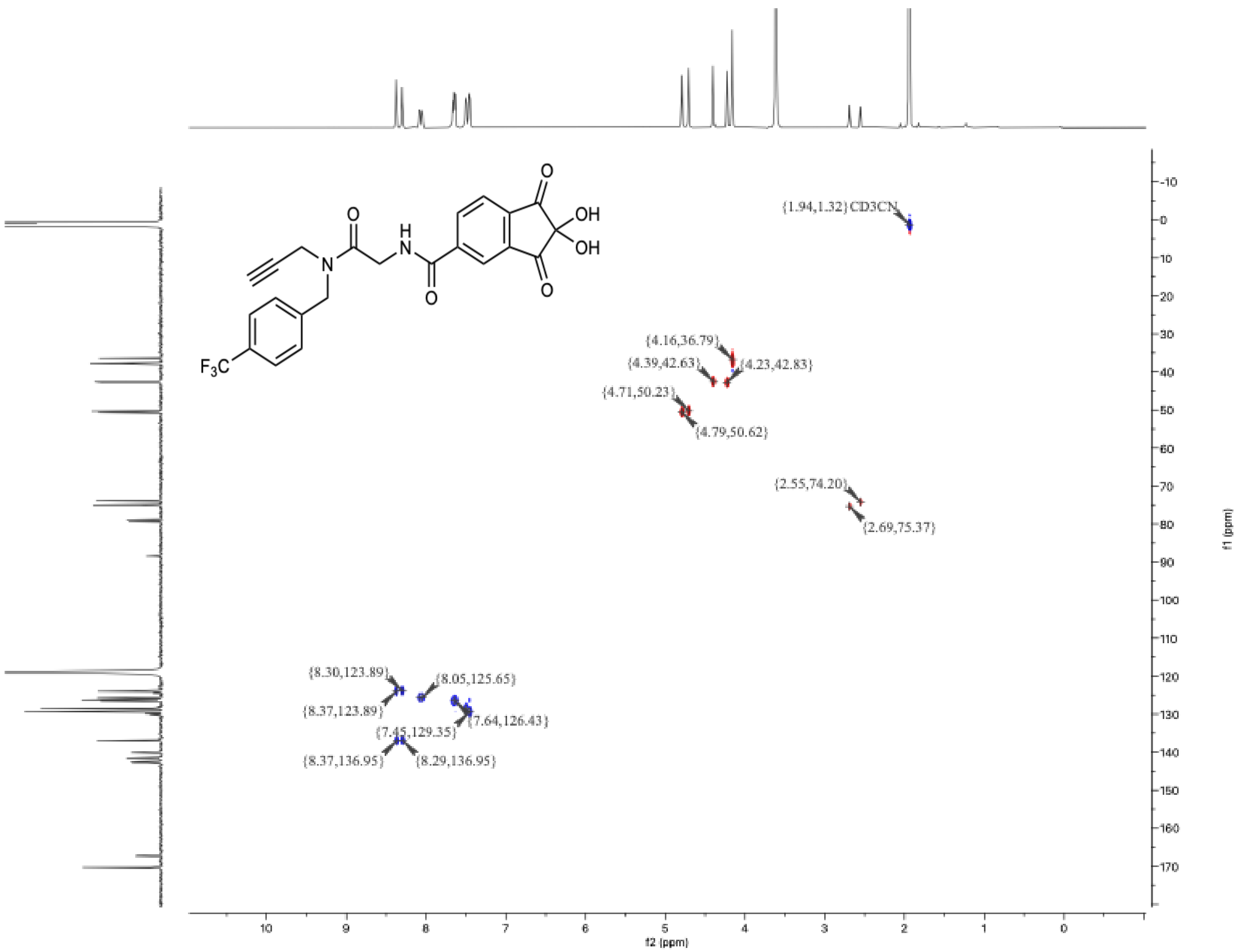

CA-Alk 1H

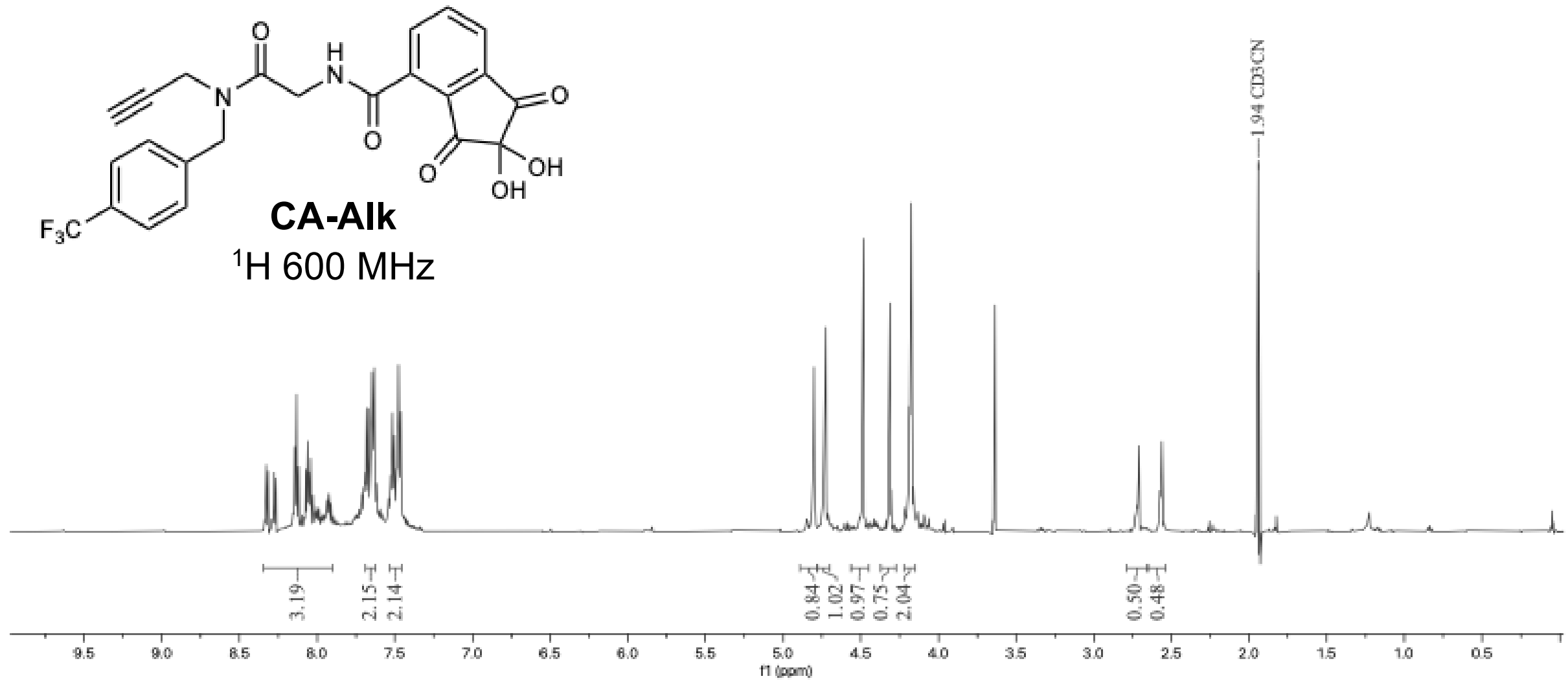

CA-Alk 13C

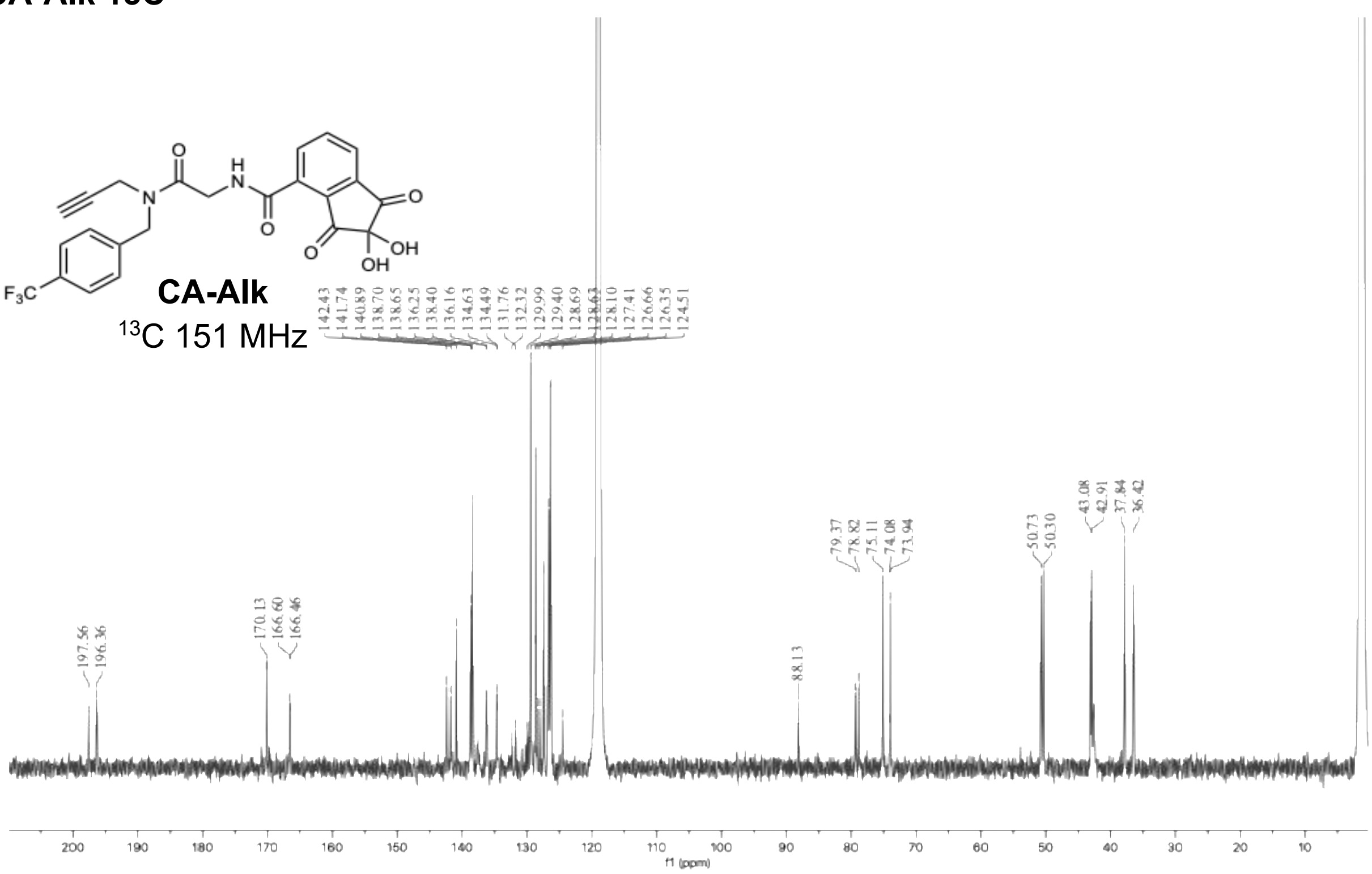

CA-Alk 19F

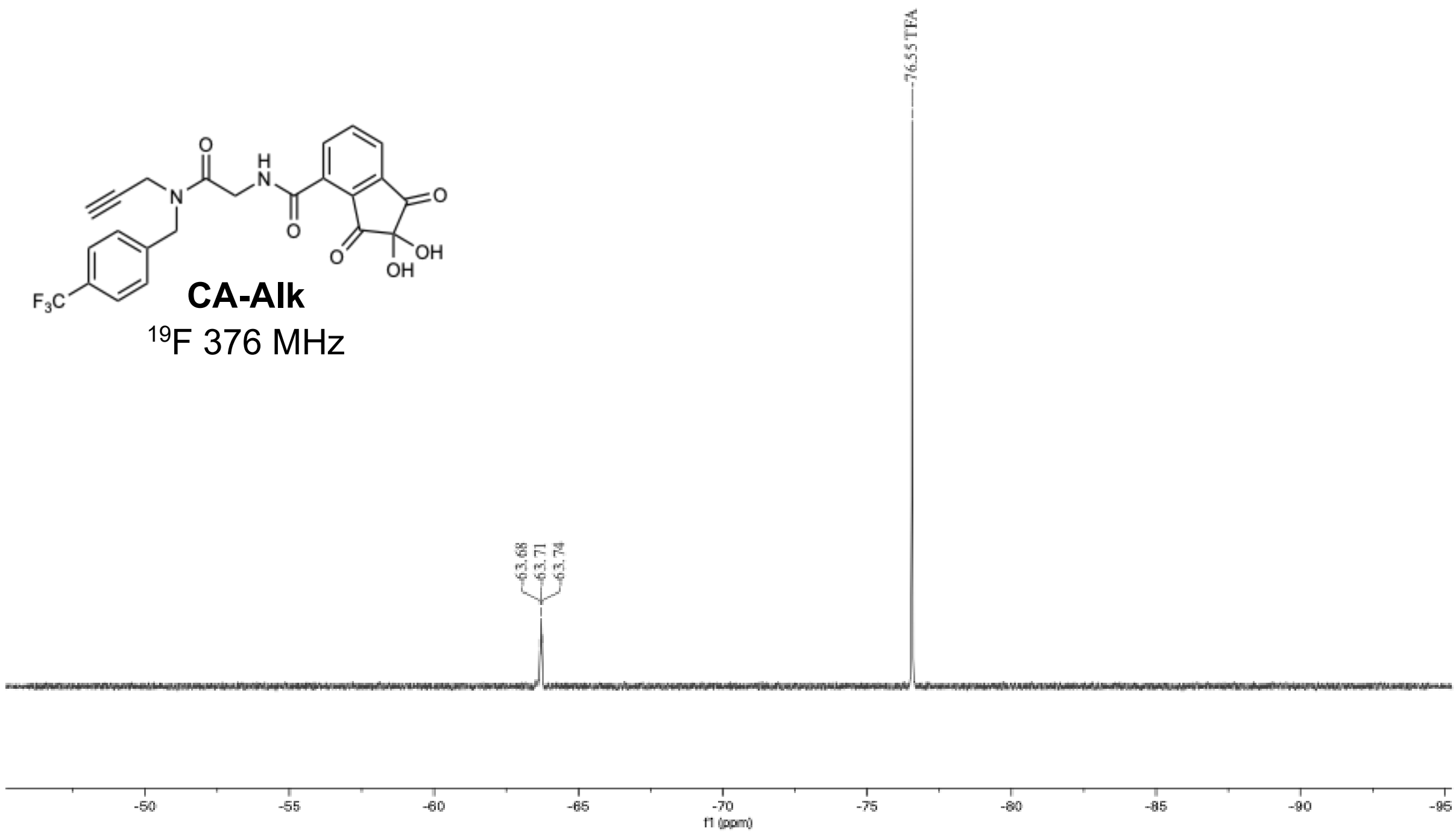

O
H
N
O
OH
OH
F3C
CA-Alk
19F 376 MHz
-63.68
-63.71
-63.74
-76.55 TFA
-50
-55
-60
-65
-70
-75
-80
-85
-90
-95
f1 (ppm)

CA-Alk HSQC

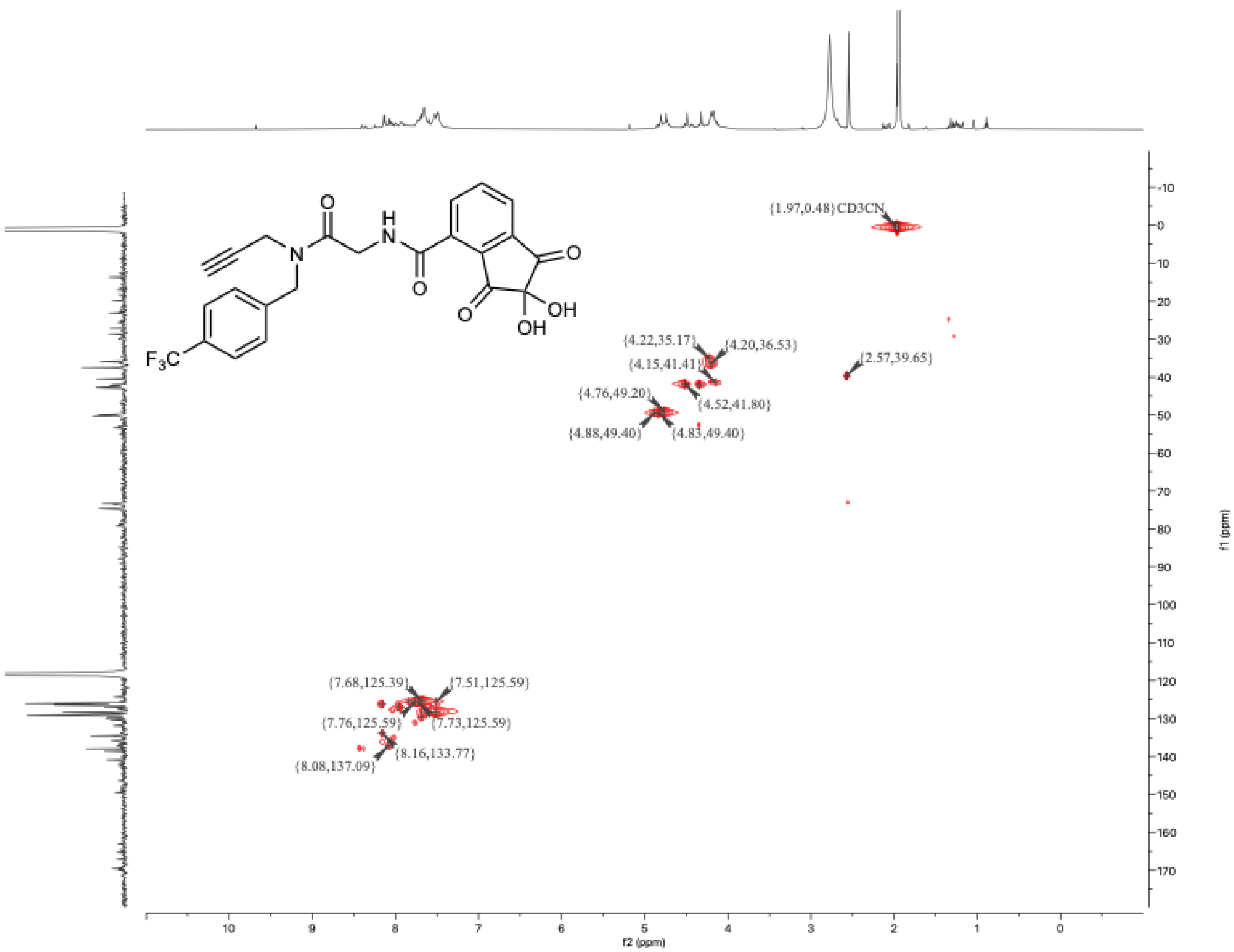
{1.97,0.48}CD3CN
{4.22,35.17}
{4.20,36.53}
{4.15,41.41}
{2.57,39.65}
{4.76,49.20}
{4.52,41.80}
{4.88,49.40}
{4.83,49.40}
{7.68,125.39}
{7.51,125.59}
{7.76,125.59}
{7.73,125.59}
{8.16,133.77}
{8.08,137.09}
f2 (ppm)
f1 (ppm)

CA-Alk HMBC

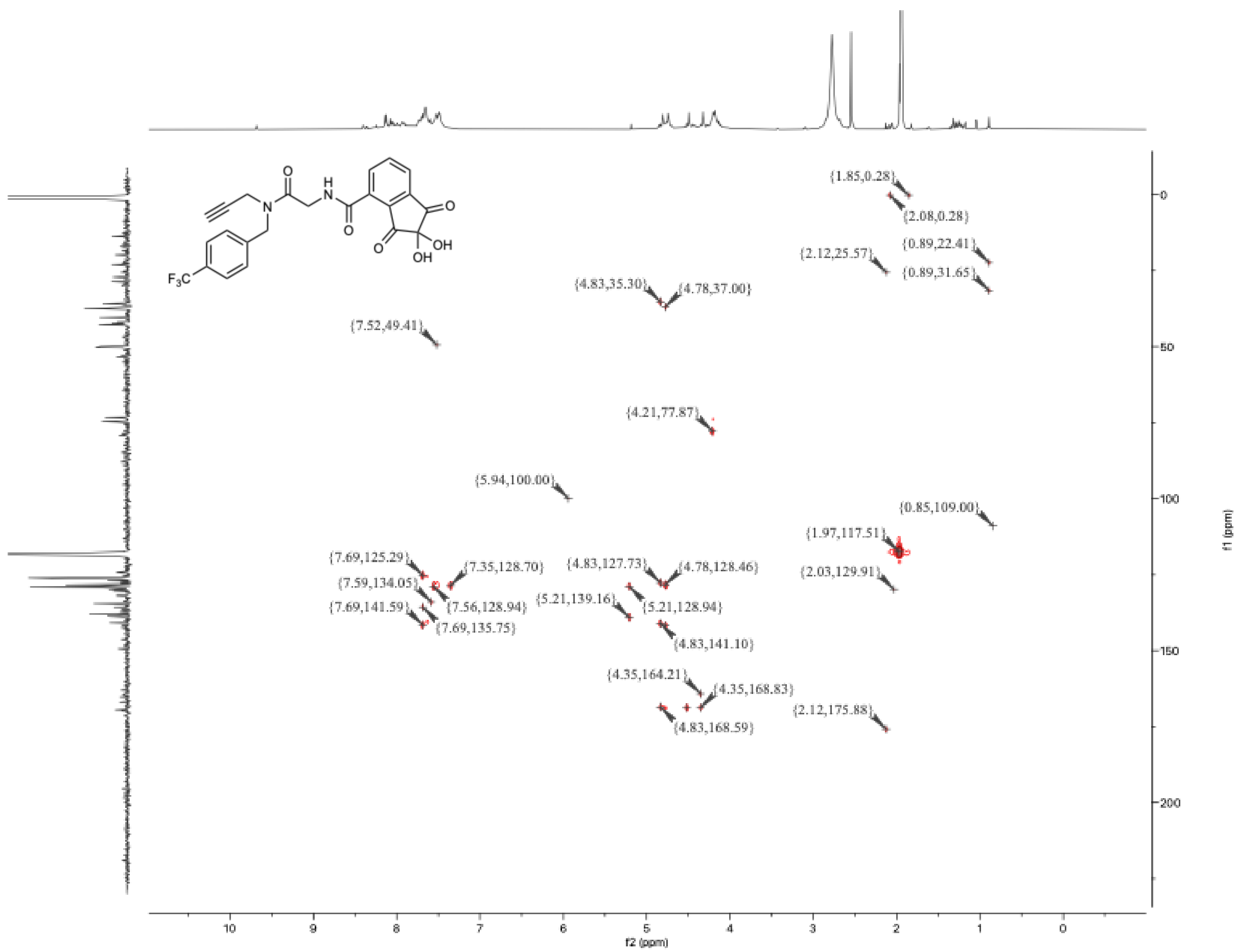
{1.85,0.28}
{2.08,0.28}
{0.89,22.41}
{2.12,25.57}
{0.89,31.65}
{4.83,35.30}
{4.78,37.00}
{7.52,49.41}
{4.21,77.87}
{5.94,100.00}
{0.85,109.00}
{1.97,117.51}
{7.69,125.29}
{7.35,128.70}
{4.83,127.73}
{4.78,128.46}
{2.03,129.91}
{7.59,134.05}
{7.56,128.94}
{5.21,139.16}
{5.21,128.94}
{7.69,141.59}
{7.69,135.75}
{4.83,141.10}
{4.35,164.21}
{4.35,168.83}
{2.12,175.88}
{4.83,168.59}
f2 (ppm)
f1 (ppm)

Compound SI-7 1H

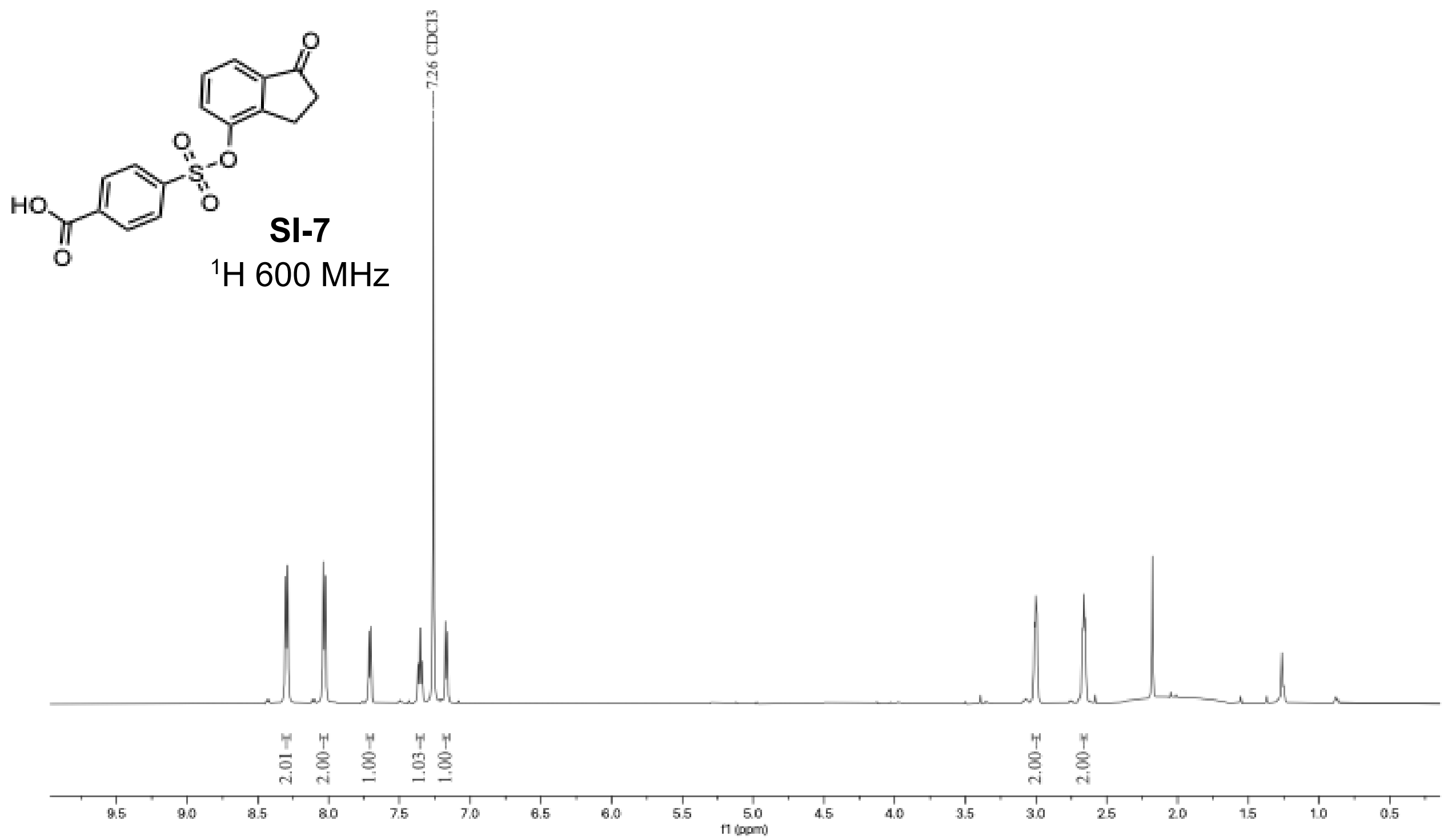

Compound SI-7 13C

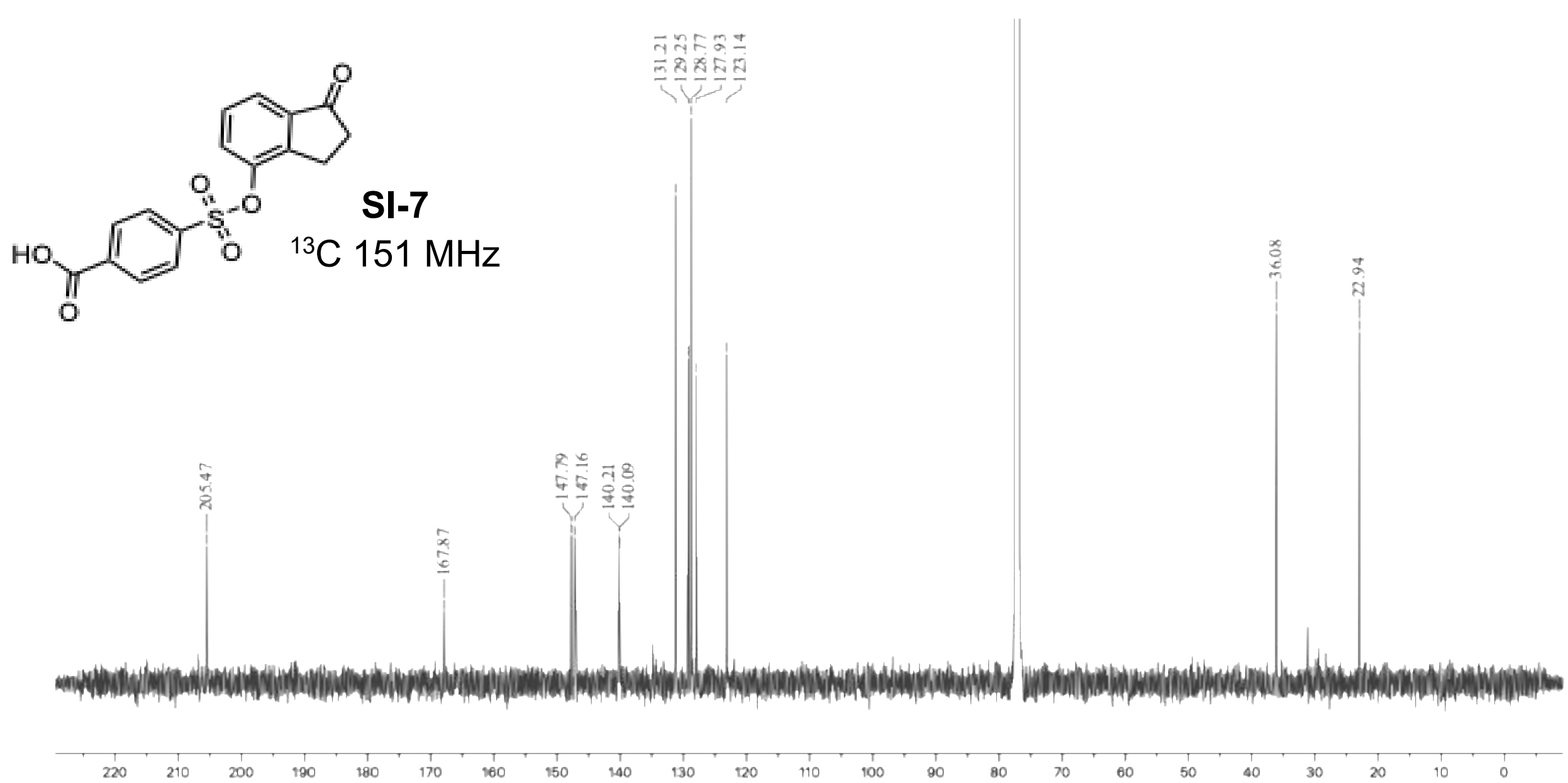

Compound SI-8 1H

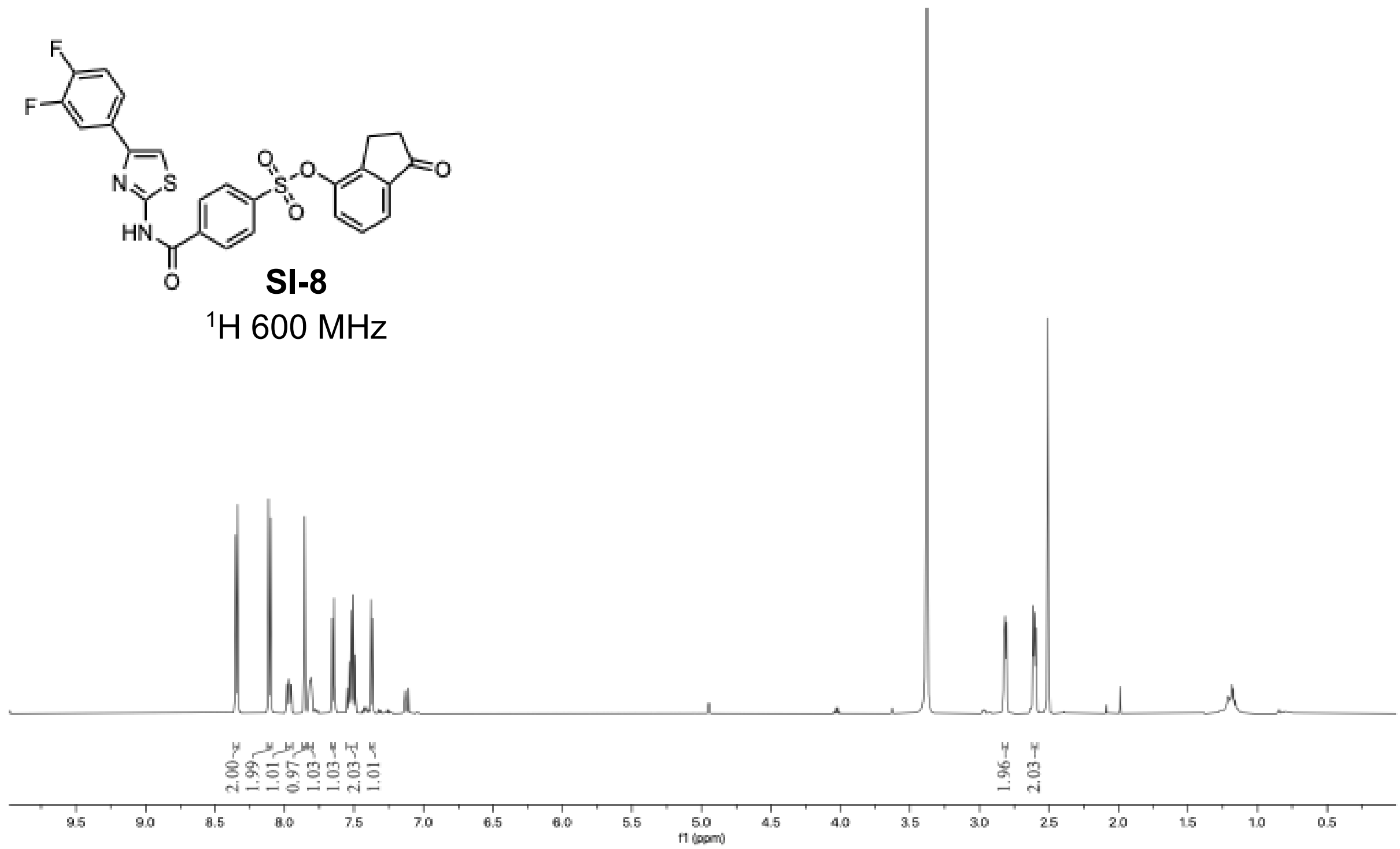

Compound SI-8 13C

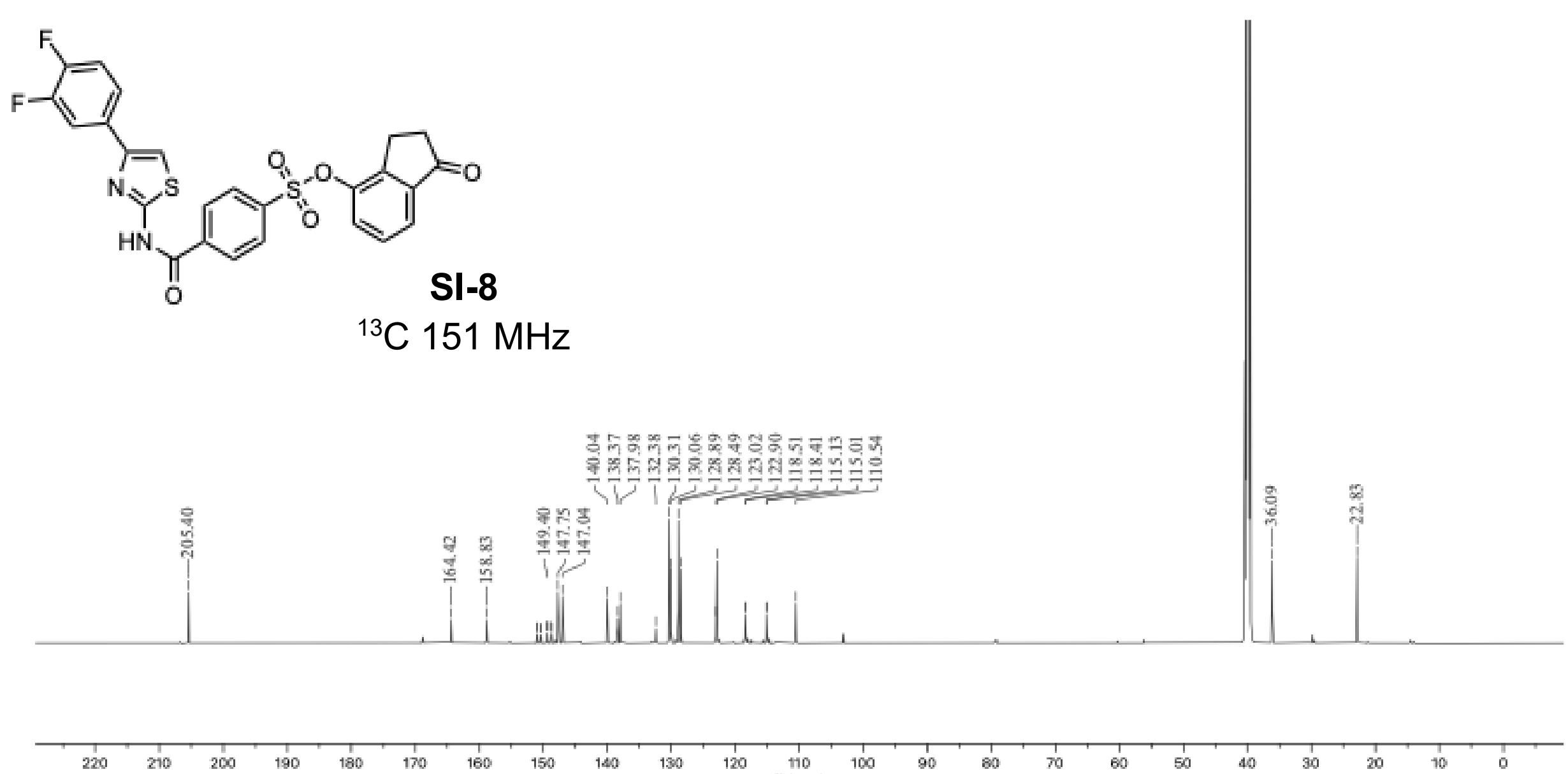

Lig-Nin 1H

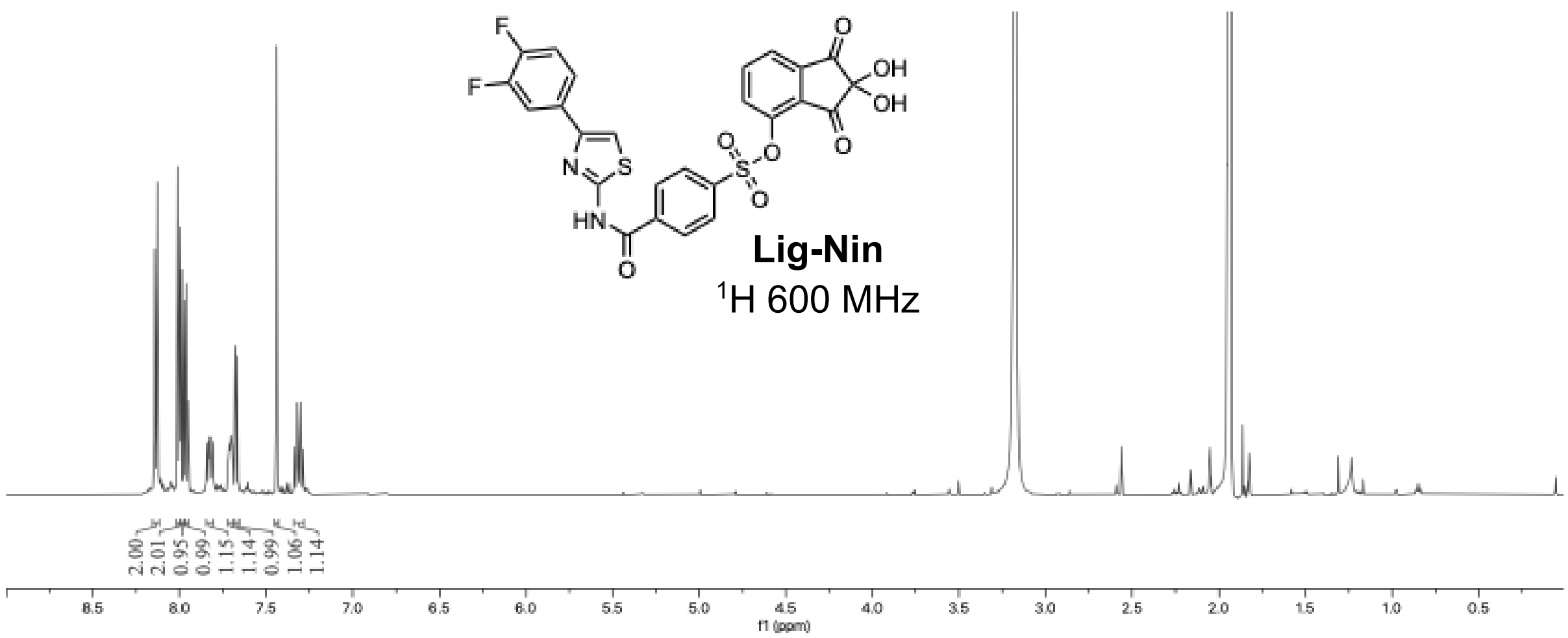

Lig-Nin 13C

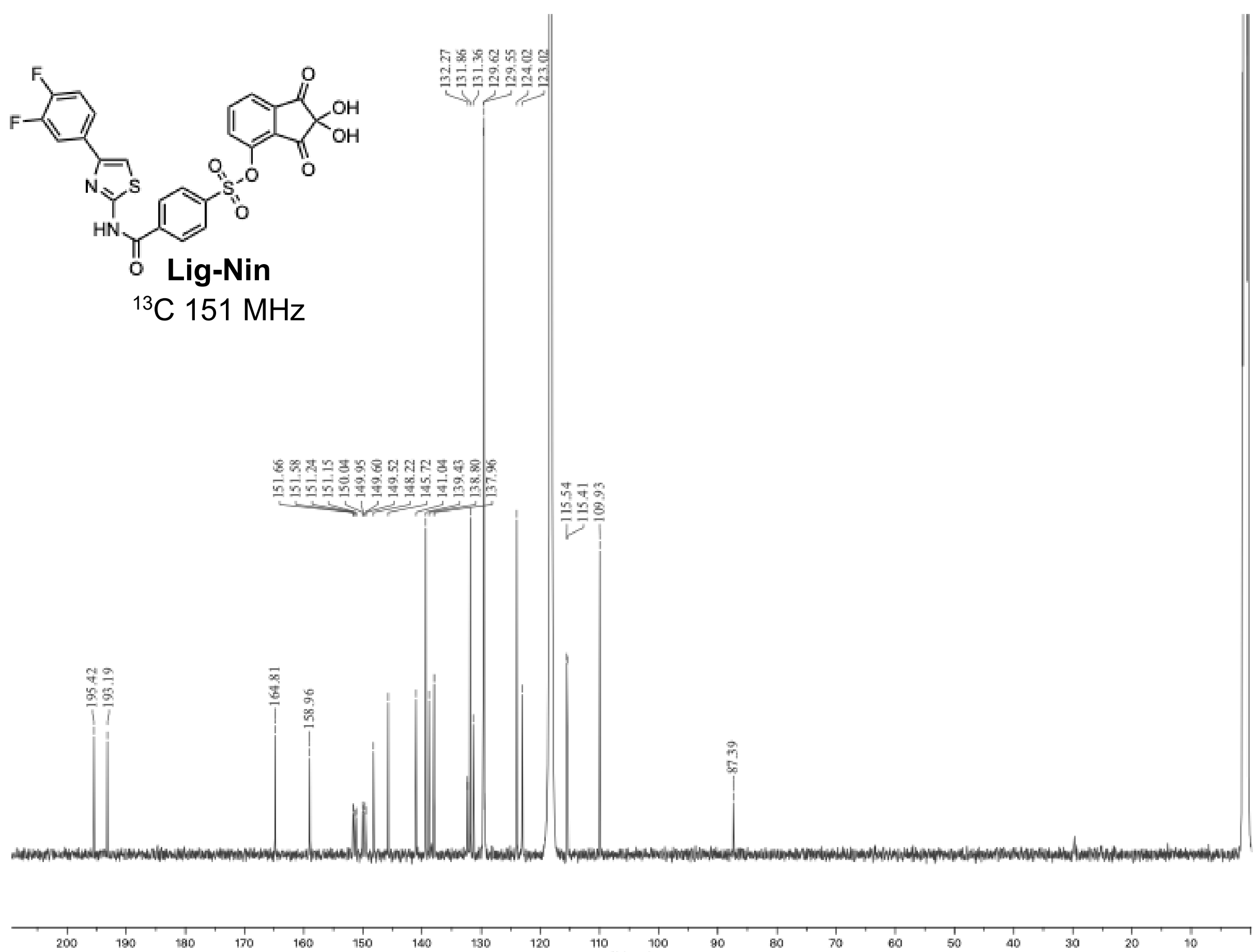

Lig-Nin 19F

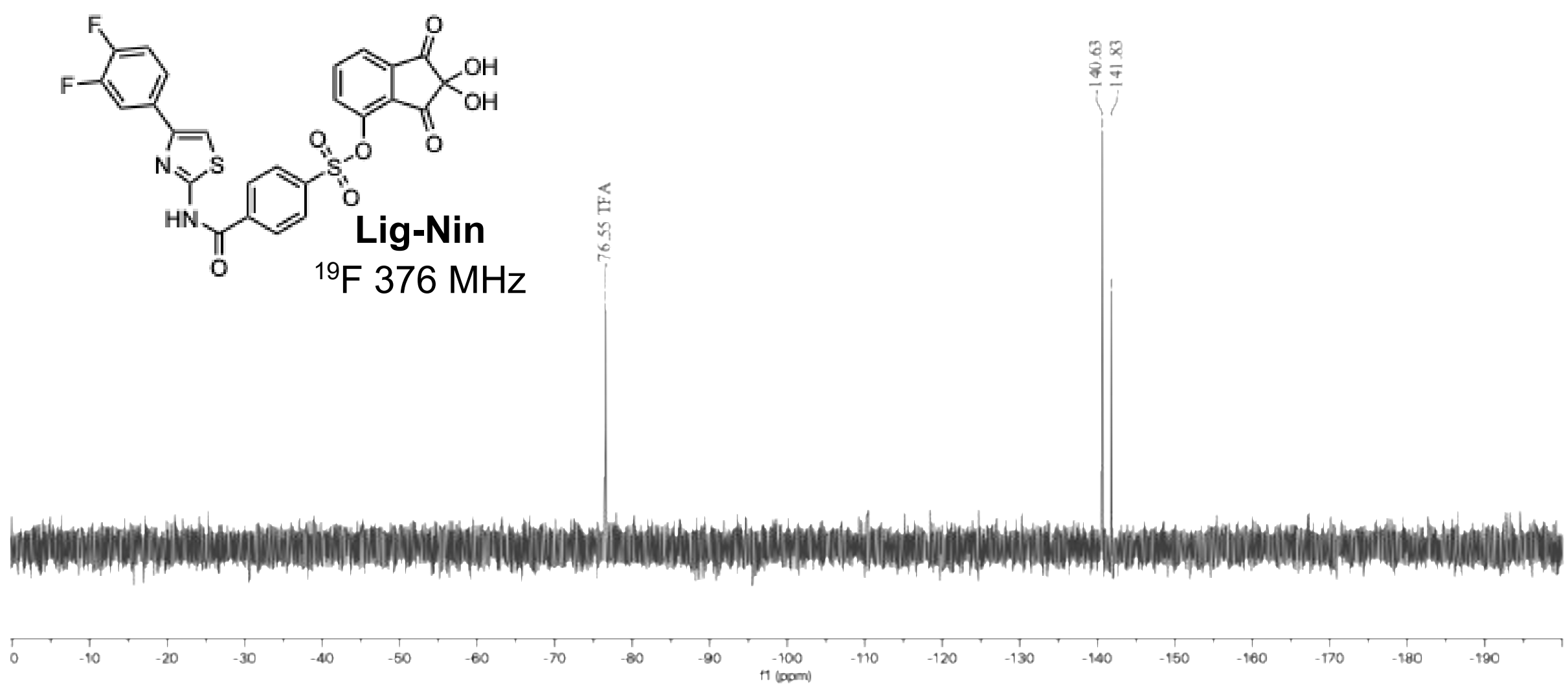

Lig-Nin COSY

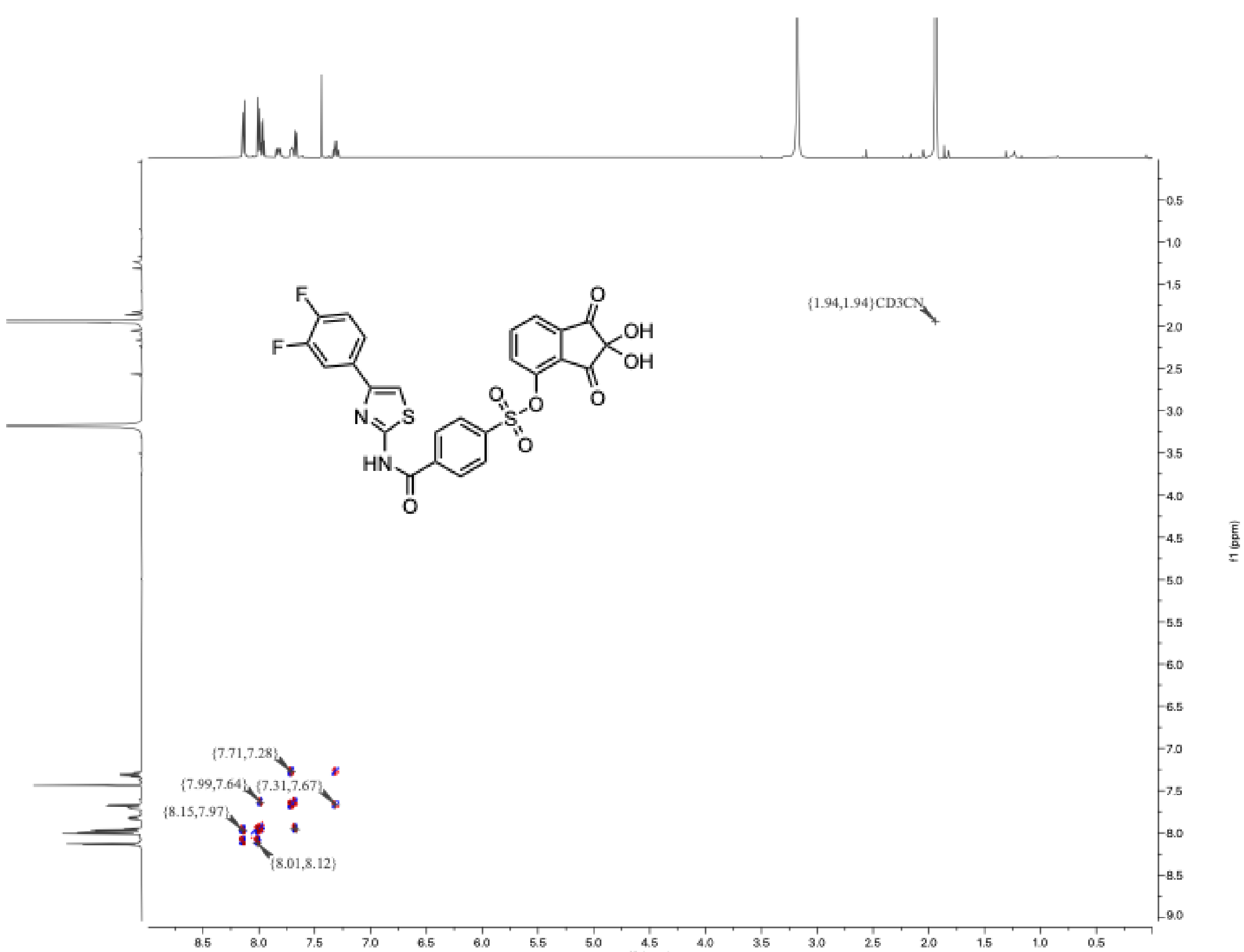

Lig-Nin HSQC

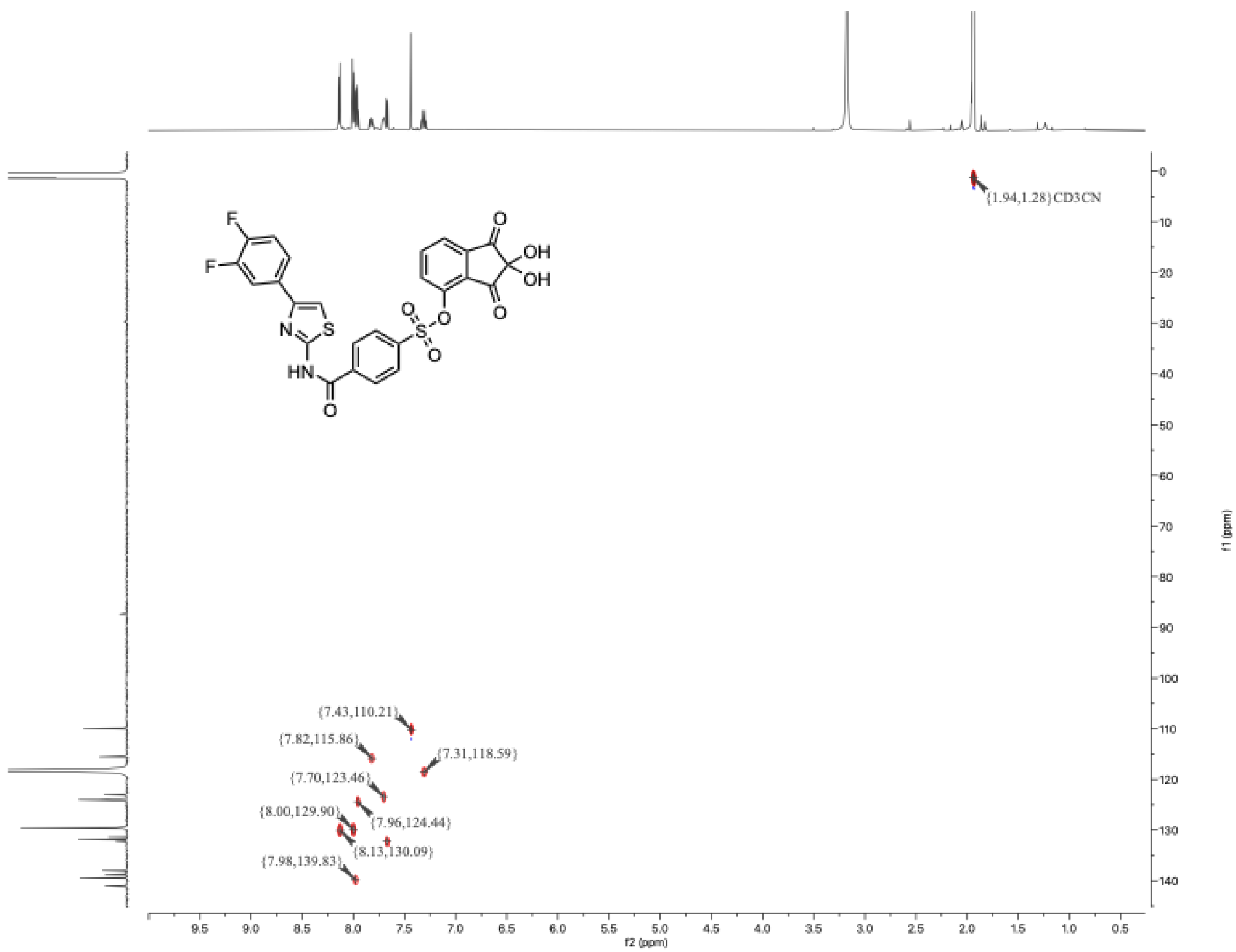
{1.94,1.28}CD3CN
F
F
N
S
HN
O
O
S
O
O
O
OH
OH
O
{7.43,110.21}
{7.82,115.86}
{7.31,118.59}
{7.70,123.46}
{8.00,129.90}
{7.96,124.44}
{8.13,130.09}
{7.98,139.83}
f1 (ppm)
f2 (ppm)

CA-Alk: HPLC purity trace 254 nm

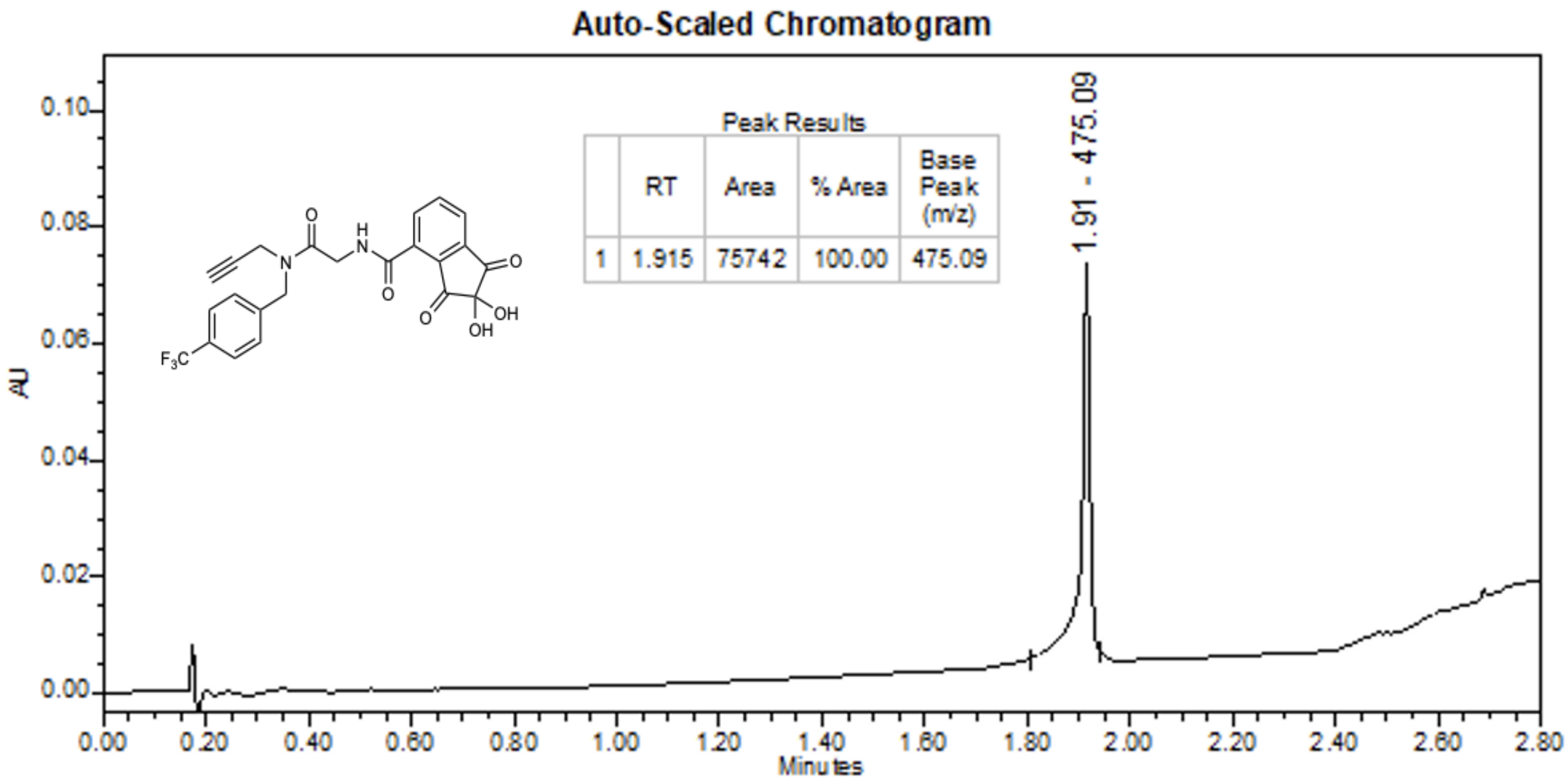

	RT	Area	% Area	Base Peak (m/z)
1	1.915	75742	100.00	475.09

Iso-CA-Alk: HPLC purity trace 254 nm

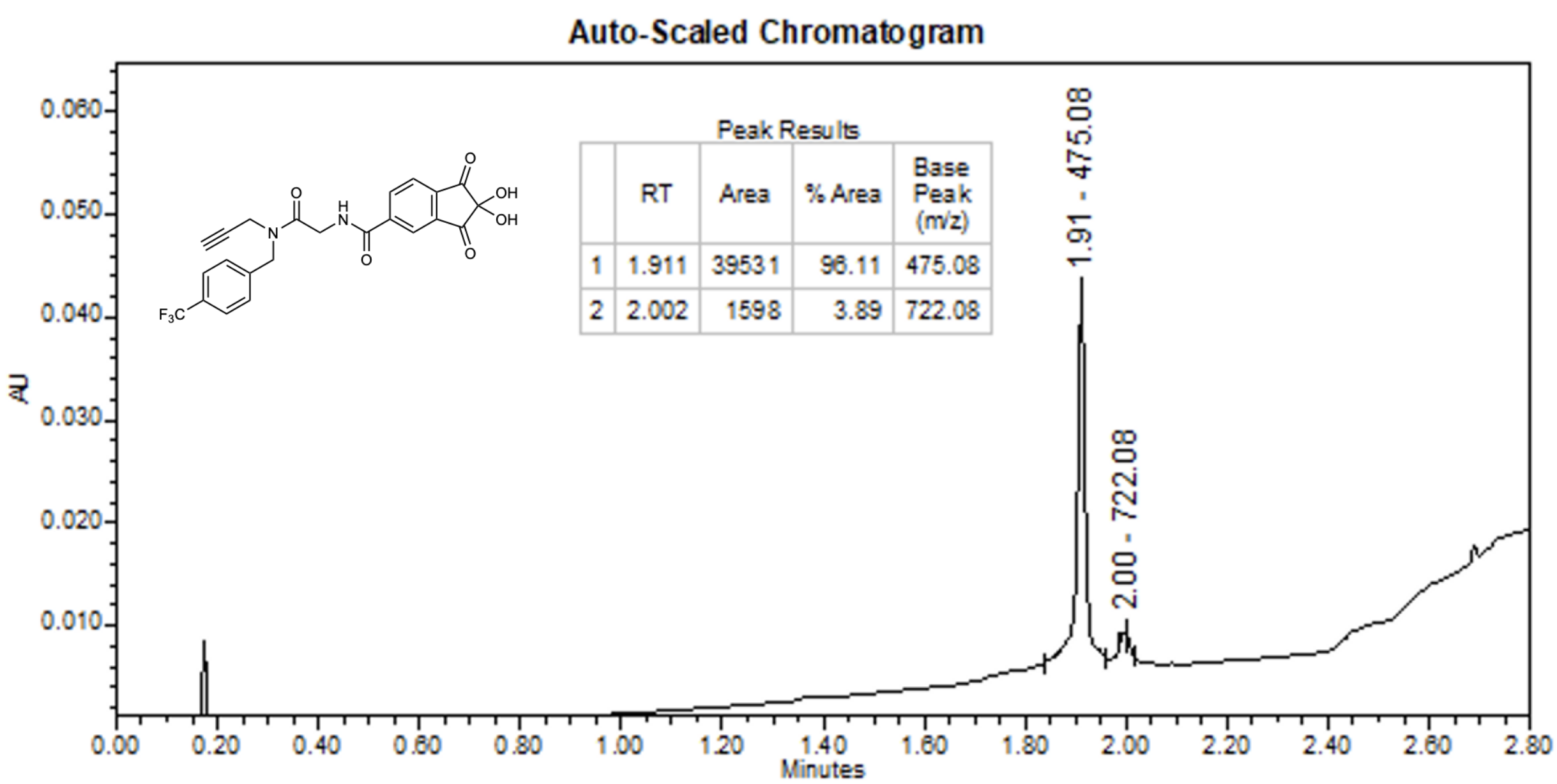

	RT	Area	% Area	Base Peak (m/z)
1	1.911	39531	96.11	475.08
2	2.002	1598	3.89	722.08

Lig-Nin: HPLC purity trace 254 nm

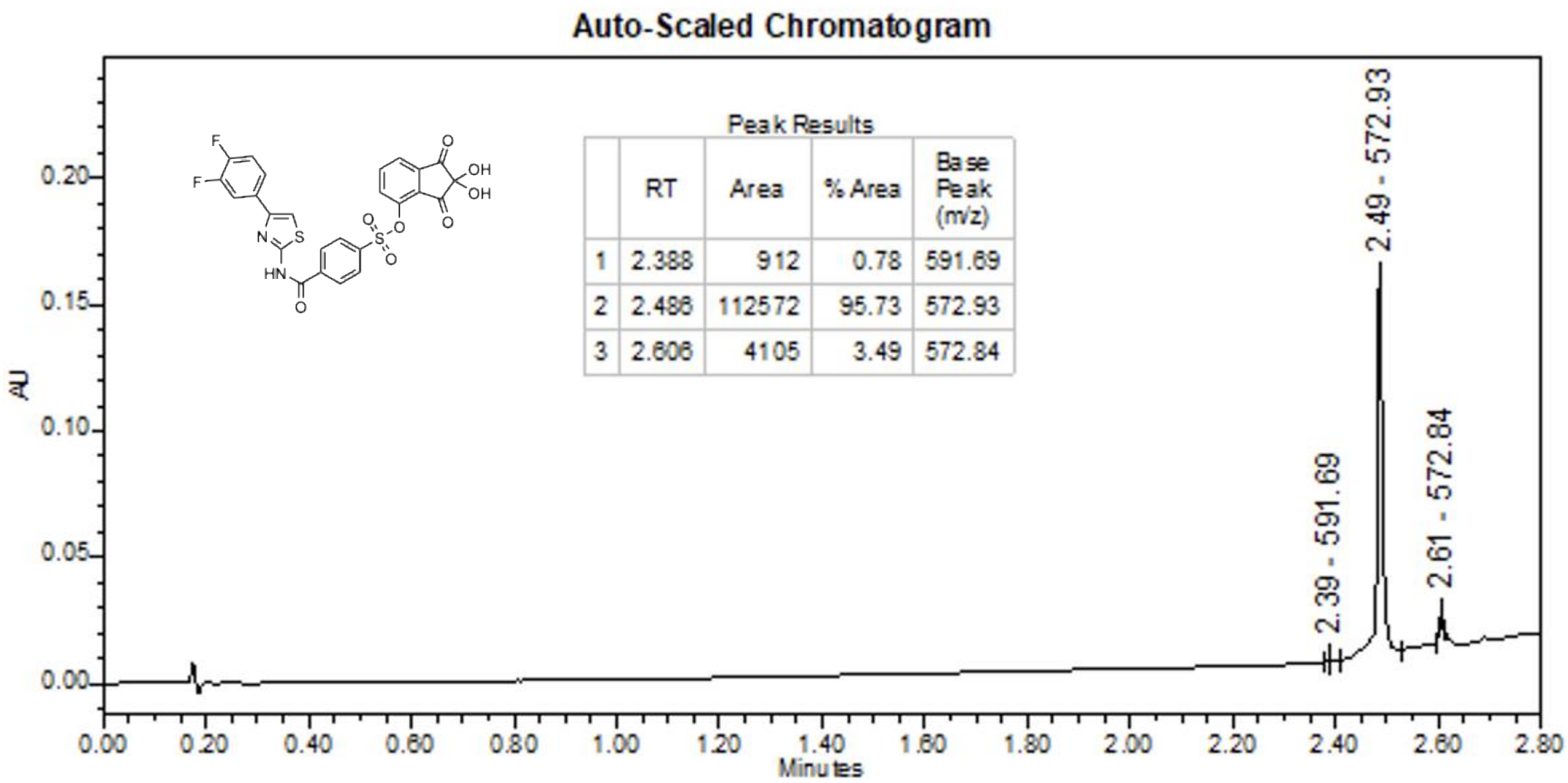

	RT	Area	% Area	Base Peak (m/z)
1	2.388	912	0.78	591.69
2	2.486	112572	95.73	572.93
3	2.606	4105	3.49	572.84
